# Rh(iii)-catalyzed synthesis of tetracyclic isoquinolinium salts *via* C–H activation and [4+2] annulation of 1-phenyl-3,4-dihydroisoquinolines and alkynes in ethanol†

**DOI:** 10.1039/c8ra05443f

**Published:** 2018-08-24

**Authors:** Xinxin Dang, Yu He, Yingtian Liu, Xuehong Chen, Jun-Long Li, Xian-Li Zhou, Hezhong Jiang, Jiahong Li

**Affiliations:** School of Life Science and Engineering, Southwest Jiaotong University Chengdu 610041 China jiahongljh@163.com jianghz10@home.swjtu.edu.cn zhouxl@swjtu.edu.cn; Antibiotics Research and Re-evaluation Key Laboratory of Sichuan Province, Sichuan Industrial Institute of Antibiotics, Chengdu University Chengdu 610052 China

## Abstract

An efficient and convenient method to construct tetracyclic isoquinolinium salts *via* [Cp*RhCl_2_]_2_ catalyzed C–H activation and [4 + 2] annulation reactions in ethanol is described. This reaction is very fast and highly efficient in the green solvent ethanol. The reaction works with a broad substrate scope affording the products in good to excellent yields in a short time. Moreover, a ratio of S/C up to 10 000 could be achieved with gram scale synthesis.

## Abstract 2

An efficient method to construct tetracyclic isoquinolinium salts *via* C–H activation and [4 + 2] annulation reactions in ethanol is described.

## Graphical abstract

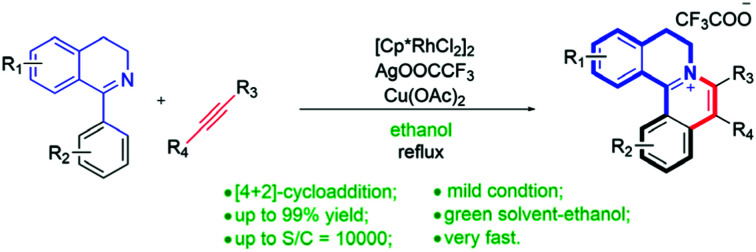

## Introduction

**Fig. 1:**
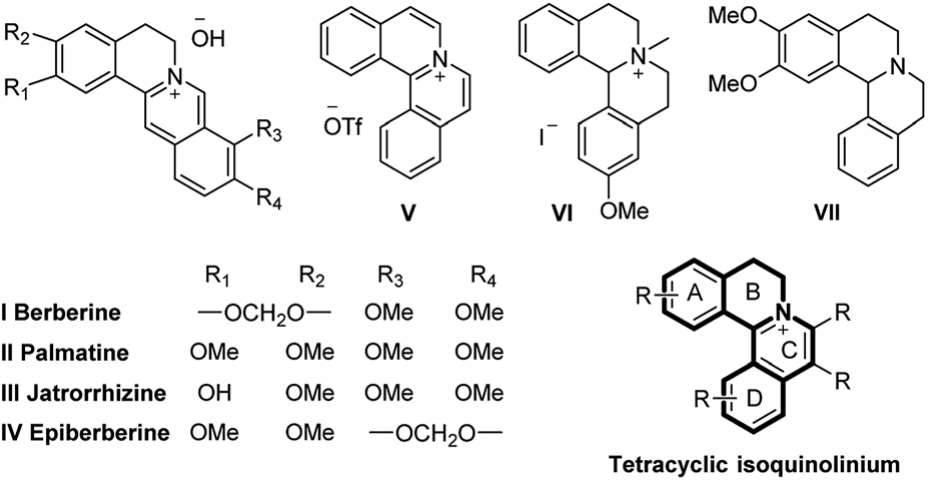
Representative tetracyclic isoquinolinium salts and their derivatives in medicinal chemistry and natural products.

N-Heterocyclic quaternary ammonium salts and their derivatives are versatile heterocyclic compounds found in many natural^1^ and synthetic products^2^ and are well-known for their potent biological activities^3^ (Fig. 1). Therefore, the development of improved methodologies to synthesize new N-heterocyclic quaternary ammonium salts still remains highly desirable.

**Scheme 1:**
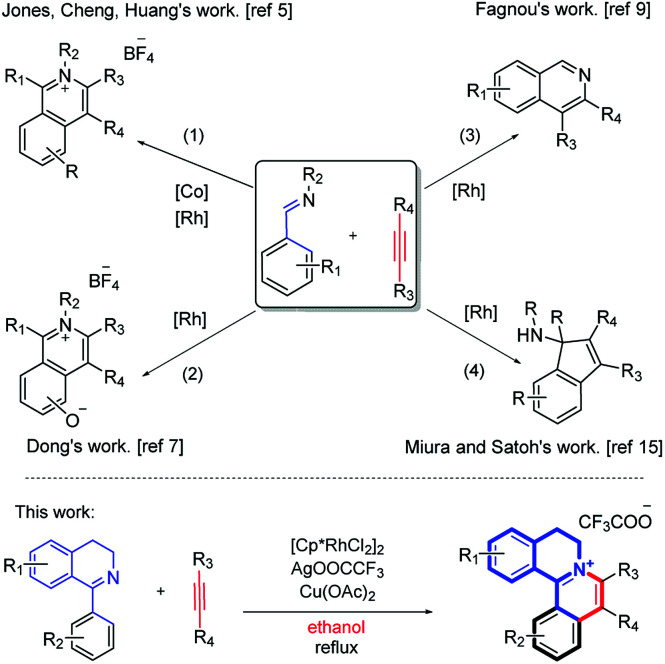
Imine-directed C–H activation.

In recent years, significant advancements have been made in transition-metal-catalyzed C–C bond formation *via* C–H activation, among which rhodium-catalyzed direct C–H bond activations are powerful strategies to synthesize various polycyclic skeletons and N-heterocyclic scaffolds, due to their high efficiency and atom economy.^4^ Particularly, using aldehyde imine/ketimine substrates to construct an isoquinoline skeleton *via* C–H annulation has been documented. Earlier, methods for the synthesis of isoquinolinium salts by C–H activation and [4 + 2] annulation of various imines have been studied (Scheme 1, eqn (1) and (2)),^5^ such as by Cheng's group^5*b*,6^ and Xu's group.^7^ Recently, You's group reported a Rh-catalyzed cascade C–H activation/[4 + 2] annulation of aldoximes with alkynes to synthesize multisubstituted protoberberine skeletons.^8^ In the meantime, the synthesis of isoquinoline compounds by the [4 + 2] annulation of open-ring imines has also been reported (Scheme 1, eqn (3)).^9^ Fagnou's group used [Cp*Rh(MeCN)_3_][SbF_6_]_2_ to catalyze the formation of isoquinoline compounds from *N-tert*-butylbenzaldimines and internal alkyne.^9^ Similar work has also been reported by Lade,^10^ Dong,^11^ Chiba,^12^ Cheng,^13^ Zhao^14^*et al.* In addition, there are some reports about the reaction of Rh, Ir and Ru-catalyzed [3 + 2] annulation using imine as a directing group (Scheme 1, eqn (4)).^15^

A close look at the literature precedents revealed that all the previously elegant examples regarding the isoquinolinium salts syntheses mainly focused on the use of acyclic aldimines or ketimines, while not even a single example, starting from the cyclic imines, such as 3,4-dihydroisoquinoline, has been documented. Inspired by these work, we proposed that it was possible to use the imine group of dihydroisoquinoline as a directing group to furnish C–H activation and [4 + 2] annulation to construct tetracylic isoquinolinium salts. Herein, we report Rh-catalyzed [4 + 2] annulations of cyclic-imine of 1-phenyl-3,4-dihydroisoquinolines to synthesis tetracyclic isoquinolinium salts in ethanol. Notably, ethanol is safer and more environmentally friendly compared with some other organic solvents, especially poisonous DCE. Noteworthily, this reaction proceeds with excellent efficiency. What's more, the ratio of S/C could achieve up to 10 000.

## Results and discussion

At the outset of our study, [Cp*RhCl_2_]_2_ was used to catalyze 6,7-dimethoxy-1-phenyl-3,4-dihydroisoquinoline 1a with diphenylacetylene 2a to investigate the catalytic performance of additives, solvents, and oxidants (Table 1). When [Cp*RhCl_2_]_2_ was used as a catalyst, without any additives, 3aa was barely formed in dioxane (entry 1). Then we explored various silver salts, among which AgOOCCF_3_ performed most remarkably, and provided 68% yield in 4 h (entry 3). Other silver salts showed little poor performance (entries 2, 4). Meanwhile, the effects of different solvents were investigated (entries 5–10). It is noteworthy that the reaction could get almost quantitative yield in ethanol, with 99% yield in 4 h (entry 10). Meanwhile, we compared the effects of additives, such as copper salts, K_2_S_2_O_8_, C_6_H_5_I(O_2_CCH_3_)_2_ and 2,3-dicyano-5,6-dichlorobenzoquinone (DDQ) (entries 11–16). Among these additives, Cu(OAc)_2_ gave the best yield of 99% for 3aa in 10 minutes (entry 12). However, the reaction could not proceed when only AgOOCCF_3_ and Cu(OAc)_2_ were used without Rh catalyst. (entry 17). What is noteworthy is that even when the S/C ratio was 1000, the reaction could finish completely in 10 minutes with 99% isolated yield (entry 18).

**Table 1:** Optimization of the reaction conditions^a^

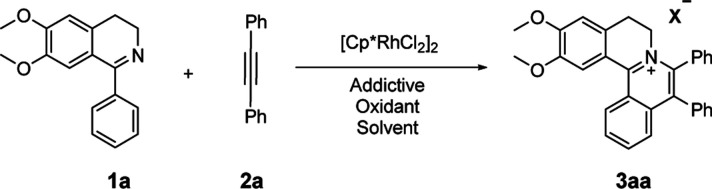					
Entry	Oxidant	Additive	Solvent	Time	3aa (%)^b^
1	—	—	Dioxane	4 h	ND
2	AgOTf	—	Dioxane	4 h	40
3	AgOOCCF_3_	—	Dioxane	4 h	68
4	AgOAc	—	Dioxane	4 h	Trace
5	AgOOCCF_3_	—	DCE	4 h	70
6	AgOOCCF_3_	—	Toluene	4 h	95
7	AgOOCCF_3_	—	DCM	4 h	25
8^c^	AgOOCCF_3_	—	DMF	4 h	70
9^c^	AgOOCCF_3_	—	DMSO	4 h	45
10	AgOOCCF_3_	—	EtOH	4 h	99
11^d^	AgOOCCF_3_	CuCl_2_·2H_2_O	EtOH	10 min	25
12^d^	AgOOCCF_3_	Cu(OAc)_2_	EtOH	10 min	99
13^d^	AgOOCCF_3_	Cu(CF_3_COO)_2_	EtOH	10 min	92
14^d^	AgOOCCF_3_	K_2_S_2_O_8_	EtOH	10 min	81
15^d^	AgOOCCF_3_	C_6_H_5_I(O_2_CCH_3_)_2_	EtOH	10 min	90
16^d^	AgOOCCF_3_	DDQ	EtOH	10 min	Trace
17^d^^,^^e^	AgOOCCF_3_	Cu(OAc)_2_	EtOH	10 min	ND
18^f^	AgOOCCF_3_	Cu(OAc)_2_	EtOH	10 min	99

a Reaction conditions unless otherwise specified: 1a (0.32 mmol), 2a (1 eq.), 0.5 mol% of [Cp*RhCl_2_]_2_, 1.0 eq. of oxidants, 3 mL of solvent, reflux, ND = Not Detected.

b Isolated yield.

c 120 °C.

d 1 eq. of additives.

e No [Cp*RhCl_2_]_2_ was added.

f S/C = 1000, 1a (1.6 mmol), 2a (1 eq.), 1.5 eq. of oxidants, 1 eq. of Cu(OAc)_2_.

**Fig. 2:**
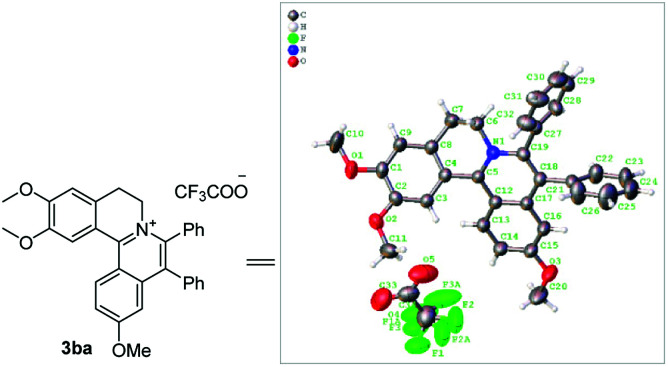
X-ray crystal structure of 3ba.

With the optimized conditions in hand, a range of electronically and sterically diverse of 3,4-dihydroisoquinoline derivatives were employed using 2a as a coupling partner to test the substrate tolerance of the Rh(iii)-catalyzed tandem [4 + 2] annulation. And the corresponding tetracyclic isoquinolinium salts were constructed in Table 2. A series of 1-aryl-substituted 3,4-dihydroisoquinoline (1a–g) could be effectively worked with 2a in the catalytic reaction with excellent yields (99–95%). However, when the ortho-position of benzene ring was substituted by methoxy group (1h), the reaction speed went down apparently. The product 3ia and 3ia′ was successfully obtained in 96% yield with the regioselectivity of 1 : 1. In addition, using naphthalene-substituted 1j, the reaction proceed slower than 1a, which indicated that steric effect could influence the reaction process. Then, we investigated the effect of steric and electronic influences on the isoquinoline core (1k–o). Notably, the substrates bearing both electron-withdrawing and electron-donating substituted at the *ortho*-, *meta*-, and *para*-positions of the phenyl ring (1b, 1k–o) reacted fast to provide excellent yields. However, the reaction significantly weakened to 65% yield even in 48 hours when 1q was used as a substrate. For naphthalene-substituted substrates, 1s gave mixture product 3sa and 3sa′. Interestingly, 1j only afforded pure product 3ja. It indicated that electronic influences on the isoquinoline core played an important role on the regioselectivity of the reaction process. Notably, the 1t could be converted to corresponding quaternary ammonium salt 3ta in good yield, whose reduction product bears the key hetero-tetracyclic scaffolds of reserpine.^16^ The structure of the final product 3ba was characterized by X-ray crystallography (Fig. 2).

**Table 2:** Substrate Scope of dihydroisoquinolines^a^^,^^b^

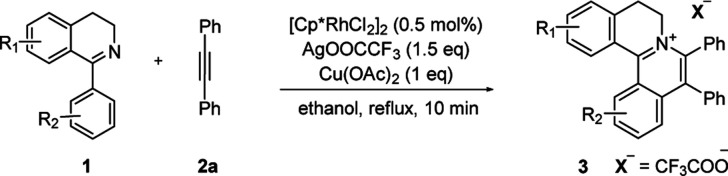
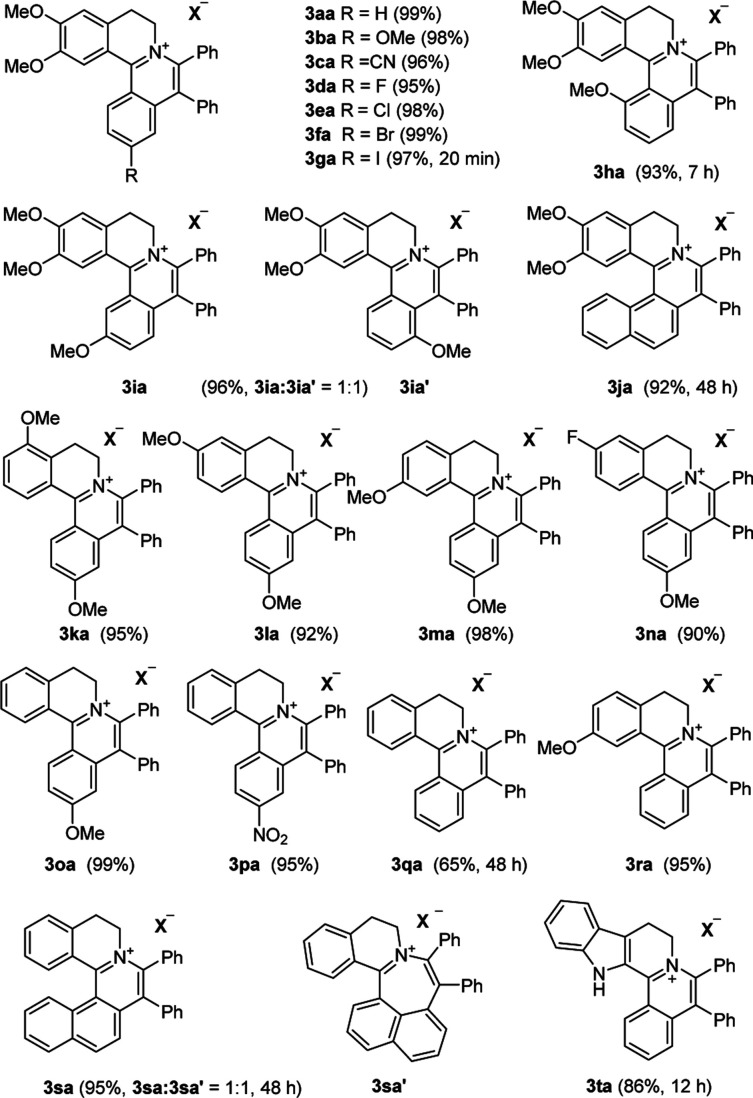

a Reaction conditions unless otherwise specified: 0.32 mmol of 1, 0.32 mmol of 2a, 0.5 mol% of [Cp*RhCl_2_]_2_, 1.5 eq. of AgOOCCF_3_, 1 eq. of Cu(OAc)_2_, 3 mL of ethanol, 10 min.

b Isolated yield.

The examination of the scope of various alkynes 2 was shown in Table 3. The result revealed that wide substrate tolerance with both internal aryl and alkyl alkynes. For alkynes, both electron-donating and -withdrawing substituents on the phenyl ring proceeded smoothly with 1a to furnish 3ab–e with good yields, though need a longer time than disubstituent alkynes 2a. To our delight, alkyl-substituted alkyne 2f exhibited the similar excellent reactivity as that of aryl-substituted alkyne. Especially, different with other's work,^5*c*,6*a*,6*c*,7,8^ unsymmetrical alkynes 2g and 2h reacted very fast with 1a to give pure products instead of regioisomers with excellent yields in 10 minutes. The structure of 3ag and 3ah were determined by the NOESY analysis. It needs to point out that the present conditions are specific and highly efficient for the synthesis of tetracylic isoquinolinium salts.

**Table 3:** Substrate Scope of the alkynes^a^^,^^b^

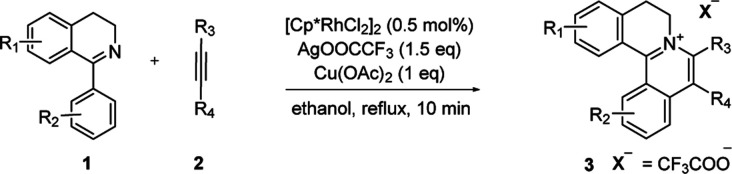
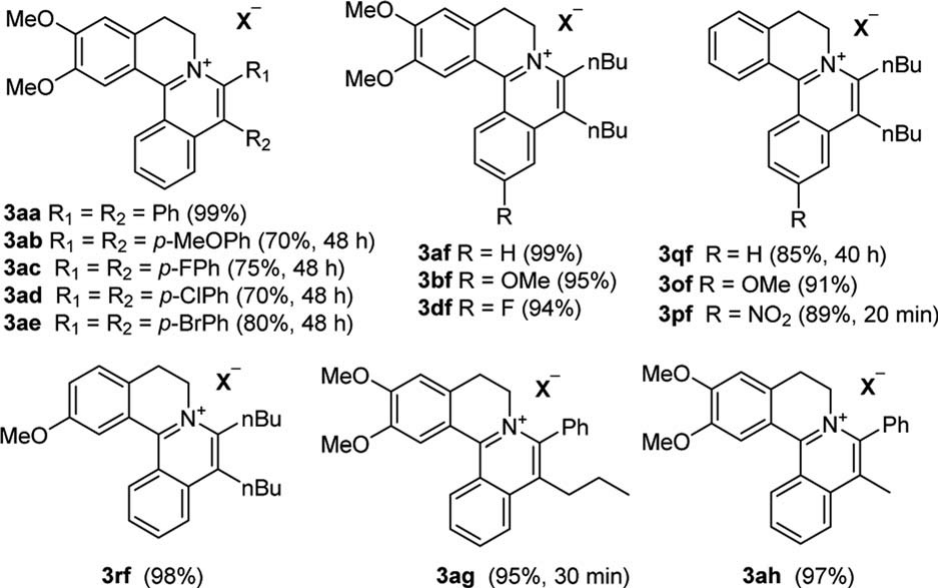

a Reaction conditions unless otherwise specified: 0.32 mmol of 1, 2 (1 eq.), 0.5 mol% of [Cp*RhCl_2_]_2_, 1.5 eq. of AgOOCCF_3_, 1 eq. of Cu(OAc)_2_, 3 mL of ethanol, 10 min.

b Isolated yield.

Considering, metal alkenyl intermediates may undergo [3 + 2] annulation to imine group for non-cyclicimines, we have also investigated the scope of non-cyclicimine substrates in Table S2.† Interestingly, we found non-cyclicimines gave N-heterocyclic quaternary ammonium salts of [4 + 2] annulation of imines not of [3 + 2] annulation. Generally, the results of Table 2 and S2† suggested that the dihydroisoquinoline showed much higher activity than non-cyclicimines in the [Cp*RhCl_2_]_2_/AgOOCCF_3_/Cu(OAc)_2_ catalyst system.

To assess the scalability of this Rh(iii)-catalyzed C–H bond activation and annulation process, gram-scale reaction of 1a with 2a and 2f were performed (Table 4). Firstly, 99% yield of 3aa was obtained in 10 minutes while the S/C = 1000 (entry 1). Then the S/C was gradually increased to 10 000, and 4.505 g of 3aa was obtained in 99% yield in 22 h (entry 4). What's more, the alkynes 2f could conduct well with 1a at 5000 of S/C ratio and 2.09 g of 3af was obtained in 99% yield (entry 5). These results showed the catalytic system has fairly good catalytic capability and practicality.

**Table 4:** Gram-scale synthesis of 3aa and 3afa^,^^b^

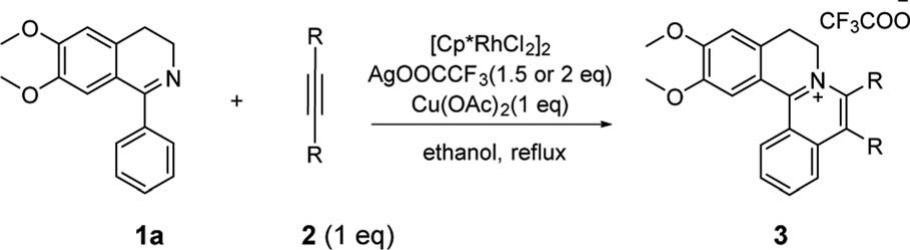				
Entry	S/C	3/g	Time	Yield%
1	1000	3aa/0.451	10 min	99
2^c^	5000	3aa/2.252	24 h	99
3^d^	5000	3aa/2.253	2.5 h	99
4^e^	10 000	3aa/4.505	22 h	99
5^f^	5000	3af/2.090	48 h	99

a Reaction conditions unless otherwise specified: 1a (0.8091 mmol), 1.5 eq. of AgOOCCF_3_, 15 mL of ethanol.

b Isolated yield.

c 1a (4.0453 mmol).

d 1a (4.0453 mmol), 2 eq. of AgOOCCF_3_.

e 1a (8.0906 mmol), 2 eq. of AgOOCCF_3_.

f 1a (4.0453 mmol), 2 eq. of AgOOCCF_3_, 15 mL of ethanol.

## Conclusions

In summary, we have developed a simple and efficient catalytic method for transforming the imine substrates especially the dihydroisoquinoline compounds to quaternary ammonium salts with the utilization of rhodium catalyzed C–H activation and [4 + 2] annulation in ethanol in a very short time and with remarkable yield under mild reaction conditions. Additionally, with the aid of AgOOCCF_3_ and Cu(OAc)_2_, the reaction time and catalytic performance can be greatly enhanced, so that a ratio of S/C up to 10 000 could be achieved with gram scale substrate. It provides an efficient strategy to synthesise tetracyclic isoquinolinium salts.

## Conflicts of interest

There are no conflicts to declare.

## Supplementary Material

RA-008-C8RA05443F-s001

RA-008-C8RA05443F-s002
