# Supplementary material for: Rh(iii)-catalyzed synthesis of tetracyclic isoquinolinium salts via C–H activation and [4+2] annulation of 1-phenyl-3,4-dihydroisoquinolines and alkynes in ethanol
Source: RSC Adv. 2018 Aug 24;8(52):30050–4. doi: 10.1039/c8ra05443f (PMC9085511; doi:10.1039/c8ra05443f)
Supplement: RA-008-C8RA05443F-s001 [file RA-008-C8RA05443F-s001.pdf]

Electronic Supplementary Material (ESI) for RSC Advances.
This journal is © The Royal Society of Chemistry 2018

Supporting information

Rh(III)-catalyzed Synthesis of Tetracyclic Isoquinolinium Salts via C-H Activation and [4+2] Annulation of 1-Phenyl-3,4-Dihydroisoquinolines and Alkynes in Ethanol

Xinxin Dang,[a] Yu He,[a] Yingtian Liu,[a] Xuehong Chen,[a] Jun-long Li,[b] Xian-li Zhou,*[a] Hezhong Jiang,*[, a] Jiahong Li.*[, a]

a School of Life Science and Engineering, Southwest Jiaotong University Chengdu 610041, China;

b Antibiotics Research and Re-evaluation Key Laboratory of Sichuan Province, Sichuan Industrial Institute of Antibiotics, Chengdu University Chengdu, 610052, China.

Table of contents

1. **General Methods** **S2**
2. **Optimization of the reaction conditions** **S2**
3. **General Procedure for Synthesis of Tetracyclic Isoquinolinium Salts and the Scope of Non-Cyclicimine Substrates** **S3**
4. **Characterization Data** **S4**
5. **Reference**..**S16**
6. **NMR Spectra** **S16**
7. **Crystal Data**...**S66**

1. General Methods

Unless otherwise noted, all reagents were obtained from commercial suppliers and used without further purification. [(Cp*RhCl$_2$)$_2$], AgOOCCF$_3$ and diphenylacetylene was purchased from aladdin. Symmetrical diarylalkynes **2b-e**[1] and 1-phenyl-3,4-dihydroisoquinolines[2] were prepared according to the literature procedures. Ethanol was obtained by atmospheric distillation.

NMR spectra were obtained on a JNM-ECZ600R/S1 spectrometer. NMR data were obtained for ^{1}H at 600 MHz, and for ^{13}C at 150 MHz. The ^{1}H NMR (600 MHz) chemical shifts Chemical shifts were measured relative to CDCl$_3$ or DMSO-d$_6$ or CD$_3$OD as the internal references (CDCl$_3$: δ = 7.26, DMSO-d$_6$: δ = 2.50, CD$_3$OD: δ = 3.31). The ^{13}C NMR (150 MHz) chemical shifts were given using CDCl$_3$ or DMSO-d$_6$ or CD$_3$OD as the internal references (CDCl$_3$: δ = 77.16, DMSO-d$_6$: δ = 39.52, CD$_3$OD: δ = 49.00). ESI HRMS was recorded on a Waters SYNAPT G2 and Water XEVO G2 Q-ToF. TLC was performed on glass-backed silica plates. Column chromatography was performed on silicagel (300-400 mesh), eluting with dichloromethane and methanol.

2. Optimization of the reaction conditions

Table S1. Screening for the optimal reaction conditions[a,b]

Entry	Catalyst	Oxidant	Addictive	Acid/base	T/ °C	Solvent	Time	**3aa** (%)
1	[Cp*RhCl$_2$]$_2$	-	-	-	Reflux	dioxane	4 h	Trace
2	[Cp*RhCl$_2$]$_2$	AgOTf			Reflux	dioxane	4 h	40
3	[Cp*RhCl$_2$]$_2$	Ag(OAc)$_2$			Reflux	dioxane	4 h	Trace
4	[Cp*RhCl$_2$]$_2$	AgSbF$_6$			Reflux	dioxane	4 h	34
6	[Cp*RhCl$_2$]$_2$	AgBF$_4$			Reflux	dioxane	4h	30
7	[Cp*RhCl$_2$]$_2$	AgOOCCF$_3$			Reflux	dioxane	4 h	68
8	-	AgOOCCF$_3$			Reflux	dioxane	4 h	ND
9[c]	[Cp*RhCl$_2$]$_2$	AgOOCCF$_3$		NaOH	Reflux	dioxane	4 h	24
10[d]	[Cp*RhCl$_2$]$_2$	AgOOCCF$_3$		Na$_2$CO$_3$	Reflux	dioxane	4 h	57
11	[Cp*RhCl$_2$]$_2$	AgOOCCF$_3$			Reflux	TFA	4 h	Trace
12	[Cp*RhCl$_2$]$_2$	AgOOCCF$_3$			Reflux	DCE	4 h	70
13	[Cp*RhCl$_2$]$_2$	AgOOCCF$_3$			Reflux	toluene	4 h	95
14	[Cp*RhCl$_2$]$_2$	AgOOCCF$_3$			Reflux	DCM	4 h	25
15	[Cp*RhCl$_2$]$_2$	AgOOCCF$_3$			120	decalin	4 h	Trace
16	[Cp*RhCl$_2$]$_2$	AgOOCCF$_3$			120	DMF	4 h	70
17	[Cp*RhCl$_2$]$_2$	AgOOCCF$_3$			120	DMSO	4 h	45
18	[Cp*RhCl$_2$]$_2$	AgOOCCF$_3$			Reflux	H_2O	4 h	30

19[e]	$[Cp^*RhCl_2]_2$	$AgOOCCF_3$			Reflux	H_2O+CTAB	4 h	52
20	$[Cp^*RhCl_2]_2$	$AgOOCCF_3$			Reflux	EtOH	4 h	99
21	$[Cp^*RhCl_2]_2$	$AgOOCCF_3$			Reflux	EtOH	1 h	69
22	$[Cp^*RhCl_2]_2$	$AgOOCCF_3$			Reflux	isopropanol	1 h	65
23	$[Cp^*RhCl_2]_2$	$AgOOCCF_3$			120	butyl alcohol	1 h	67
24	$[Cp^*RhCl_2]_2$	$AgOOCCF_3$			Reflux	TFEA	1 h	67
25	$[Cp^*RhCl_2]_2$	$AgOOCCF_3$	air		Reflux	EtOH	10min	73
26	$[Cp^*RhCl_2]_2$	$AgOOCCF_3$	$K_2S_2O_8$		Reflux	EtOH	10 min	81
27	$[Cp^*RhCl_2]_2$	$AgOOCCF_3$	$C_6H_5I(O_2CCH_3)_2$		Reflux	EtOH	10 min	90
28	$[Cp^*RhCl_2]_2$	$AgOOCCF_3$	DDQ		Reflux	EtOH	10 min	Trace
29	$[Cp^*RhCl_2]_2$	$AgOOCCF_3$	$CuSO_4$		Reflux	EtOH	1 h	74
30	$[Cp^*RhCl_2]_2$	$AgOOCCF_3$	$CuCl_2.2H_2O$		Reflux	EtOH	1 h	65
31	$[Cp^*RhCl_2]_2$	$AgOOCCF_3$	$Cu(NO)_3.2H_2O$		Reflux	EtOH	1 h	66
32	$[Cp^*RhCl_2]_2$	$AgOOCCF_3$	$Cu(OOCCF_3)_2.H_2O$		Reflux	EtOH	10 min	95
33[f]	$[Cp^*RhCl_2]_2$	$AgOOCCF_3$	$Cu(OOCCF_3)_2.H_2O$		Reflux	EtOH	10 min	75
34	$[Cp^*RhCl_2]_2$	$AgOOCCF_3$	$Cu(OAc)_2$		Reflux	EtOH	10 min	99
35[f]	$[Cp^*RhCl_2]_2$	$AgOOCCF_3$	$Cu(OAc)_2$		Reflux	EtOH	10 min	99
36[g]	$[Cp^*RhCl_2]_2$	$Cu(OAc)_2$	$AgOOCCF_3$		Reflux	EtOH	10 min	99
37[h]	$[Cp^*RhCl_2]_2$	$Cu(OAc)_2$	$AgOOCCF_3$		Reflux	EtOH	10 min	99
38[i]	$[Cp^*RhCl_2]_2$	$Cu(OAc)_2$	$AgOOCCF_3$		Reflux	EtOH	10 min	85
39[j]	$[Cp^*RhCl_2]_2$	$Cu(OAc)_2$	$AgOOCCF_3$		Reflux	EtOH	10 min	81
40	$[Cp^*RhCl_2]_2$	$AgOOCCF_3$	air		Reflux	EtOH	10min	73
41	-	-	$Cu(OAc)_2$		Reflux	EtOH	10 min	ND
42	-	$AgOOCCF_3$	$Cu(OAc)_2$		Reflux	EtOH	10 min	ND
43	$[Cp^*RhCl_2]_2$	$Cu(OAc)_2$	-		Reflux	EtOH	10 min	85
44	$[RuCl_2cyme]_2$	$AgOOCCF_3$	$Cu(OAc)_2$		Reflux	EtOH	10 min	Trace
45	$[Cp^*IrCl_2]_2$	$AgOOCCF_3$	$Cu(OAc)_2$		Reflux	EtOH	10 min	Trace

[a]Reaction conditions: **1a** (0.32 mmol), **2a** (0.32 mmol), 0.5 mol% of $[Cp^*RhCl_2]_2$, 1 eq of additive, 1 eq of oxidants, 3 mL of solvent, ND = Not Detected; [b]isolated yields; [c]NaOH (20 mol%); [d]Na_2CO_3 (20 mol%); [e]CTAB (Cetyltrimethyl Ammonium Bromide) (5 mol%); [f]S/C = 400; [g]1.0 eq of $AgOOCCF_3$, [h]0.5 eq $AgOOCCF_3$. [i]0.1 eq $AgOOCCF_3$. [j]0.05eq $AgOOCCF_3$.

3. General Procedure for Synthesis of Tetracyclic Isoquinolinium Salts and the Scope of Non-Cyclicimine Substrates

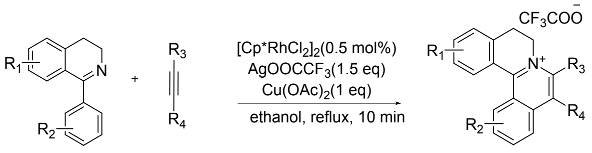

1 **2** **3**

General procedure: A reaction tube with a magnetic stir bar was charged with **1** (0.32 mmol, 1 eq), **2** (0.32 mmol, 1 eq), $[Cp^*RhCl_2]_2$ (0.5 mol%), $AgOOCCF_3$ (1.5 eq) and $Cu(OAc)_2$ (1 eq) and ethanol (3 mL), reflux. The resulting mixture was stirred for 10 min. After completion, the reaction mixture was purified by flash chromatography eluting with methanol and dichloromethane (1:20) to give the product.

Table S2. Scope of Non-Cyclicimine Substrates

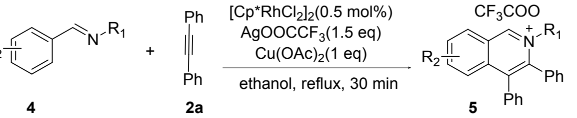

4 **2a** **5**

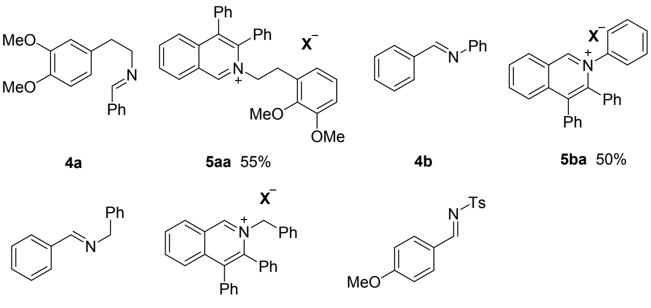

4a **5aa** 55% **4b** **5ba** 50%

4c **5ca** 70% **4d** Decomposed

General procedure: A reaction tube with a magnetic stir bar was charged with **4** (0.32 mmol, 1 eq), 1, 2-diphenylethyne **2a** (0.32 mmol, 1 eq), $[Cp^*RhCl_2]_2$ (0.5 mol%), $AgOOCCF_3$ (1.5 eq) and $Cu(OAc)_2$ (1 eq) and ethanol (3 mL). The resulting mixture was stirred for 30 min. After completion, the reaction mixture was purified by flash chromatography eluting with methanol and dichloromethane (1:20) to give the product.

4. Characterization Data

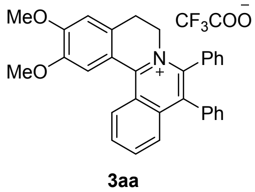

3aa

2,3-dimethoxy-8,9-diphenyl-5,6-dihydroisoquinolino[1,2-a]isoquinolin-7-ium 2,2,2-trifluoroacetate (3aa). A yellow solid (176.9 mg, 99% yield). ^{1}H NMR (600 MHz, $CDCl_3$): δ 8.68 (d, J = 8.89 Hz, 1H), 7.94 (t, J = 7.38 Hz, 1H), 7.89 (t, J = 7.68 Hz, 1H), 7.72 (d, J = 8.52 Hz, 1H), 7.50 (s, 1H), 7.45 (m, 2H), 7.35 (m, 3H), 7.30 (m, 3H), 7.25 (m, 2H), 7.08 (s, 1H), 4.44 (t, J = 5.25, 2H), 4.05 (s, 3H), 3.96 (s, 3H), 3.25 (t, J = 5.25, 2H) ppm; ^{13}C NMR (150 MHz, $CDCl_3$): δ 154.6, 154.1, 148.5, 144.3, 139.0, 136.1, 135.5, 133.8, 136.6, 131.6, 130.7, 130.5, 130.4, 130.3, 130.2, 129.1, 128.6, 128.5, 127.3, 125.1, 119.0, 116.2, 110.9, 56.8, 56.7, 52.4, 27.3 ppm. ESI HRMS: calcd. for $C_{31}H_{26}NO_2^+$ $[M-CF_3COO]^+$ 444.19634, found 444.1989.

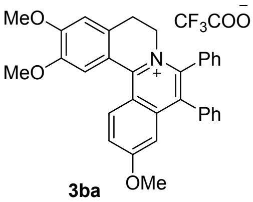

3ba **2,3,11-trimethoxy-8,9-diphenyl-5,6-dihydroisoquinolino[1,2-a]isoquinolin-7-ium 2,2,2-trifluoroacetate (3ba)**. A yellow solid (186.5 mg, 98% yield). ^{1}H NMR (600 MHz, $CDCl_3$): δ 8.60 (d, J = 8.63 Hz, 1H), 7.51 (d, J = 8.03 Hz, 1H), 7.46 (s, 1H), 7.34 (s, 5H), 7.29 (m, 3H), 7.18 (m, 2H), 7.09 (s, 1H), 6.89 (s, 1H), 4.30 (s, 2H), 4.04 (s, 3H), 3.97 (s, 3H), 3.82 (s, 3H), 3.21 (s, 2H) ppm; ^{13}C NMR (150 MHz, $CDCl_3$): δ 165.3, 154.2, 152.6, 148.4, 144.4, 141.9, 134.5, 134.0, 133.4, 132.8, 131.7, 130.4, 130.3, 130.2, 129.1, 128.7, 128.6, 122.7, 120.2, 119.0, 115.9, 110.9, 106.0, 56.9, 56.7, 56.2, 51.9, 27.5 ppm. ESI HRMS: calcd. for $C_{32}H_{28}NO_3^+$ [M-CF_3COO]$^+$ 474.20690, found 474.2079.

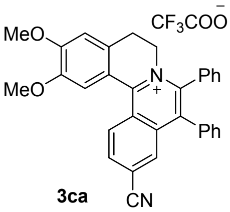

3ca **11-cyano-2,3-dimethoxy-8,9-diphenyl-5,6-dihydroisoquinolino[1,2-a]isoquinolin-7-ium 2,2,2-trifluoroacetate (3ca)**. A yellow solid (179.5 mg, 96% yield). ^{1}H NMR (600 MHz, $CDCl_3$): δ 8.89 (s, 1H), 8.66 (d, J = 9.36 Hz, 1H), 8.50 (d, J = 9.36 Hz, 1H), 8.15 (s, 1H), 7.51 (s, 1H), 7.36-7.35 (m, 3H), 7.30-7.27 (m, 4H), 7.17-7.15 (m, 2H), 6.98 (s, 1H), 4.29 (s, 2H), 4.29 (s, 3H), 4.03 (s, 3H), 3.14 (s, 2H) ppm; ^{13}C NMR (150 MHz, $CDCl_3$): δ 168.0, 155.0, 153.8, 149.2, 144.1, 138.8, 132.6, 131.7, 131.0, 130.5, 130.3, 130.2, 129.4, 129.0, 128.8, 127.0, 126.1, 119.0, 116.2, 110.6, 57.0, 56.8, 52.3, 27.4 ppm. ESI HRMS: calcd. for $C_{32}H_{25}N_2O_2^+$ [M-CF_3COO]$^+$ 469.19159, found 469.1938.

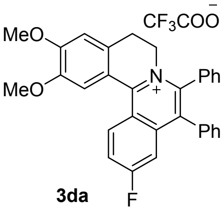

3da **11-fluoro-2,3-dimethoxy-8,9-diphenyl-5,6-dihydroisoquinolino[1,2-a]isoquinolin-7-ium 2,2,2-trifluoroacetate (3da)**. A yellow solid (176.5 mg, 95% yield). ^{1}H NMR (600 MHz, $CDCl_3$): δ 8.73 (q, 1H), 7.63 (t, J = 8.44 Hz, 1H), 7.46 (s, 1H), 7.44 (d, J = 3.78, 2H), 7.35-7.34 (m, 3H), 7.32-7.28 (m, 4H), 7.20 (m, 2H), 7.05 (s, 1H), 4.40 (s, 2H), 4.05 (s, 3H), 3.98 (s, 3H), 3.23 (s, 2H) ppm; ^{13}C NMR (150 MHz, $CDCl_3$): δ 166.4 (d, J = 261.1 Hz), 154.6, 154.0, 148.6, 145.1, 141.7 (d, J = 10.5 Hz), 135.3, 135.4, 134.6, 134.5, 133.5 (d, J = 11.5 Hz), 131.5, 130.4, 130.3 (d, J = 4.3 Hz), 129.2, 128.8, 128.7, 122.4, 120.7 (d, J = 24.3 Hz), 118.9, 116.2, 111.6 (d, J = 23.1 Hz), 110.8, 56.9, 56.7, 52.2, 27.3 ppm. ESI HRMS: calcd. for $C_{31}H_{25}FNO_2^+$ [M-CF_3COO]$^+$ 462.18691, found 462.1881.

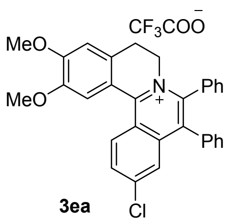

3ea **11-chloro-2,3-dimethoxy-8,9-diphenyl-5,6-dihydroisoquinolino[1,2-a]isoquinolin-7-ium 2,2,2-trifluoroacetate (3ea)**. A yellow solid (191.7 mg, 98% yield). ^{1}H NMR (600 MHz, $CDCl_3$): δ 8.64 (s, 1H), 7.82 (s, 1H), 7.65 (s, 1H), 7.51-7.31 (m, 10H), 7.19 (s, 2H), 7.09 (s, 1H), 4.43 (s, 2H), 4.05 (s, 3H), 3.98 (s, 3H), 3.27 (s, 2H) ppm; ^{13}C NMR (150 MHz, $CDCl_3$): δ 154.8, 154.3, 148.7, 145.5, 143.0, 140.0, 135.1, 133.7, 133.3, 132.5, 131.5, 131.2, 130.6, 130.5, 130.4, 129.2, 128.8, 128.7, 126.1, 123.7, 118.9, 116.1, 110.9, 56.9, 56.8, 52.4, 27.3 ppm. ESI HRMS: calcd. for $C_{31}H_{25}ClNO_2^+$ [M-CF_3COO]$^+$ 478.15737, found 478.1592.

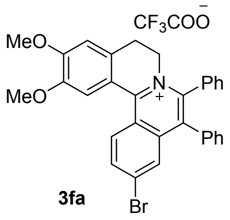

3fa **11-bromo-2,3-dimethoxy-8,9-diphenyl-5,6-dihydroisoquinolino[1,2-a]isoquinolin-7-ium 2,2,2-trifluoroacetate (3fa)**. A yellow solid (203.2 mg, 99% yield). ^{1}H NMR (600 MHz, $CDCl_3$): δ 8.54 (d, J = 8.50 Hz, 1H), 7.97 (d, J = 7.82 Hz, 1H), 7.86 (s, 1H), 7.47-7.42 (m, 3H), 7.35-7.31 (m, 6H), 7.19 (m, 2H), 7.07 (s, 1H), 6.89 (s, 1H), 4.41 (s, 2H), 4.06 (s, 3H), 3.98 (s, 3H), 3.20 (s, 2H) ppm; ^{13}C NMR (150 MHz, $CDCl_3$): δ 154.7, 154.4, 148.7, 145.6, 140.0, 135.0, 133.9, 133.6, 133.3, 132.2, 130.6, 130.6, 130.4, 129.5, 129.2, 128.9, 128.7, 123.9, 118.9, 116.1, 110.9, 56.9, 56.8, 52.5, 27.3 ppm. ESI HRMS: calcd. for $C_{31}H_{25}BrNO_2^+$ [M-CF_3COO]$^+$ 522.10690, found 522.1081.

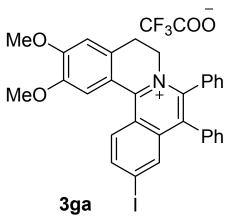

3ga **11-iodo-2,3-dimethoxy-8,9-diphenyl-5,6-dihydroisoquinolino[1,2-a]isoquinolin-7-ium 2,2,2-trifluoroacetate (3ga)**. A yellow solid (210.9 mg, 97% yield). ^{1}H NMR (600 MHz, $CDCl_3$): δ 8.32 (d, J = 8.91Hz, 1H), 8.13 (d, J = 9.06, 1.41 Hz, 1H), 8.05 (d, 1H), 7.40-7.37 (m, 3H), 7.34-7.30 (m, 6H), 7.18 (m, 2H), 7.06 (s, 1H), 4.36 (t, J = 6.03, 2H), 4.04 (s, 3H), 3.96 (s, 3H), 3.21 (t, J = 6.17, 2H) ppm; ^{13}C NMR (150 MHz, $CDCl_3$): δ 154.8, 154.5, 148.6, 145.6, 139.5, 139.2, 136.0, 134.5, 133.8, 133.3, 131.4, 130.5, 130.3, 129.2, 128.8, 128.7, 124.1, 118.8, 116.0, 110.9, 105.4, 56.8, 56.8, 52.4, 27.2 ppm. ESI HRMS: calcd. for $C_{31}H_{25}INO_2^+$ [M-CF_3COO]$^+$ 570.09317, found 570.0945.

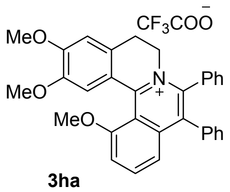

3ha

1,11,12-trimethoxy-5,6-diphenylisoquinolino[1,2-a]isoquinolin-7-ium 2,2,2-trifluoroacetate (3ha). A yellow solid (176.5 mg, 93% yield). ^{1}H NMR (600 MHz, CD_3OD): δ 7.97 (t, *J* = 7.80Hz, 1H), 7.47 (d, *J* = 9.60 Hz, 1H), 7.34 (m, 4H), 7.28 (m, 4H), 7.18-7.17 (m, 3H), 7.07-7.06 (m, 2H), 3.96 (s, 3H), 3.87 (s, 3H), 3.80 (s, 3H), 3.27 (m, 4H) ppm. ^{13}C NMR (150 MHz, CD_3OD) : δ 159.8, 155.7, 154.8, 149.3, 145.3, 141.7, 138.4, 136.6, 135.9, 133.4, 132.5, 131.0, 129.9, 129.5, 123.4, 119.7, 118.3, 118.0, 111.9, 110.2, 57.1, 56.8, 56.5, 53.2, 49.0, 27.5 ppm. ESI HRMS: calcd. for $C_{32}H_{28}NO_3^+$ [M-CF_3COO]$^+$ 474.20690, found 474.2079.

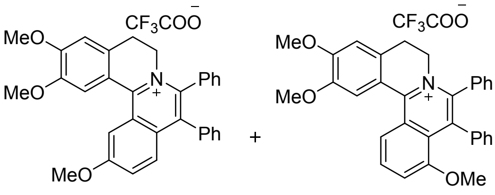

3ia 3ia' **2,3,12-trimethoxy-8,9-diphenyl-5,6-dihydroisoquinolino[1,2-a]isoquinolin-7-ium 2,2,2-trifluoroacetate (3ia)**. A yellow solid (182.5 mg, 96% yield). ^{1}H NMR (600 MHz, CD_3Cl_3): δ: 8.24 (d, *J* = 8.95Hz, 1H), 7.96 (s, 1H), 7.83 (t, *J* = 8.33Hz, 1H), 7.63 (d, *J* = 9.10Hz, 1H), 7.58-7.56 (m, 2H), 7.46 (s, 1H), 7.34-7.26 (m, 14H), 7.16-7.13 (m, 9H), 4.38-4.30 (m, 4H), 4.05 (s, 3H), 4.04 (s, 3H), 3.97 (s, 3H), 3.96 (s, 3H), 3.94 (s, 3H), 3.44 (s, 3H), 3.21 (s, 4H) ppm. ^{13}C NMR (150 MHz, CD_3OD): δ: 160.8, 156.7, 153.6, 148.3, 144.6, 138.2, 136.1, 134.7, 134.0, 131.7, 131.2, 130.5, 130.2, 129.9, 129.3, 129.2, 129.0, 128.9, 128.6, 128.6, 128.2, 127.1, 127.0, 126.9, 122.6, 119.3, 115.4, 111.0, 110.7, 108.3, 56.8, 56.8, 56.3, 56.1, 52.7, 52.6, 27.4, 27.2 ppm. ESI HRMS: calcd. for $C_{32}H_{28}NO_3^+$ [M-CF_3COO]$^+$ 474.20690, found 474.2079

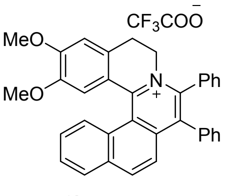

3ja **13,14-dimethoxy-4,5-diphenyl-1,2-dihydrobenzo[h]isoquinolino[1,2-a]isoquinolin-3-ium 2,2,2-trifluoroacetate (3ja)**. A yellow solid (178.8 mg, 92% yield). ^{1}H NMR (600 MHz, $CDCl_3$): δ 8.18 (d, *J* = 8.47 Hz, 1H), 8.11 (d, *J* = 9.42 Hz, 1H), 7.95 (d, *J* = 8.16 Hz, 1H), 7.68-7.63 (m, 2H), 7.45-7.40 (m, 3H), 7.37-7.28 (m, 6H), 7.23 (m, 1H), 7.16 (d, *J* = 6.8 Hz, 1H), 7.09 (s, 1H), 6.92 (s, 1H), 4.66 (d, *J* = 14.19 Hz, 1H), 4.23 (t, *J* = 15.12 Hz, 1H), 4.06 (s, 3H), 3.64 (t, *J* = 15.48 Hz, 1H), 3.39 (s, 3H), 3.21 (d, *J* = 15.37 Hz, 1H) ppm; ^{13}C NMR (150 MHz, $CDCl_3$): δ 154.4, 151.3, 148.5, 147.0, 141.0, 137.7, 135.8, 134.0, 133.7, 131.6, 131.3, 131.2, 130.8, 130.7, 130.3, 129.6, 129.5, 129.3, 129.2, 129.2, 129.0, 128.8, 128.7, 128.5, 127.3, 124.6, 122.8, 122.0, 115.6, 110.5, 56.7, 56.0, 52.6, 27.3 ppm. ESI HRMS: calcd. for $C_{35}H_{28}NO_2^+$ [M-CF_3COO]$^+$ 494.21199, found 494.2135.

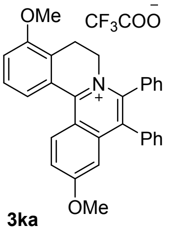

3ka

4,11-dimethoxy-8,9-diphenyl-5,6-dihydroisoquinolino[1,2-a]isoquinolin-7-ium 2,2,2-trifluoroacetate (3ka). A yellow solid (171.2 mg, 95% yield). ^{1}H NMR (600 MHz, $CDCl_3$): δ 8.58 (d, J = 8.76Hz, 1H), 7.59 (s,2H), 7.51 (d, J = 8.56, 1H), 7.43-7.26 (m, 9H), 7.21-7.20 (m, 2H), 6.90 (s, 1H), 4.28 (s, 2H), 3.94 (s, 3H), 3.81 (s, 3H), 3.18 (s, 2H) ppm; ^{13}C NMR (150 MHz, $CDCl_3$): δ 165.5, 155.9, 152.6, 144.7, 142.0, 135.5, 134.0, 133.2, 131.8, 130.3, 130.2, 129.1, 128.7, 128.6, 128.5, 127.9, 127.0, 125.4, 123.2, 120.8, 115.5, 105.7, 56.2, 56.1, 52.1, 20.9 ppm. ESI HRMS: calcd. for $C_{31}H_{26}NO_2^+$ [M-CF_3COO]$^+$ 444.19634, found 444.1989.

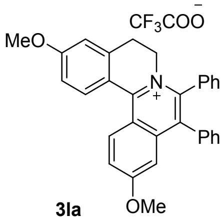

3la **3,11-dimethoxy-8,9-diphenyl-5,6-dihydroisoquinolino[1,2-a]isoquinolin-7-ium 2,2,2-trifluoroacetate (3la)**. A yellow solid (165.5 mg, 92% yield). ^{1}H NMR (600 MHz, $CDCl_3$): δ 8.57 (s, 1H), 7.98 (s, 1H), 7.52 (s, 1H), 7.43-7.26 (m, 8H), 7.26-7.08 (m, 4H), 6.89 (s, 1H), 4.37 (s, 2H), 3.97 (s, 3H), 3.81 (s, 3H), 3.34 (s, 2H) ppm; ^{13}C NMR (150 MHz, $CDCl_3$): δ 165.4, 164.3, 152.9, 144.4, 141.8, 141.3, 135.3, 134.3, 134.1, 133.3, 131.9, 130.4, 130.2, 130.2, 129.2, 128.7, 128.6, 122.8, 120.3, 119.4, 114.2, 113.5, 106.0, 56.2, 51.8, 28.2 ppm. ESI HRMS: calcd. for $C_{31}H_{26}NO_2^+$ [M-CF_3COO]$^+$ 444.19634, found 444.1989.

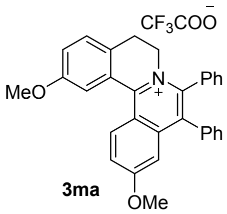

3ma **2,11-dimethoxy-8,9-diphenyl-5,6-dihydroisoquinolino[1,2-a]isoquinolin-7-ium 2,2,2-trifluoroacetate (3ma)**. A yellow solid (176.2 mg, 98% yield). ^{1}H NMR (600 MHz, $CDCl_3$): δ 8.61 (d, J = 8.46Hz, 1H), 7.51-7.48 (m, 2H), 7.38 (m, 2H), 7.35-7.27 (m, 6H), 7.23-7.20 (m, 3H), 6.90 (d, J = 2.40Hz, 1H), 4.37 (s, 2H), 4.31 (s, 2H), 3.92 (s, 3H), 3.82 (s, 3H), 3.15 (s, 2H) ppm; ^{13}C NMR (150 MHz, $CDCl_3$): δ 165.5, 158.8, 152.5, 144.9, 142.1, 135.5, 134.0, 132.8, 131.8, 130.4, 130.3, 130.3, 130.2, 129.2, 128.7, 128.7, 123.4, 120.6,119.1, 119.1, 105.8, 56.2, 56.0, 52.8, 27.1 ppm. ESI HRMS: calcd. for $C_{31}H_{26}NO_2^+$ [M-CF_3COO]$^+$ 444.19634, found 444.1989.

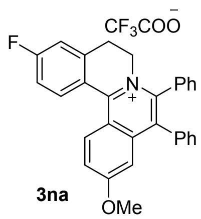

3na **3-fluoro-11-methoxy-8,9-diphenyl-5,6-dihydroisoquinolino[1,2-a]isoquinolin-7-ium 2,2,2-trifluoroacetate (3na)**. A yellow solid (158.3 mg, 90% yield). ^{1}H NMR (600 MHz, $CDCl_3$): 8.56 (d, J = 9.60, 1H), 7.98 (t, J = 7.52, 1H), 7.68 (t, J = 7.51, 1H), 7.61 (t, J = 7.38, 1H), 7.53-7.49 (m, 2H), 7.41 (m, 2H), 7.33-7.27 (m, 6H), 7.23-7.21 (t, 2H), 6.90 (d, J = 2.76, 1H), 4.34 (s, 2H), 3.82 (s, 3H), 3.27 (s, 2H) ppm; ^{13}C NMR (150 MHz, $CDCl_3$): δ 165.6 (d, J = 256.5 Hz), 165.4, 151.8, 144.9, 142.1, 142.0 (d, J = 9.2 Hz), 135.8 (d, J = 10.1 Hz), 135.4, 134.1, 132.7, 131.9, 130.4 (d, J = 4.5 Hz), 130.2, 129.1, 128.7, 128.6, 123.4, 120.6, 115.5 (d, J =

23.0 Hz), 115.3 (d, J = 21.4 Hz) 105.8, 56.2, 51.9, 20.0 ppm. ESI HRMS: calcd. for $C_{30}H_{23}FNO^+$ [M-CF_3COO]$^+$ 432.17635, found 432.1788.

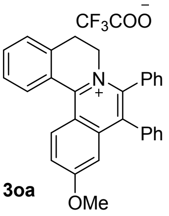

3oa

11-methoxy-8,9-diphenyl-5,6-dihydroisoquinolino[1,2-a]isoquinolin-7-ium 2,2,2-trifluoroacetate (3oa). A yellow solid (167.6 mg, 99% yield). ^{1}H NMR (600 MHz, $CDCl_3$): 8.56 (d, J = 9.60, 1H), 7.98 (t, J = 7.52, 1H), 7.68 (t, J = 7.51, 1H), 7.61 (t, J = 7.38, 1H), 7.53-7.49 (m, 2H), 7.41 (m, 2H), 7.33-7.27 (m, 6H), 7.23-7.21 (t, 2H), 6.90 (d, J = 2.76, 1H), 4.34 (s, 2H), 3.82 (s, 3H), 3.27 (s, 2H) ppm; ^{13}C NMR (150 MHz, $CDCl_3$): δ 165.5, 152.6, 144.9, 142.1, 138.8, 135.5, 134.0, 133.8, 133.2, 133.0, 131.8, 130.4, 130.2, 129.1, 128.7, 128.7, 128.2, 127.8, 126.9, 123.3, 120.7, 105.8, 56.2, 52.2, 28.0 ppm. ESI HRMS: calcd. for $C_{30}H_{24}NO^+$ [M-CF_3COO]$^+$ 414.18577, found 414.1873.

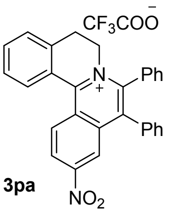

3pa

11-nitro-8,9-diphenyl-5,6-dihydroisoquinolino[1,2-a]isoquinolin-7-ium 2,2,2-trifluoroacetate (3pa). A yellow solid (166.5 mg, 95% yield). ^{1}H NMR (600 MHz, $CDCl_3$): δ 8.84 (d, J = 10.56, 1H), 8.55-8.53 (m, 2H), 8.02 (d, J = 7.81, 1H), 7.74 (t, J = 7.62, 1H), 7.64 (t, J = 7.71, 1H), 7.54-7.50 (m, 3H), 7.36-7.34 (m, 6H), 7.27-7.26 (m, 2H), 4.52 (t, J = 6.25, 2H), 3.31 (s, 2H) ppm; ^{13}C NMR (150 MHz, $CDCl_3$): δ 155.1, 150.9, 146.8, 139.5, 138.9, 138.8, 134.7, 133.8, 133.5, 132.8, 131.2, 130.6, 130.5, 130.4, 129.3, 129.2, 128.9, 128.2, 128.1, 127.9, 126.8, 123.3, 122.7, 53.4, 27.4 ppm. ESI HRMS: calcd. for $C_{29}H_{21}N_2O_2^+$ [M-CF_3COO]$^+$ 429.16029, found 429.1621.

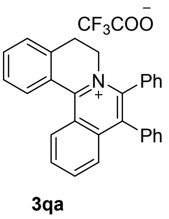

3qa

8,9-diphenyl-5,6-dihydroisoquinolino[1,2-a]isoquinolin-7-ium 2,2,2-trifluoroacetate (3qa). A yellow solid (104.2 mg, 65% yield). ^{1}H NMR (600 MHz, DMSO-d_6): δ 8.12 (t, J = 7.86Hz, 1H), 7.59-7.57 (m, 1H), 7.54-7.51 (m, 2H), 7.47-7.40 (m, 7H), 7.38-7.36 (m, 1H), 7.33-7.30 (m, 1H), 7.26 (t, J = 7.86Hz, 1H), 7.23 (d, J = 7.8Hz, 1H), 7.18-7.17 (m, 2H), 6.78-6.77 (m, 2H), 4.32 (t, J = 7.86Hz, 1H), 3.09 (t, J = 7.86Hz, 1H) ppm; ^{13}C NMR (150 MHz, DMSO-d6): δ 156.2, 150.0, 141.7, 138.0, 137.2, 135.9, 134.2, 130.3, 130.0, 129.8, 129.6, 129.3, 128.8, 128.7, 128.3, 128.1, 127.9, 126.6, 122.5, 121.3, 120.0, 50.9, 32.8 ppm. ESI HRMS: calcd. for $C_{29}H_{22}N^+$ [M-CF_3COO]$^+$ 384.17521, found 384.1764.

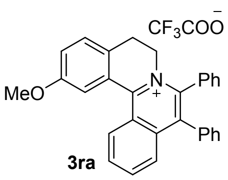

3ra **2-methoxy-8,9-diphenyl-5,6-dihydroisoquinolino[1,2-a]isoquinolin-7-ium 2,2,2-trifluoroacetate (3ra)**. A yellow solid (161.5 mg, 95% yield). ^{1}H NMR (600 MHz, $CDCl_3$): δ 8.73 (d, J = 8.26Hz, 1H), 7.98 (t, J = 7.86, 1H), 7.92 (t, J = 7.86, 1H), 7.76(d, J = 7.86, 1H), 7.56 (s, 1H), 7.46 (m, 3H), 7.34 (s, 3H), 7.22 (m, 3H), 4.45 (s, 2H), 3.92 (s, 3H), 3.21 (s, 2H) ppm; ^{13}C NMR (150 MHz, $CDCl_3$): δ 158.8, 154.1, 144.8, 139.1, 137.5, 135.8, 133.8, 131.6, 130.7, 130.5, 130.5, 130.3, 129.2, 129.2, 128.7, 128.5, 127.6, 127.3, 125.5, 119.5, 119.4, 56.1, 53.5, 27.0 ppm. ESI HRMS: calcd. for $C_{30}H_{24}NO^+$ $[M-CF_3COO]^+$ 414.18577, found 414.1872.

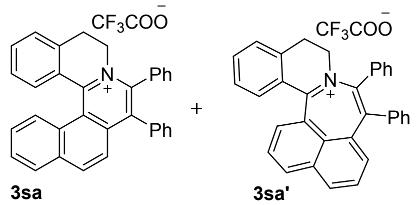

3sa 3sa' **4,5-diphenyl-1,2-dihydrobenzo[h]isoquinolino[1,2-a]isoquinolin-3-ium 2,2,2-trifluoroacetate (3sa)**. A yellow solid (167.5 mg, 95% yield). ^{1}H NMR (600 MHz, $CDCl_3$): δ 8.15 (d, J = 7.92, 1H), 7.94 (d, J = 7.92, 1H), 7.66 (t, J = 7.81, 2H), 7.58-7.52 (m, 8H), 7.45 (d, J = 0.96, 2H), 7.38-7.26 (m, 16H), 7.24-7.19 (m, 6H), 4.73 (d, J = 8.46 2H), 4.16 (t, J = 8.46, 2H), 3.81 (t, J = 7.92, 2H), 3.15 (d, J = 7.92H) ppm; ^{13}C NMR (150 MHz, $CDCl_3$): δ 151.7, 147.6, 141.2, 137.9, 137.1, 137.0, 134.1, 133.9, 133.8, 133.1, 131.7, 131.0, 131.0, 130.5, 130.3, 130.0, 129.9, 129.6, 129.6, 129.3, 129.0, 129.0, 128.7, 128.5, 128.2, 127.8, 127.5, 122.7, 53.2, 28.0 ppm. ESI HRMS: calcd. for $C_{33}H_{24}N^+$ $[M-CF_3COO]^+$ 434.19086, found 434.1926.

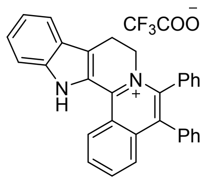

3ta **5,6-diphenyl-9,14-dihydro-8H-indolo[2',3':3,4]pyrido[2,1-a]isoquinolin-7-ium 2,2,2-trifluoroacetate (3ta)**. A yellow solid (120.8 mg, 86% yield). ^{1}H NMR (600 MHz, $CDCl_3$): δ 11.14 (s, 1H), 7.82 (t, J = 7.8, 1H), 7.72 (d, J = 8.4, 1H), 7.66(t, J = 7.8, 1H), 7.45 (d, J = 7.8, 1H), 7.30-7.35 (m, 3H), 7.29-7.26 (m, 2H), 7.24-7.22 (m, 5H), 7.11-7.09 (m, 3H), 6.85 (t, J = 7.2, 1H), 4.26 (t, J = 7.8, 2H), 3.13 (t, J = 7.2, 2H) ppm; ^{13}C NMR (150 MHz, $CDCl_3$): δ 150.7, 142.1, 137.1, 136.1, 135.5, 134.3, 133.6, 130.9, 130.0, 129.6, 129.3, 129.0, 128.3, 127.7, 126.4, 126.1, 125.4, 124.3, 121.2, 120.7, 120.3, 118.8, 53.7, 21.2 ppm. ESI HRMS: calcd. for $C_{31}H_{23}N_2^+$ $[M-CF_3COO]^+$ 423.18611, found 423.1879.

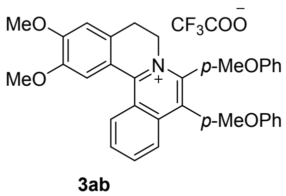

3ab **8,9-bis(4-methoxyl)-2,3-dimethoxy-5,6-dihydroisoquinolino[1,2-a]isoquinolin-7-ium 2,2,2-trifluoroacetate (3ab)**. A yellow solid (139.4 mg, 70% yield). ^{1}H

NMR (600 MHz, $CDCl_3$): δ 8.65 (d, *J* = 8.16, 1H), 7.95 (t, *J* = 7.80, 1H), 7.89 (t, *J* = 7.80, 1H), 7.77 (d, *J* = 8.40, 1H), 7.45 (s, 1H), 7.28 (s, 1H), 7.27 (s, 1H), 7.09-7.08 (m, 3H), 6.88-6.83 (m, 4H), 4.41 (s, 2H), 4.04 (s, 3H), 3.95 (s, 3H), 3.80 (d, 6H), 3.19 (t, *J* = 6.00, 2H) ppm; ^{13}C NMR (150 MHz, $CDCl_3$): δ 160.7, 159.5, 154.5, 153.8, 148.5, 144.7, 139.4, 136.3, 135.4, 133.6, 131.9, 131.8, 130.6, 130.2, 127.4, 126.1, 125.0, 123.8, 119.1, 116.1, 114.5, 114.1, 110.9, 56.8, 56.7, 56.5, 55.4, 52.3, 27.3 ppm. ESI HRMS: calcd. for $C_{33}H_{30}NO_4^+$ $[M-CF_3COO]^+$ 504.21746, found 504.2198.

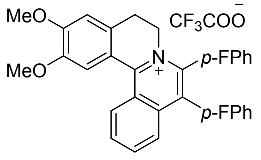

3ac

8,9-bis(4-fluorophenyl)-2,3-dimethoxy-5,6-dihydroisoquinolino[1,2-a]isoquinolin-7-ium 2,2,2-trifluoroacetate (3ac). A yellow solid (143.2 mg, 75% yield). ^1H NMR (600 MHz, $CDCl_3$): δ 8.67 (d, *J* = 7.86, 1H), 7.96 (t, *J* = 7.80, 1H), 7.90 (t, *J* = 7.80, 1H), 7.68 (d, *J* = 7.80, 1H), 7.57 (m, 2H), 7.51 (s, 1H), 7.24 (m, 2H), 7.07-7.00 (m, 5H), 4.47 (s, 2H), 4.04 (s, 3H), 3.97 (s, 3H), 3.23 (s, 2H) ppm; ^{13}C NMR (150 MHz, $CDCl_3$): δ 163.4 (d, *J* = 250.39 Hz), 162.6 (d, *J* = 247.71 Hz), 154.4, 148.5, 143.8, 139.0, 135.6, 135.4, 133.5, 133.0 (d, *J* = 8.55 Hz), 132.5 (d, *J* = 8.7 Hz), 130.9, 130.3, 129.9 (d, *J* = 4.83 Hz), 127.8, 127.0 (d, *J* = 4.5 Hz), 125.3, 119.2, 116.5 (d, *J* = 21.6 Hz), 116.3, 115.8 (d, *J* = 21.6 Hz), 110.7, 56.8, 56.7, 52.3, 27.3 ppm. ESI HRMS: calcd. for $C_{31}H_{24}F_2NO_2^+$ $[M-CF_3COO]^+$ 480.17749, found 480.1795.

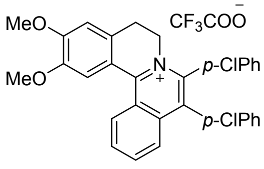

3ad

8,9-bis(4-chlorophenyl)-2,3-dimethoxy-5,6-dihydroisoquinolino[1,2-a]isoquinolin-7-ium 2,2,2-trifluoroacetate (3ad). A yellow solid (141.3 mg, 70% yield). ^1H NMR (600 MHz, $CDCl_3$): δ 8.66 (d, *J* = 9.00, 1H), 7.96 (t, *J* = 7.20, 1H), 7.90 (t, *J* = 7.86, 1H), 7.65 (d, *J* = 9.00, 1H), 7.45 (m, 3H), 7.34-7.30 (m, 4H), 7.17 (m, 2H), 7.03 (s, 1H), 4.38 (t, *J* = 6.00, 2H), 4.03 (s, 3H), 3.94 (s, 3H), 3.23 (t, *J* = 5.40, 2H) ppm; ^{13}C NMR (150 MHz, $CDCl_3$): δ 154.7, 154.5, 148.5, 143.2, 138.7, 136.7, 135.7, 135.0, 134.9, 133.5, 132.3, 132.0, 131.9, 130.8, 130.4, 130.0, 129.6, 129.1, 127.0, 125.2, 119.0, 116.1, 110.7, 56.7 52.3, 27.1 ppm. ESI HRMS: calcd. for $C_{31}H_{24}Cl_2NO_2^+$ $[M-CF_3COO]^+$ 512.11839, found 512.1198.

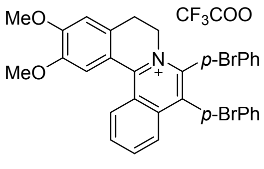

3ae

8,9-bis(4-chlorophenyl)-2,3-dimethoxy-5,6-dihydroisoquinolino[1,2-a]isoquinolin-7-ium 2,2,2-trifluoroacetate (3ae). A yellow solid (183.8 mg, 80% yield). ^1H NMR (600 MHz, $CDCl_3$): δ 8.65 (d, *J* = 9.00, 1H), 7.95 (t, *J* = 7.86, 1H), 7.89 (t, *J* = 7.80, 1H), 7.64 (d, *J* = 8.40, 1H), 7.50 (m, 2H), 7.47-7.42 (m, 5H), 7.14-7.12 (m, 2H), 7.00 (s, 1H), 4.38 (t, *J* = 6.30 2H), 4.03 (s, 3H), 3.94 (s, 3H), 3.19 (s, 2H) ppm; ^{13}C NMR (150 MHz, $CDCl_3$): δ 160.5, 160.2, 154.7, 154.5, 148.5, 143.1, 138.7, 135.7, 134.9, 133.3, 132.8, 132.6, 132.2, 132.0, 127.0, 125.2, 125.1, 123.2, 119.1, 116.2, 110.6, 56.7, 56.7, 52.2, 27.2 ppm. ESI HRMS: calcd. for $C_{31}H_{24}Br_2NO_2^+$ $[M-CF_3COO]^+$ 600.01747, found 600.0197.

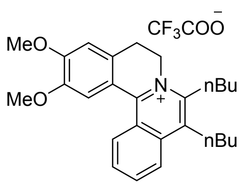

3af **8,9-dibutyl-2,3-dimethoxy-5,6-dihydroisoquinolino[1,2-a]isoquinolin-7-ium 2,2,2-trifluoroacetate (3af)**. A yellow solid (165.1 mg, 99% yield). ^{1}H NMR (600 MHz, $CDCl_3$): δ 8.50 (d, *J* = 8.46, 1H), 8.18 (d, *J* = 7.86, 1H), 8.06 (d, *J* = 8.1, 1H), 7.84 (t, J = 7.8, 1H), 7.21 (s, 1H), 7.14 s, 1H), 4.88 (s, 2H), 4.05 (s, 3H), 3.89 (s, 3H), 3.35 (m, 4H), 3.13(t, J = 8.46, 2H), 1.70-1.60 (m, 8H), 1.06-0.99 (m, 6H) ppm; ^{13}C NMR (150 MHz, $CDCl_3$): δ 154.3, 153.1, 148.3, 145.5, 138.3, 135.5, 133.8, 133.5, 131.2, 129.4, 124.5, 124.2, 118.8, 115.9, 110.9, 56.7, 50.2, 32.4, 31.1, 29.6, 28.8, 27.3, 22.9, 13.9, 13.8 ppm. ESI HRMS: calcd. for $C_{27}H_{34}NO_2^+$ [M-CF_3COO]$^+$ 404.25893, found 404.2599.

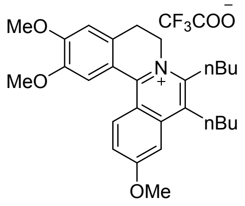

3bf **8,9-dibutyl-2,3,11-trimethoxy-5,6-dihydroisoquinolino[1,2-a]isoquinolin-7-ium 2,2,2-trifluoroacetate (3bf)**. A yellow solid (167.8 mg, 95% yield). ^{1}H NMR (600 MHz, $CDCl_3$): δ 8.38 (d, *J* = 10.14, 1H), 7.40 (d, *J* = 9.54, 1H), 7.32 (s, 1H), 7.14-7.13 (m, 2H), 4.74 (s, 2H), 4.07 (s, 3H), 4.00 (s, 3H), 3.88 (s, 3H), 3.32-3.25 (m, 4H), 3.06 (t, *J* = 8.52, 2H)), 1.68 (m, 4H), 1.60-1.55 (m, 4H), 1.03-1.01 (m, 6H) ppm; ^{13}C NMR (150 MHz, $CDCl_3$): δ 165.1, 153.9, 151.8, 148.2, 145.4, 140.9, 133.6, 133.2, 131.5, 121.6, 119.5, 118.8, 115.6, 110.9, 103.1, 56.7, 56.3, 49.5, 31.6, 31.0, 29.6, 28.7, 27.4, 23.2, 22.9, 13.9, 13.8 ppm. ESI HRMS: calcd. for $C_{28}H_{36}NO_3^+$ [M-CF_3COO]$^+$ 434.26950, found 434.2699.

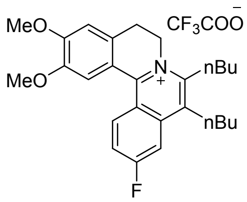

3df **8,9-dibutyl-11-fluoro-2,3-dimethoxy-5,6-dihydroisoquinolino[1,2-a]isoquinolin-7-ium 2,2,2-trifluoroacetate (3df).** A yellow solid (162.7 mg, 94% yield). ^{1}H NMR (600 MHz, $CDCl_3$): δ 8.54 (q, *J* = 7.2, 1H), 7.72 (d, *J* = 7.92, 1H), 7.56 (t, *J* = 8.1, 1H), 7.15-7.12 (m, 2H), 4.84 (s, 2H), 4.04 (s, 3H), 3.88 (s, 3H), 3.34-3.30 (m, 4H), 3.04 (t, *J* = 8.1, 2H)), 1.67-1.59 (m, 8H), 1.05-1.01 (m, 6H) ppm; ^{13}C NMR (150 MHz, $CDCl_3$): δ 166.5 (d, *J* = 260.86 Hz), 154.5, 153.1, 148.4, 146.4, 140.8 (d, *J* = 11.2 Hz), 135.0 (d, *J* = 10.2 Hz), 134.0, 132.6 (d, *J* = 5.7 Hz), 121.7, 120.0 (d, *J* = 25.8 Hz), 119.7, 115.7, 110.9, 108.6 (d, *J* = 22.3 Hz), 56.6, 50.0, 32.1, 30.9, 29.7, 29.0, 27.2, 23.3, 22.9, 13.9, 13.8 ppm. ESI HRMS: calcd. for $C_{27}H_{33}FNO_2^+$ [M-CF_3COO]$^+$ 422.24951, found 422.2508.

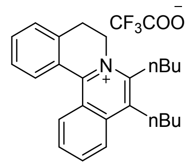

3qf

8,9-dibutyl-5,6-dihydroisoquinolino[1,2-a]isoquinolin-7-ium 2,2,2-trifluoroacetate (3qf). A yellow solid (125.3 mg, 85% yield). ^{1}H NMR (600 MHz, $CDCl_3$): δ 8.42 (d, *J* = 7.86, 1H), 8.18 (d, *J* = 9.06, 1H), 8.08 (m, 1H), 7.82 (t, *J* = 7.86, 1H), 7.74 (d, *J* = 7.26, 1H), 7.63 (m, 1H), 7.54-7.50 (m, 2H), 4.89 (s, 2H), 3.34 (s, 4H)), 3.12 (t, *J* = 7.56, 8H), 1.67-1.54 (m, 8H), 0.99-0.97 (m, 6H) ppm; ^{13}C NMR (150 MHz, $CDCl_3$): δ 153.0, 145.6, 138.6, 138.2, 136.0, 135.0, 133.7, 133.0, 131.0, 130.1, 129.8, 128.0, 127.5, 126.6, 124.5, 124.2, 50.3, 32.3, 30.9, 29.5, 29.1, 28.8, 27.7, 23.2, 22.8, 13.7, 13.7 ppm. ESI HRMS: calcd. for $C_{25}H_{30}N^{+}$ [M-CF_3COO]$^{+}$ 344.23781, found 344.2398.

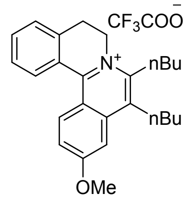

3of

8,9-dibutyl-11-methoxy-5,6-dihydroisoquinolino[1,2-a]isoquinolin-7-ium 2,2,2-trifluoroacetate (3of). A yellow solid (143.2 mg, 91% yield). ^{1}H NMR (600 MHz, $CDCl_3$): δ 8.35 (d, *J* = 9.60, 1H), 7.70 (d, *J* = 7.80, 1H), 7.63 (t, *J* = 7.20, 1H), 7.55-7.50 (m, 2H), 7.42 (d, *J* = 9.60, 1H), 7.34 (d, *J* = 2.40, 1H), 4.77 (s, 2H), 4.07 (s, 3H)), 3.29 (m, 4H), 3.07 (t, *J* = 7.80, 2H), 1.70-1.56 (m, 8H),1.05-0.99 (m, 6H) ppm; ^{13}C NMR (150 MHz, $CDCl_3$): δ 165.3, 151.7, 145.8, 141.0, 138.7, 133.5, 133.3, 132.8, 128.1, 127.6, 126.8, 122.3, 119.9, 103.0, 56.3, 49.7, 31.6, 31.0, 29.6, 28.9, 28.0, 23.2, 22.9, 13.8, 13.8 ppm. ESI HRMS: calcd. for $C_{26}H_{32}NO^{+}$ [M-CF_3COO]$^{+}$ 374.24837, found 374.2499.

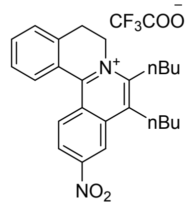

3pf

8,9-dibutyl-11-methoxy-5,6-dihydroisoquinolino[1,2-a]isoquinolin-7-ium 2,2,2-trifluoroacetate (3pf). A yellow solid (143.8 mg, 89% yield). ^{1}H NMR (600 MHz, $CDCl_3$): δ 9.03 (d, *J* = 2.40, 1H), 8.65 (d, *J* = 9.60, 1H), 8.47 (d, *J* = 9.60, 1H), 7.75-7.70 (m, 2H), 7.59-7.56 (m, 2H), 5.01 (s, 2H), 3.42 (m, 4H)), 3.23 (m, 2H), 1.74 (m, 4H), 1.63 (m, 4H), 1.08-1.02 (m, 6H) ppm; ^{13}C NMR (150 MHz, $CDCl_3$): δ 154.0, 150.9, 148.7, 139.0, 138.5, 136.8, 134.6, 134.1, 133.2, 128.2, 128.0, 127.1, 126.6, 122.5, 120.0, 51.0, 32.7, 30.8, 30.0, 29.3, 27.5, 23.2, 23.0, 13.8 ppm. ESI HRMS: calcd. for $C_{25}H_{29}N_2O_2^{+}$ [M-CF_3COO]$^{+}$ 389.22288, found 389.2253.

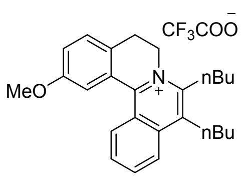

3rf

8,9-dibutyl-2-methoxy-5,6-dihydroisoquinolino[1,2-a]isoquinolin-7-ium 2,2,2-trifluoroacetate (3rf). A yellow solid (154.2 mg, 98% yield). ^{1}H NMR (600 MHz, $CDCl_3$): δ 8.54 (d, *J* = 8.40, 1H), 8.21 (d, *J* = 8.40, 1H), 8.10 (t, *J* = 7.86, 1H), 7.83 (t, *J* = 7.20, 2H), 7.50 (d, *J* = 7.80 1H), 7.27 (m, 1H), 7.21 (m, 1H), 4.90 (s, 2H), 3.86 (s, 3H), 3.37-3.29 (m, 4H), 3.16 (t, *J* = 8.40, 2H), 1.70-1.59 (m, 8H), 1.05-0.98 (m, 6H) ppm; ^{13}C NMR (150 MHz, $CDCl_3$): δ 158.6, 153.0, 146.0, 138.4, 135.6, 131.1, 130.6, 129.9, 127.5, 124.7, 124.2, 118.9, 56.0, 51.0, 49.5, 32.4, 31.0, 29.6, 28.9, 27.0, 23.3, 22.9, 13.9, 13.8 ppm. ESI HRMS: calcd. for $C_{26}H_{32}NO^+$ [M-$CF_3COO]^+$ 374.24837, found 374.2497.

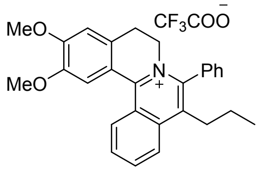

3ag **2,3-dimethoxy-9-phenyl-8-propyl-5,6-dihydroisoquinolino[1,2-a]isoquinolin-7-ium 2,2,2-trifluoroacetate (3ag).** A yellow solid (165.6 mg, 95% yield). ^{1}H NMR (600 MHz, $CDCl_3$): δ 8.64 (d, *J* = 7.80, 1H), 8.26 (d, *J* = 7.20, 1H), 8.15 (m, 1H), 7.93 (m, 1H), 7.65 (s, 3H), 7.51 (s, 2H), 7.37 (s, 1H), 7.11 (s, 1H), 4.28 (s, 2H), 4.03 (s, 3H), 3.93 (s, 3H), 3.20 (s, 2H), 2.78 (t, *J* = 7.80, 2H), 1.52 (d, *J* = 6.66, 2H), 0.88 (t, *J* = 7.20, 8H) ppm; ^{13}C NMR (150 MHz, $CDCl_3$): δ 154.4, 153.2, 148.4, 138.2, 135.8, 134.8, 131.8, 131.4, 131.0, 130.2, 130.1, 129.7, 125.2, 125.0, 118.8, 116.0, 111.0, 56.8, 52.4, 31.9, 27.1, 23.9, 14.6 ppm. ESI HRMS: calcd. for $C_{28}H_{28}NO_2^+$ [M-$CF_3COO]^+$ 410.21199, found 410.2135.

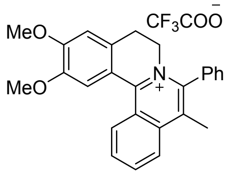

3ah

inolino[1,2-a]isoquinolin-7-ium 2,2,2-trifluoroacetate (3ah). A yellow solid (155.0 mg, 97% yield). ^{1}H NMR (600 MHz, $CDCl_3$): δ 8.62 (d, *J* = 7.86, 1H), 8.27 (d, *J* = 8.40, 1H), 8.16 (d, *J* = 6.60, 1H), 7.93 (t, *J* = 7.20 1H), 7.63 (s, 3H), 7.50 (s, 2H), 7.37 (s, 1H), 7.06 (s, 1H), 4.29 (s, 2H), 4.03 (s, 3H), 3.93 (s, 3H), 3.14 (s, 2H), 2.47 (s, 3H) ppm; ^{13}C NMR (150 MHz, $CDCl_3$): δ 154.3, 153.1, 148.4, 143.6, 138.8, 135.9, 133.1, 132.0, 131.1, 131.0, 130.5, 130.4, 130.2, 129.6, 125.0, 124.7, 118.8, 116.0, 110.8 56.7, 52.4, 27.1, 16.7 ppm. ESI HRMS: calcd. for $C_{26}H_{24}NO_2^+$ [M-$CF_3COO]^+$ 382.18069, found 382.1823.

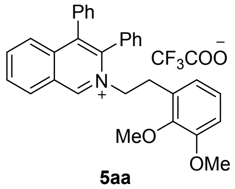

5aa

2-(2,3-dimethoxyphenethyl)-3,4-diphenylisoquinolin-2-ium 2,2,2-trifluoroacetate (5aa). A light yellow solid (99.3 mg, 55% yield). ^{1}H NMR (600 MHz, $CDCl_3$): δ 11.26 (s, 1H), 8.84 (s, 1H), 8.00 (t, *J* = 8.40, 1H), 7.93 (t, *J* = 7.20, 1H), 7.62 (d, *J* = 8.40 1H), 7.42-7.37 (m, 3H), 7.31 (m, 3H), 7.18 (d, *J* = 7.20, 2H), 7.07 (m, 2H), 6.62-6.21 (m, 2H), 6.30 (m, *J* = 8.40, 1H), 4.96 (s, 2H), 3.77 (s, 3H), 3.75 (s, 3H), 3.12 (s, 2H), 1.70-1.59 (m, 8H), 1.05-0.98 (m, 6H) ppm; ^{13}C NMR (150 MHz, $CDCl_3$): δ 152.7, 149.3, 148.3, 143.6, 139.2, 137.6, 137.4, 133.2, 131.2, 130.8, 130.6, 130.4, 129.0, 128.7, 127.9, 126.1, 120.8, 112.2, 111.3, 60.4, 56.0, 37.6 ppm. ESI HRMS: calcd. for $C_{31}H_{28}NO_2^+$ 446.21199, found 446.2135.

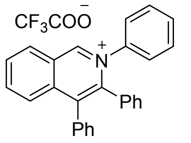

5ba

2,3,4-triphenylisoquinolin-2-ium 2,2,2-trifluoroacetate (5ba). A white solid (76.2 mg, 50% yield). ^{1}H NMR (600 MHz, $CDCl_3$): δ 10.60 (s, 1H), 8.93 (s, 1H), 8.07 (t, *J* = 7.80, 1H), 8.00 (s, 1H), 7.77 (d, *J* = 9.00 1H), 7.55 (s, 2H), 7.36-7.33 (m, 6H), 7.22-7.20 (m, 2H), 7.09-7.01 (m, 5H), 6.62-6.21 (m, 2H), 6.30 (m, *J* = 8.40, 1H), 4.96 (s, 2H), 3.77 (s, 3H), 3.75 (s, 3H), 3.12 (s, 2H), 1.70-1.59 (m, 8H), 1.05-0.98 (m, 6H) ppm; ^{13}C NMR (150 MHz, $CDCl_3$): δ 152.7, 144.1, 142.2, 139.0, 138.5, 138.0, 133.2, 132.8, 131.4, 131.2, 130.6, 130.4, 129.7, 129.4, 128.9, 128.7, 128.1, 126.9, 126.4 ppm. ESI HRMS: calcd. for $C_{27}H_{20}N^+$ $[M-CF_3COO]^+$ 358.15956, found 358.1611.

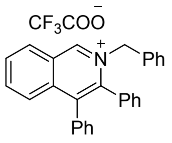

5ca

2-benzyl-3,4-diphenylisoquinolin-2-ium 2,2,2-trifluoroacetate (5ca). A white solid (109.5 mg, 70% yield). ^{1}H NMR (600 MHz, $CDCl_3$): δ 11.29 (s, 1H), 8.91 (d, *J* = 8.40, 1H), 8.01 (t, *J* = 7.80, 1H), 7.96 (t, *J* = 7.80, 1H), 7.64 (d, *J* = 8.40 1H), 7.32 (t, *J* = 7.20, 1H), 7.28-7.26 (m, 3H), 7.25-7.22 (m, 3H), 7.21-7.18 (m, 2H), 7.06-7.05 (m, 2H), 7.01 (s, 1H), 7.00 (s, 1H), 6.90 (s, 1H), 6.89 (s, 1H), 6.06 (s, 2H) ppm; ^{13}C NMR (150 MHz, $CDCl_3$): δ 153.1, 144.1, 139.6, 138.0, 137.5, 133.6, 133.2, 132.5, 131.2, 130.8, 130.7, 130.2, 130.1, 129.2, 129.1, 128.8, 128.7, 128.6, 128.3, 127.6, 126.3, 63.1 ppm. ESI HRMS: calcd. for $C_{28}H_{22}N^+$ $[M-CF_3COO]^+$ 372.17521, found 372.1767.

5. Reference

1. Mio, M. J.; Kopel, L. C.; Braun, J. B.; Gadzikwa, T. L.; Hull, K. L.; Brisbois, R. G.; Markworth, C. J.; Grieco, P. A., *Org. Lett.* 2002, **4**, 3199.
2. Perez, M.; Wu, Z.; Scalone, M.; Ayad, T.; Ratovelomanana-Vidal, V., *Eur. J. Org. Chem.* 2015, **2015**, 6503.

6. NMR Spectra

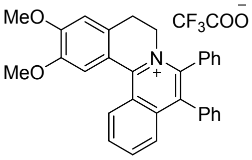

3aa

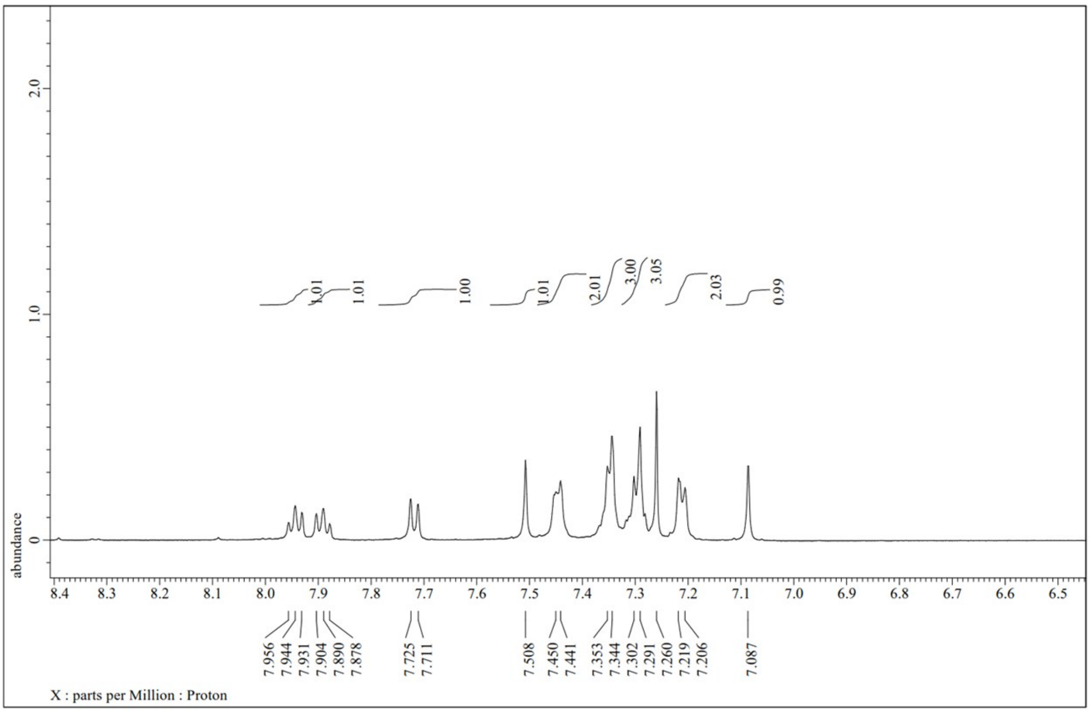

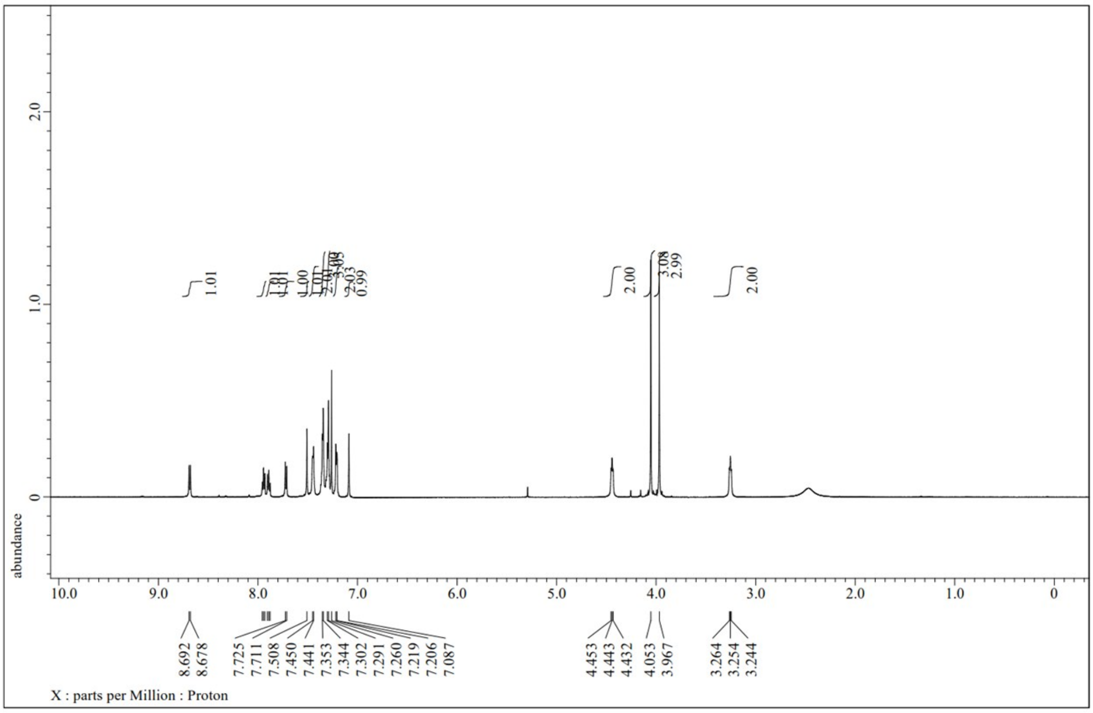

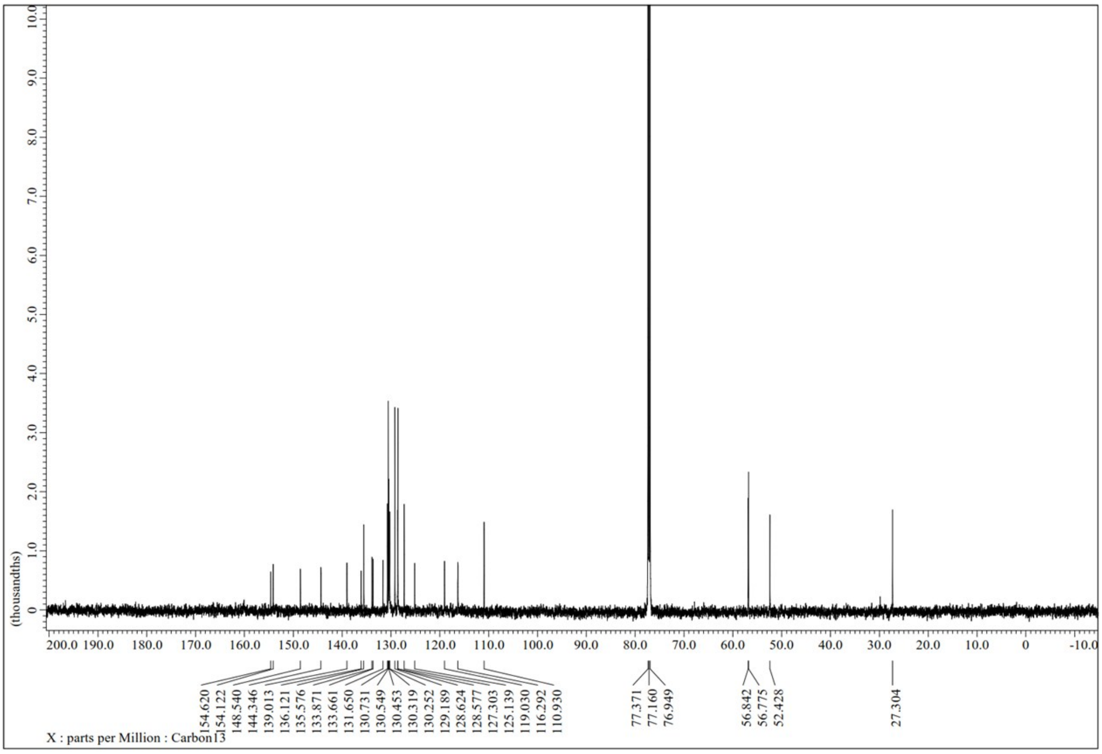
(thousandths)
154.620
154.122
148.540
144.346
139.013
136.121
135.576
133.871
133.661
131.650
130.731
130.549
130.453
130.319
130.252
129.189
128.624
128.577
127.303
125.139
119.030
116.292
110.930
77.371
77.160
76.949
56.842
56.775
52.428
27.304
X : parts per Million : Carbon13

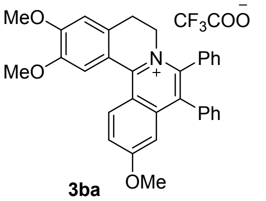
MeO
MeO
CF_3COO^-
N
Ph
Ph
OMe

3ba

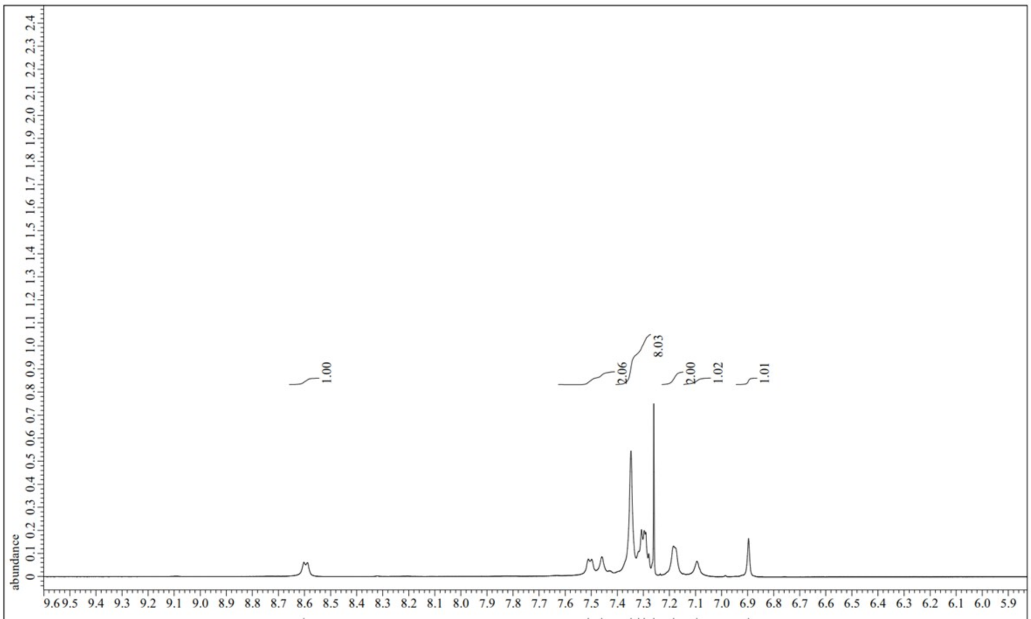
abundance
1.00
2.06
8.03
2.00
1.02
1.01
8.602
7.511
7.460
7.347
7.319
7.297
7.260
7.184
7.095
6.896
X : parts per Million : Proton

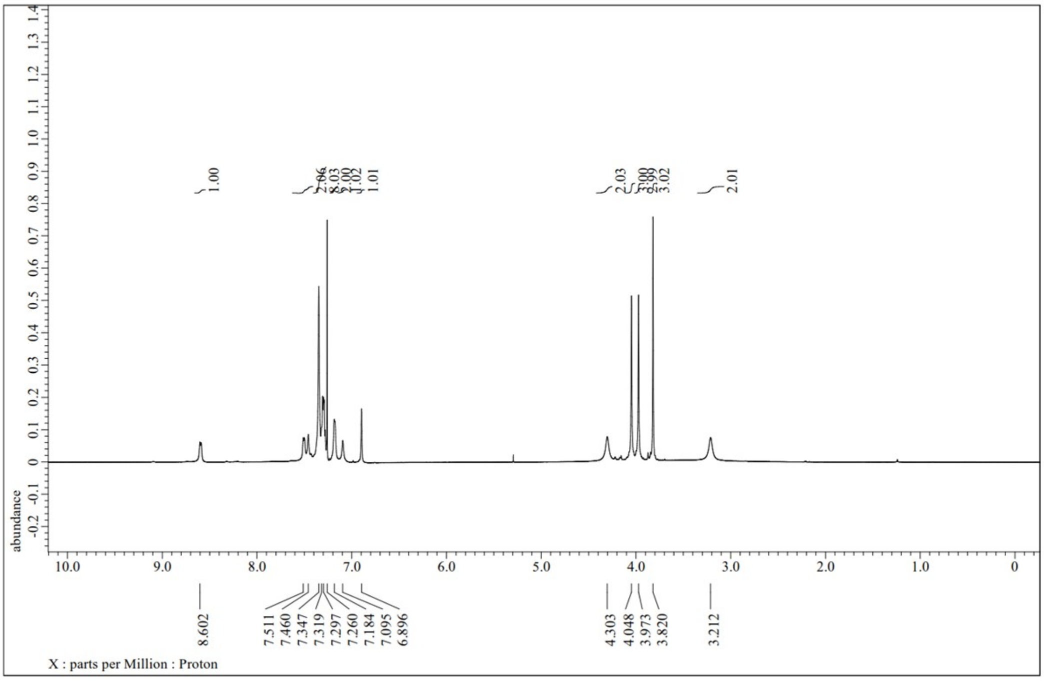
1.00
2.06
2.03
2.00
1.02
1.01
2.03
3.00
2.99
3.02
2.01
8.602
7.511
7.460
7.347
7.319
7.297
7.260
7.184
7.095
6.896
4.303
4.048
3.973
3.820
3.212
abundance
X : parts per Million : Proton

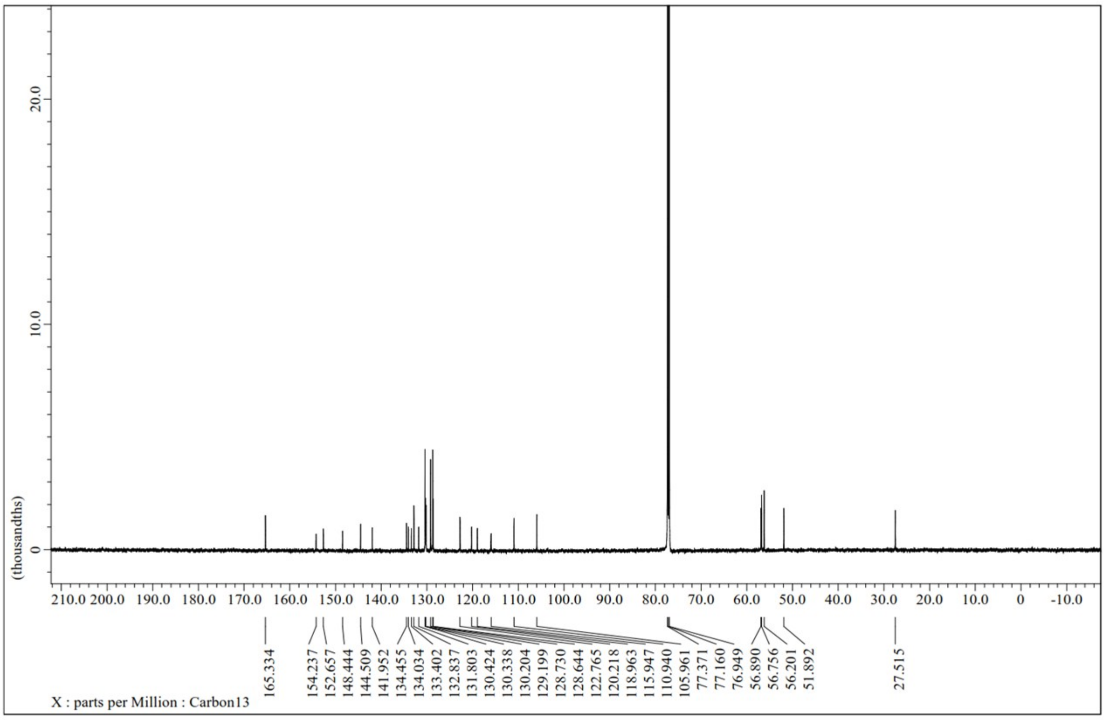
(thousandths)
165.334
154.237
152.657
148.444
144.509
141.952
134.455
134.034
133.402
132.837
131.803
130.424
130.338
130.204
129.199
128.730
128.644
122.765
120.218
118.963
115.947
110.940
105.961
77.371
77.160
76.949
56.890
56.756
56.201
51.892
27.515
X : parts per Million : Carbon13

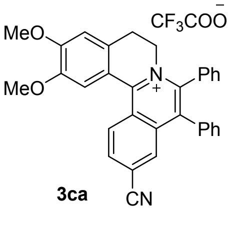
MeO
MeO
CF_3COO^-
N
+
Ph
Ph
CN

3ca

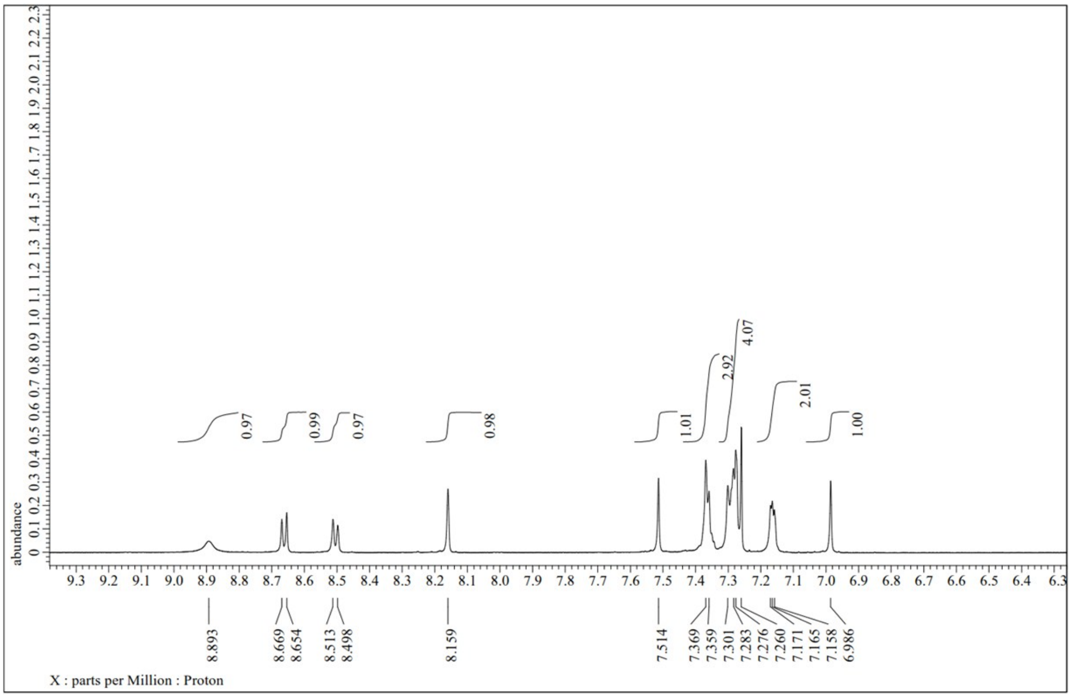
abundance
X : parts per Million : Proton
0.97
0.99
0.97
0.98
1.01
2.92
4.07
2.01
1.00
8.893
8.669
8.654
8.513
8.498
8.159
7.514
7.369
7.359
7.301
7.283
7.276
7.260
7.171
7.165
7.158
6.986

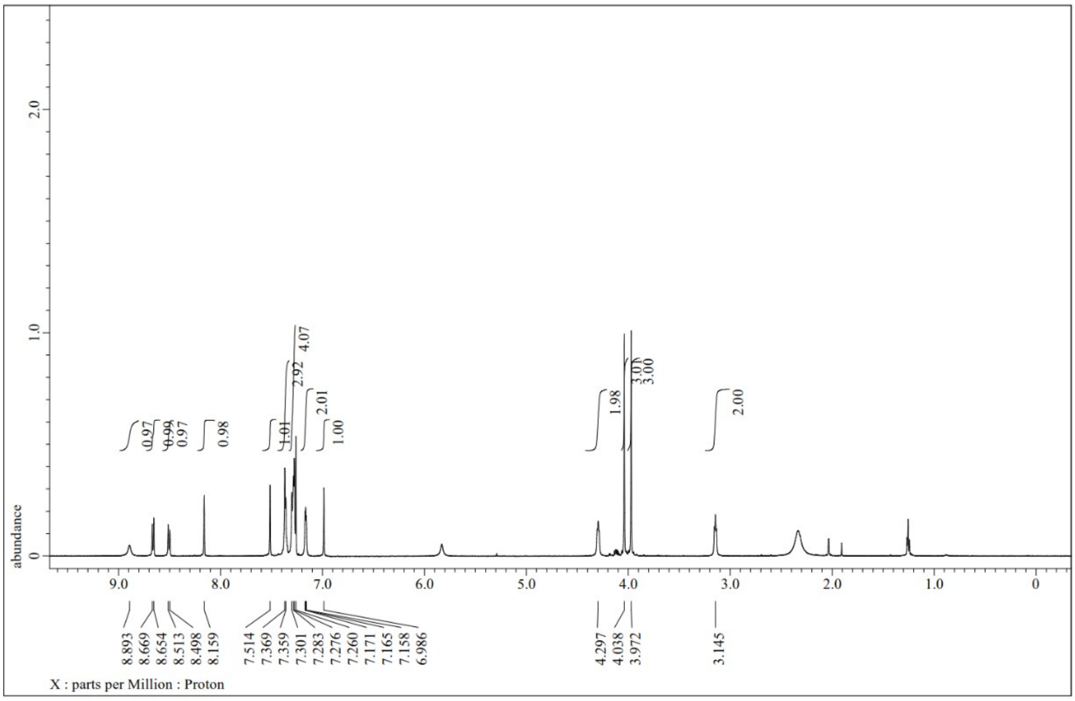
abundance
X : parts per Million : Proton
1.98
3.01
3.00
2.00
4.297
4.038
3.972
3.145

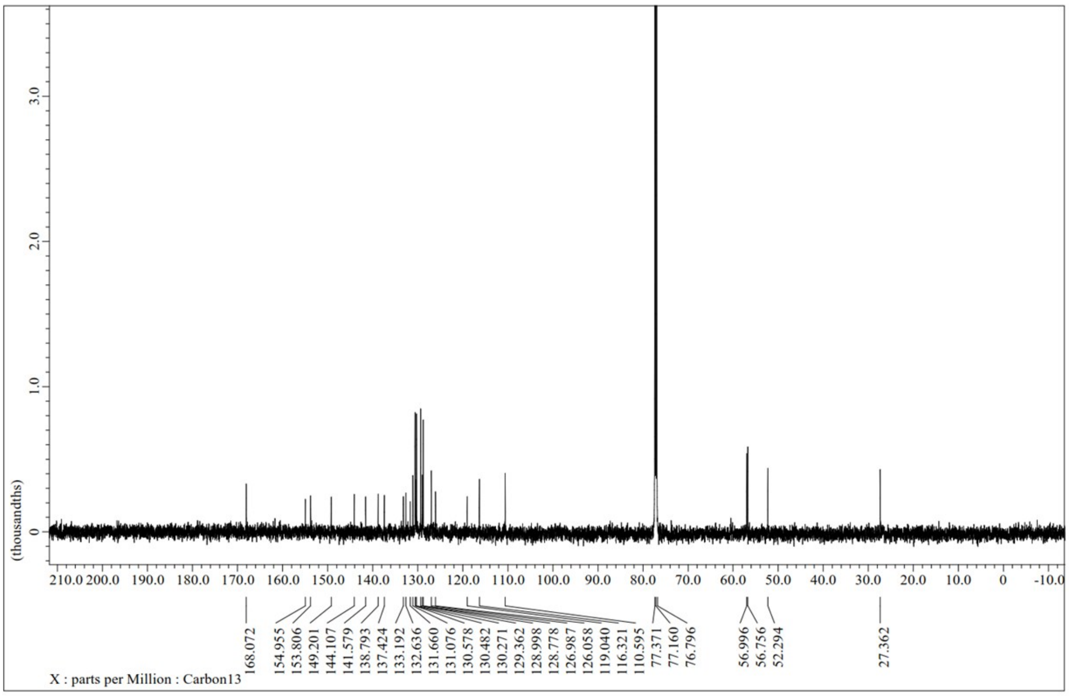
(thousandths)
X : parts per Million : Carbon13
168.072
154.955
153.806
149.201
144.107
141.579
138.793
137.424
133.192
132.636
131.660
131.076
130.578
130.482
130.271
129.362
128.998
128.778
126.987
126.058
119.040
116.321
110.595
77.371
77.160
76.796
56.996
56.756
52.294
27.362

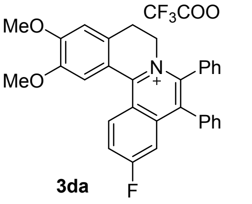
MeO
MeO
CF3COO
N
Ph
Ph
F

3da

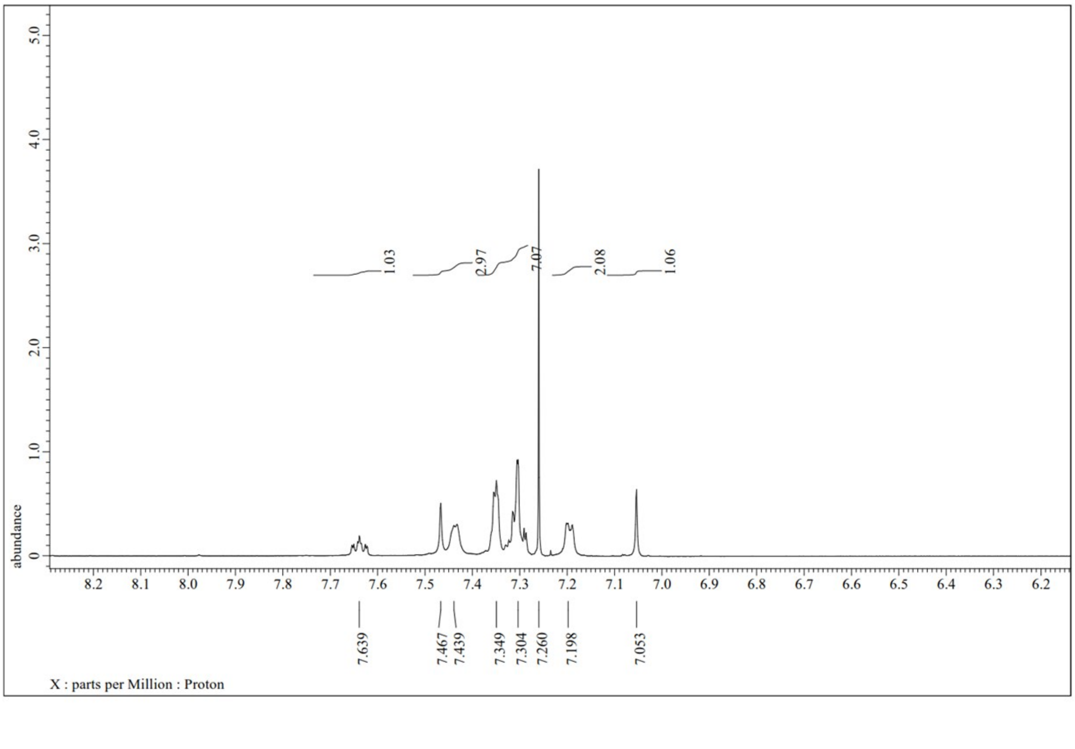
abundance
1.03
2.97
7.07
2.08
1.06
X : parts per Million : Proton
7.639
7.467
7.439
7.349
7.304
7.260
7.198
7.053

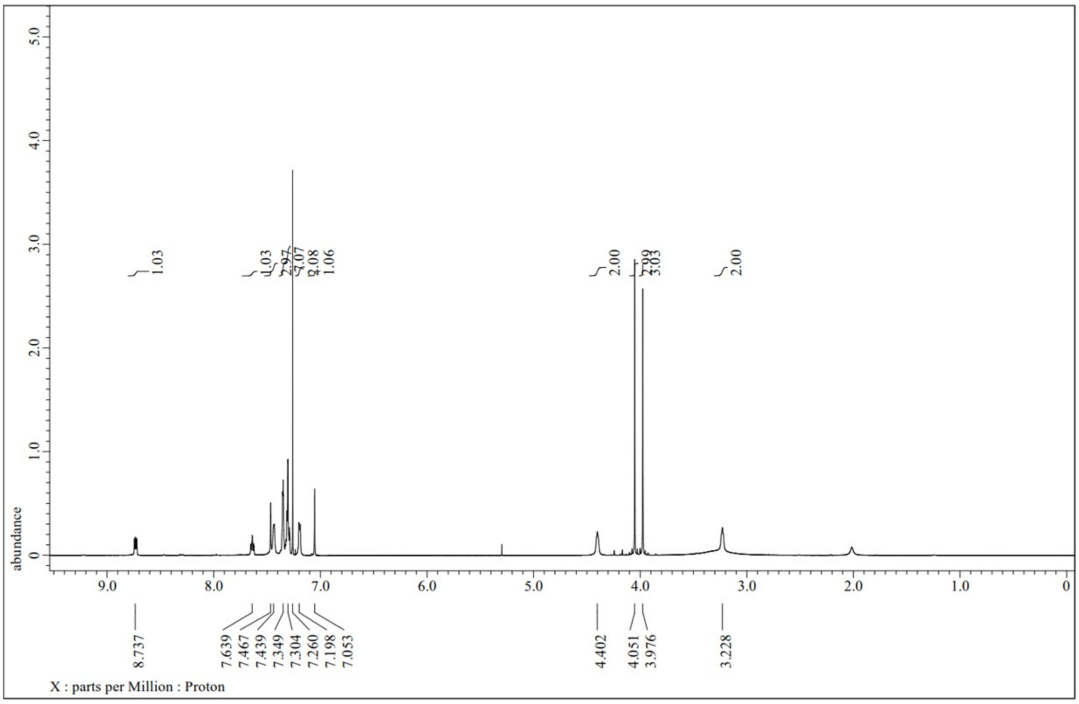
abundance
X : parts per Million : Proton
8.737
7.639
7.467
7.439
7.349
7.304
7.260
7.198
7.053
4.402
4.051
3.976
3.228
1.03
2.00
3.03
2.00

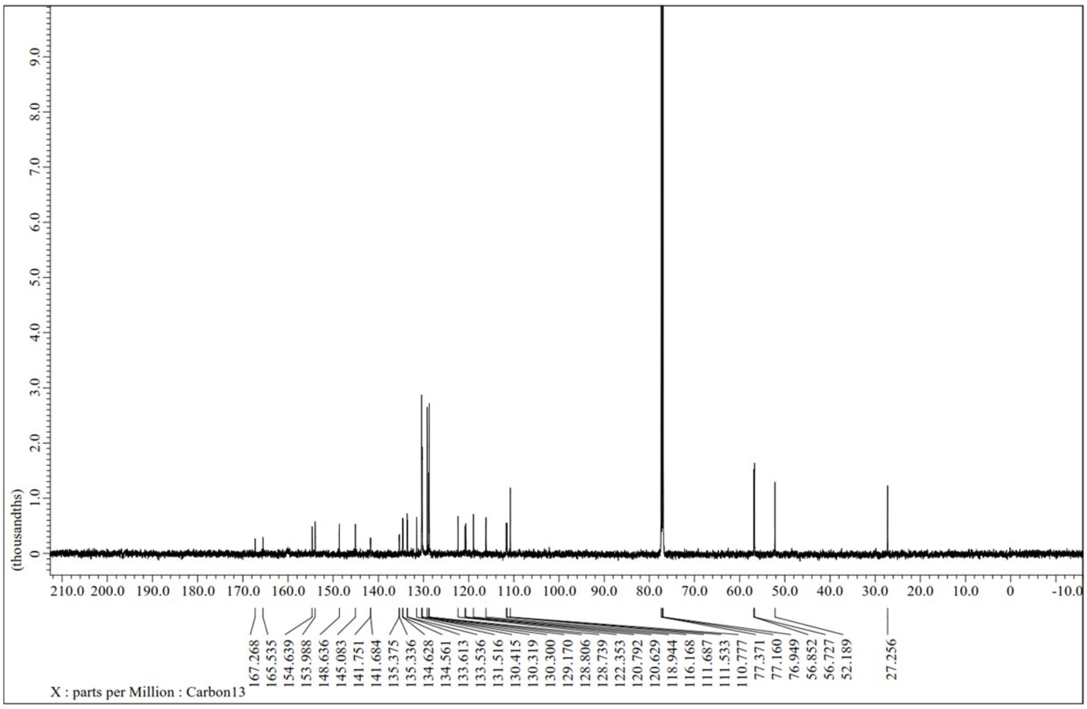
(thousandths)
X : parts per Million : Carbon13
167.268
165.535
154.639
153.988
148.636
145.083
141.751
141.684
135.375
135.336
134.628
134.561
133.613
133.536
131.516
130.415
130.319
130.300
129.170
128.806
128.739
122.353
120.792
120.629
118.944
116.168
111.687
111.533
110.777
77.371
77.160
76.949
56.852
56.727
52.189
27.256

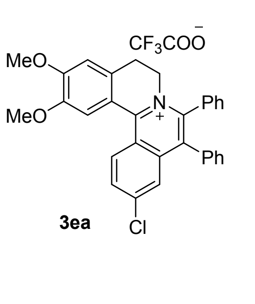
MeO
MeO
CF3COO
N
Ph
Ph
Cl

3ea

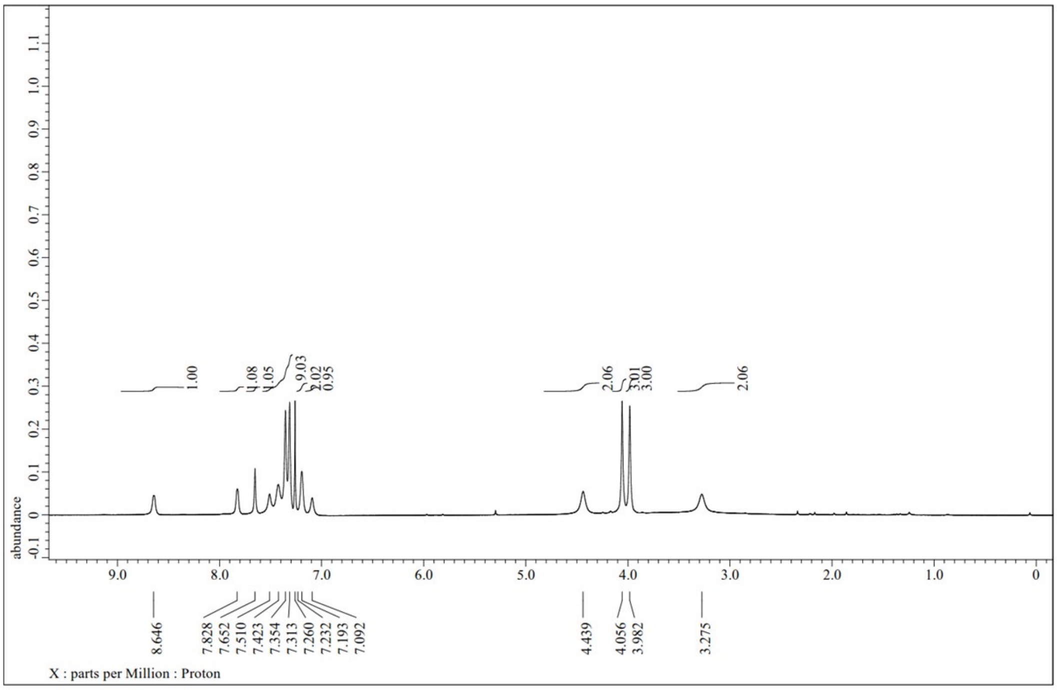

1.00
1.08
1.05
9.03
2.02
0.95
2.06
3.01
3.00
2.06
abundance
8.646
7.828
7.652
7.510
7.423
7.354
7.313
7.260
7.232
7.193
7.092
4.439
4.056
3.982
3.275
X : parts per Million : Proton

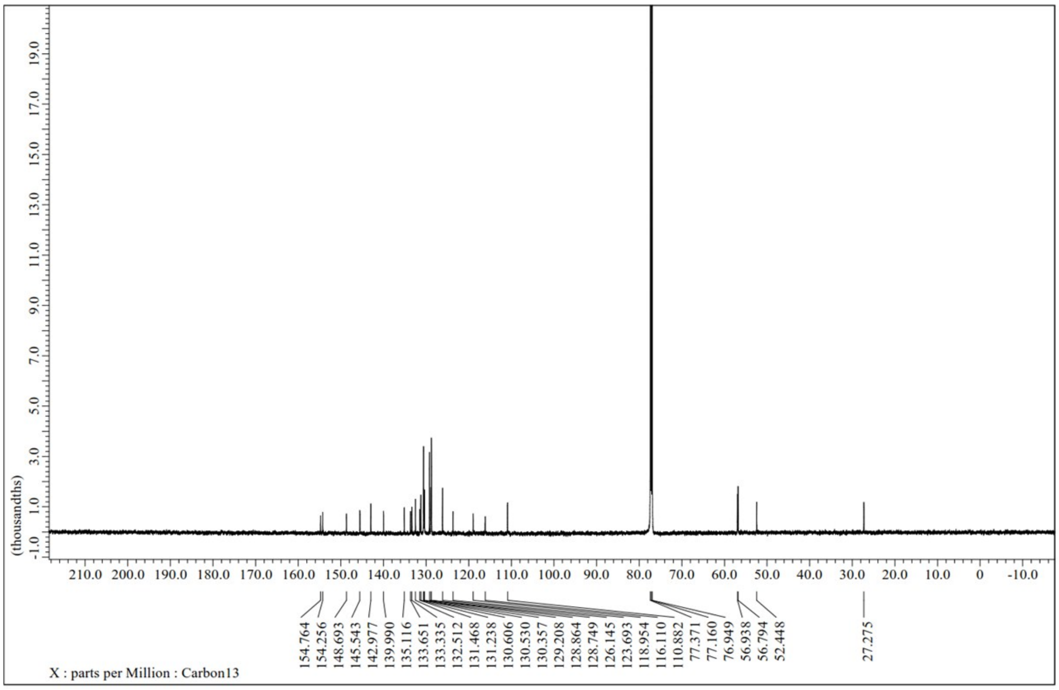

(thousandths)
154.764
154.256
148.693
145.543
142.977
139.990
135.116
133.651
133.335
132.512
131.468
131.238
130.606
130.530
130.357
129.208
128.864
128.749
126.145
123.693
118.954
116.110
110.882
77.371
77.160
76.949
56.938
56.794
52.448
27.275
X : parts per Million : Carbon13

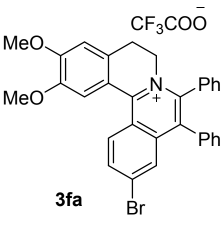

CF3COO
MeO
MeO
N
+
Ph
Ph
Br

3fa

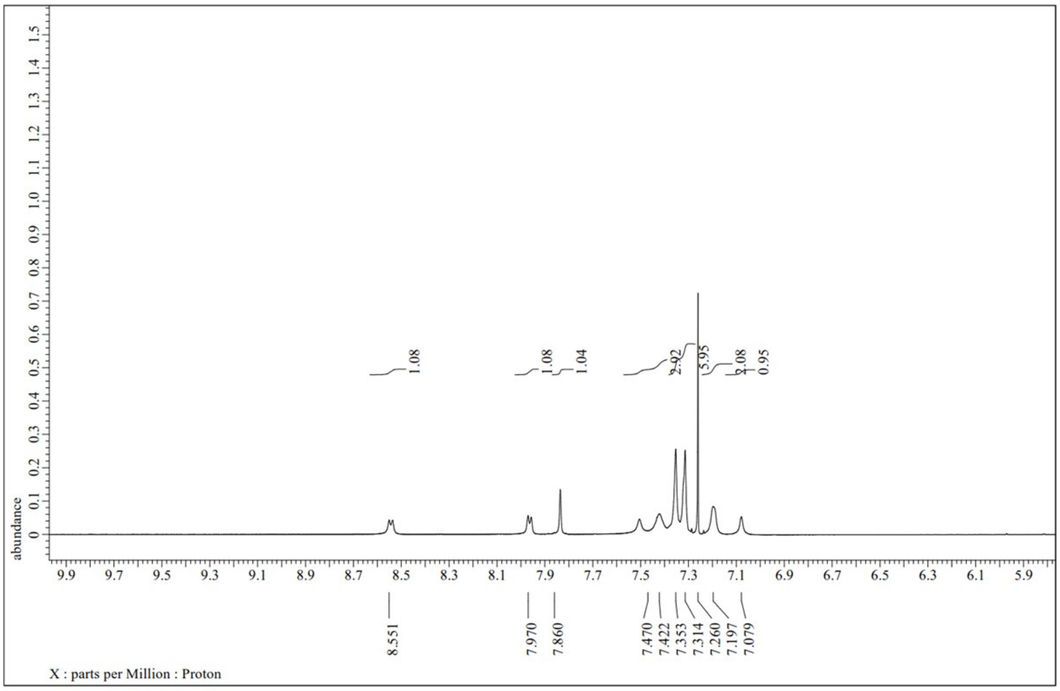
abundance
X : parts per Million : Proton
1.08
1.08
1.04
2.92
5.95
2.08
0.95
8.551
7.970
7.860
7.470
7.422
7.353
7.314
7.260
7.197
7.079

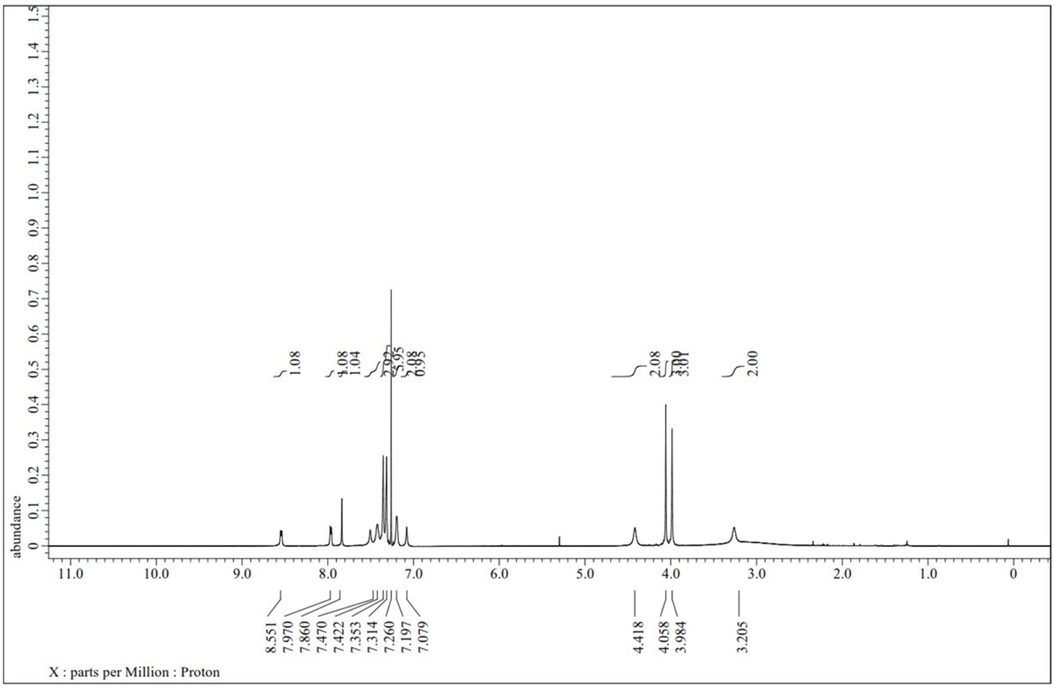
abundance
X : parts per Million : Proton
1.08
1.08
1.04
2.92
5.95
2.08
0.95
2.08
3.00
3.01
2.00
8.551
7.970
7.860
7.470
7.422
7.353
7.314
7.260
7.197
7.079
4.418
4.058
3.984
3.205

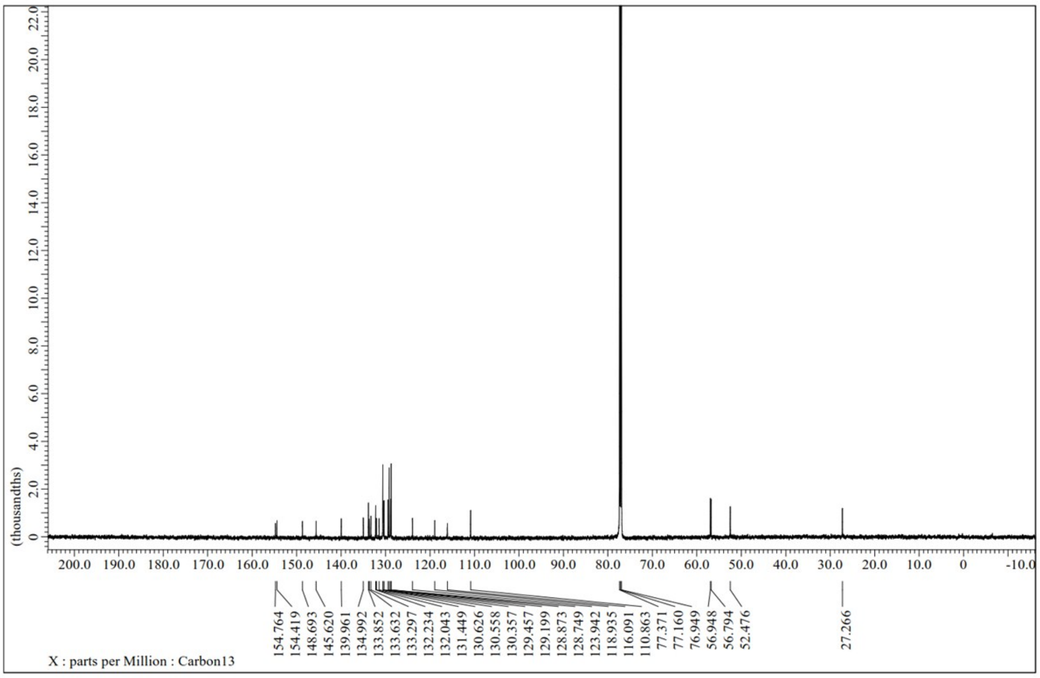
(thousandths)
X : parts per Million : Carbon13
154.764
154.419
148.693
145.620
139.961
134.992
133.852
133.632
133.297
132.234
132.043
131.449
130.626
130.558
130.357
129.457
129.199
128.873
128.749
123.942
118.935
116.091
110.863
77.371
77.160
76.949
56.948
56.794
52.476
27.266

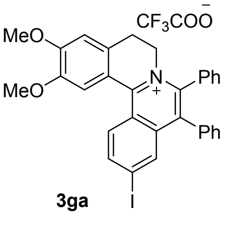
CF_3COO^-
MeO
MeO
N
Ph
Ph
I

3ga

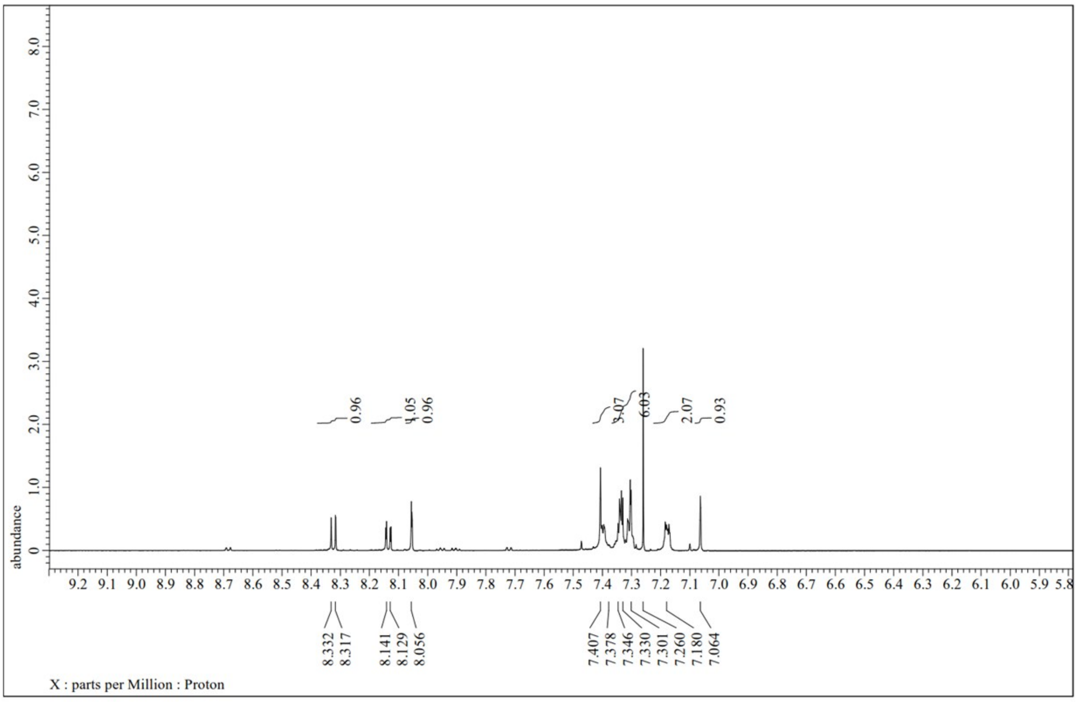
abundance
X : parts per Million : Proton
0.96
1.05
0.96
3.07
6.03
2.07
0.93
8.332
8.317
8.141
8.129
8.056
7.407
7.378
7.346
7.330
7.301
7.260
7.180
7.064

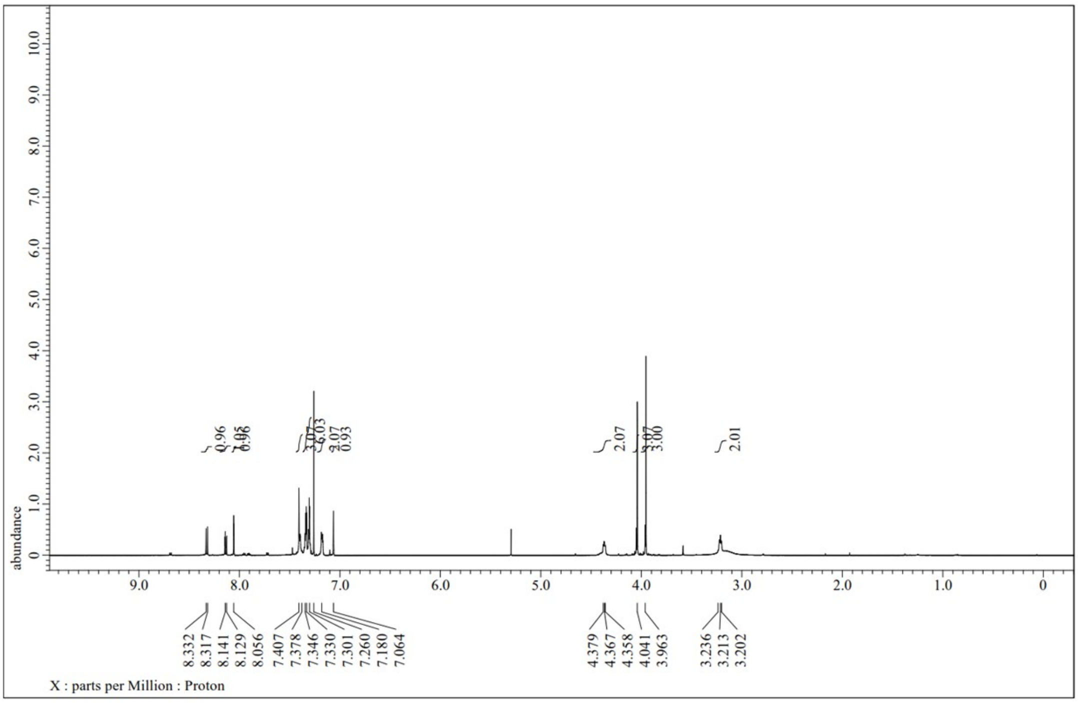
abundance
X : parts per Million : Proton
8.332
8.317
8.141
8.129
8.056
7.407
7.378
7.346
7.330
7.301
7.260
7.180
7.064
4.379
4.367
4.358
4.041
3.963
3.236
3.213
3.202
2.07
2.01
3.00

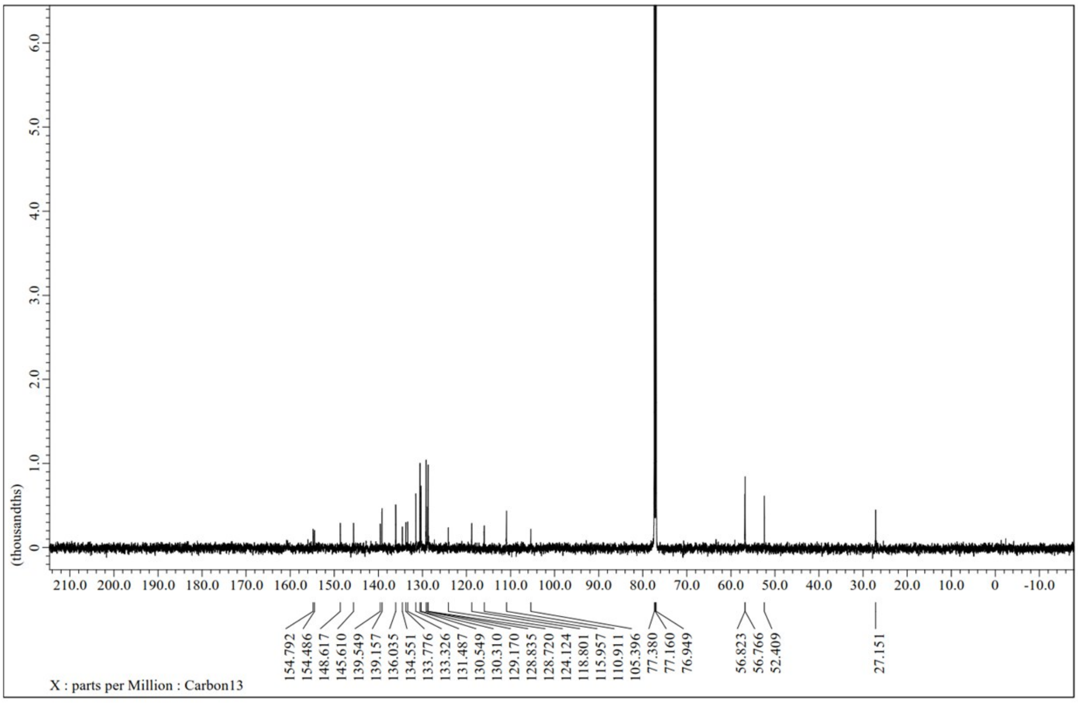
(thousandths)
X : parts per Million : Carbon13
154.792
154.486
148.617
145.610
139.549
139.157
136.035
134.551
133.776
133.326
131.487
130.549
130.310
129.170
128.835
128.720
124.124
118.801
115.957
110.911
105.396
77.380
77.160
76.949
56.823
56.766
52.409
27.151

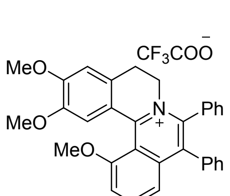
MeO
MeO
MeO
CF3COO
N
Ph
Ph

3ha

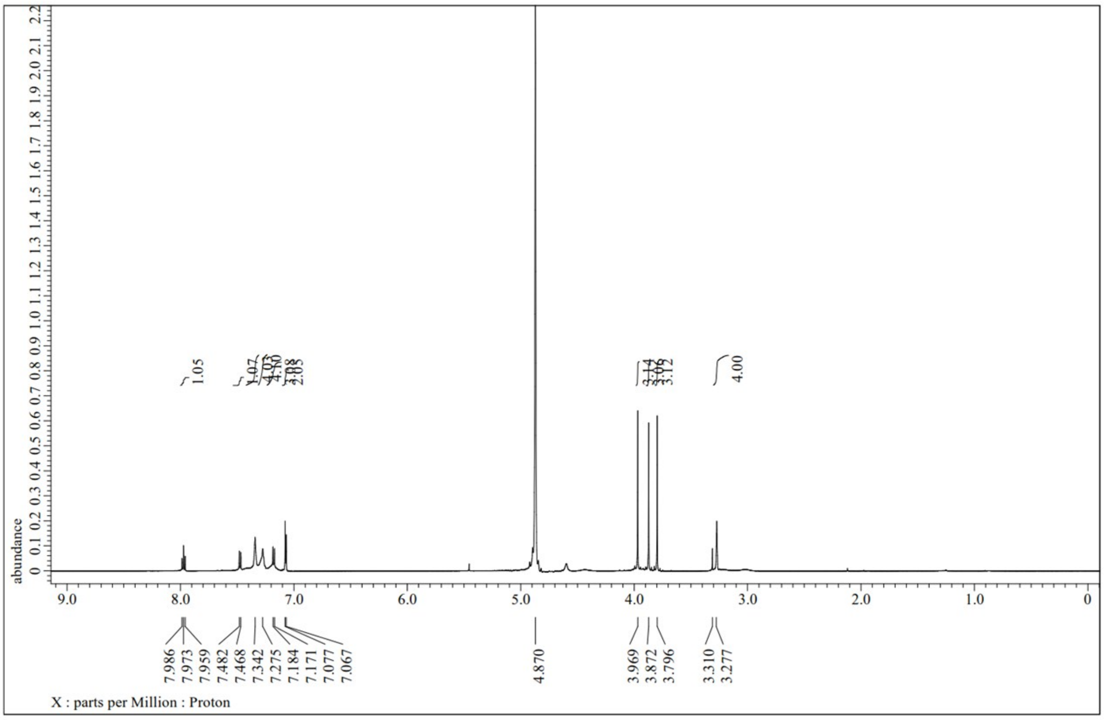
abundance
X : parts per Million : Proton

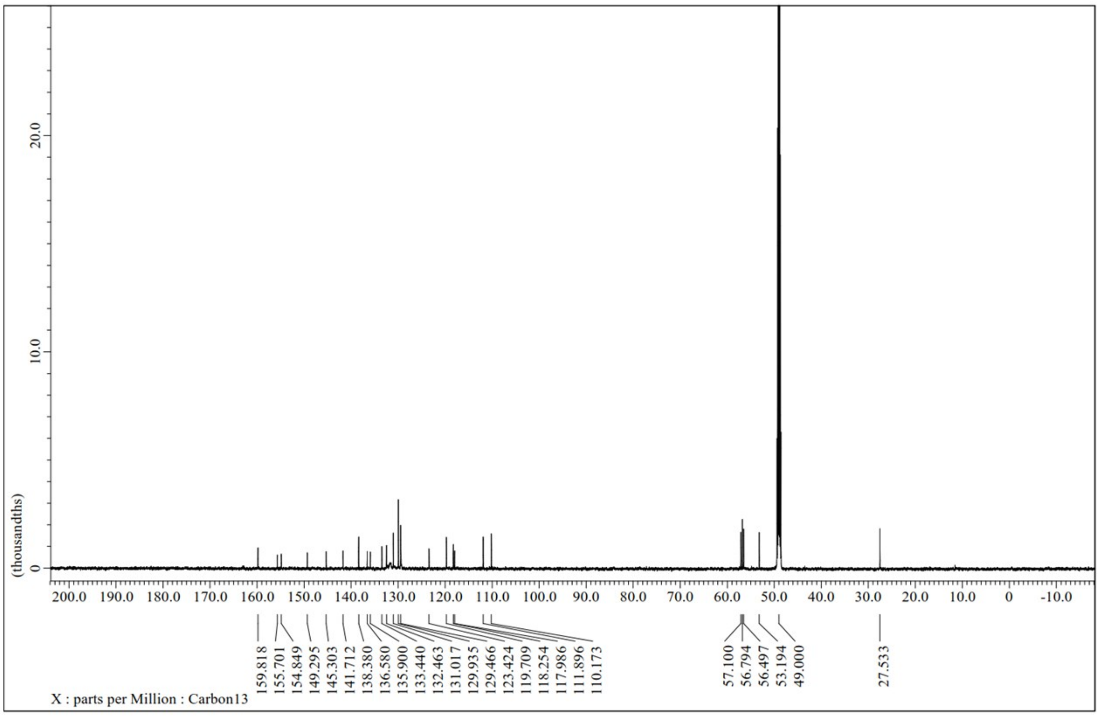
(thousandths)
X : parts per Million : Carbon13

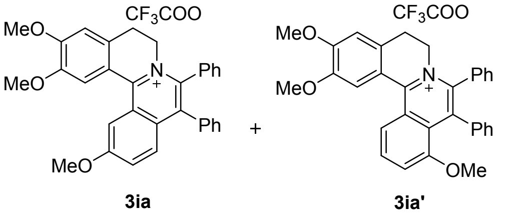
CF3COO
MeO
MeO
Ph
Ph
MeO
OMe

3ia + **3ia'**

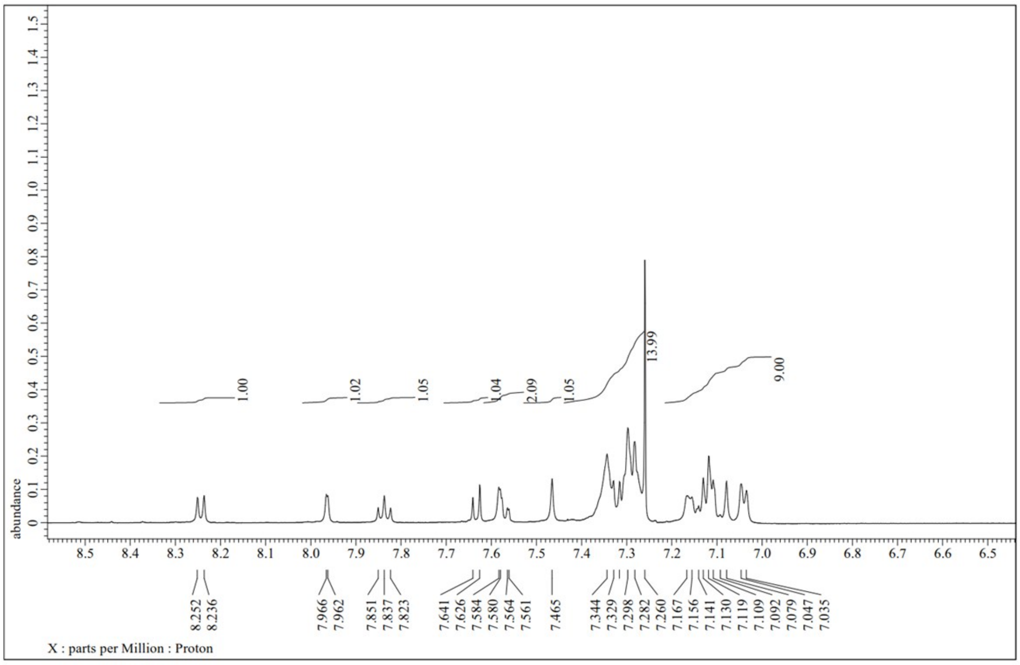
abundance
X : parts per Million : Proton

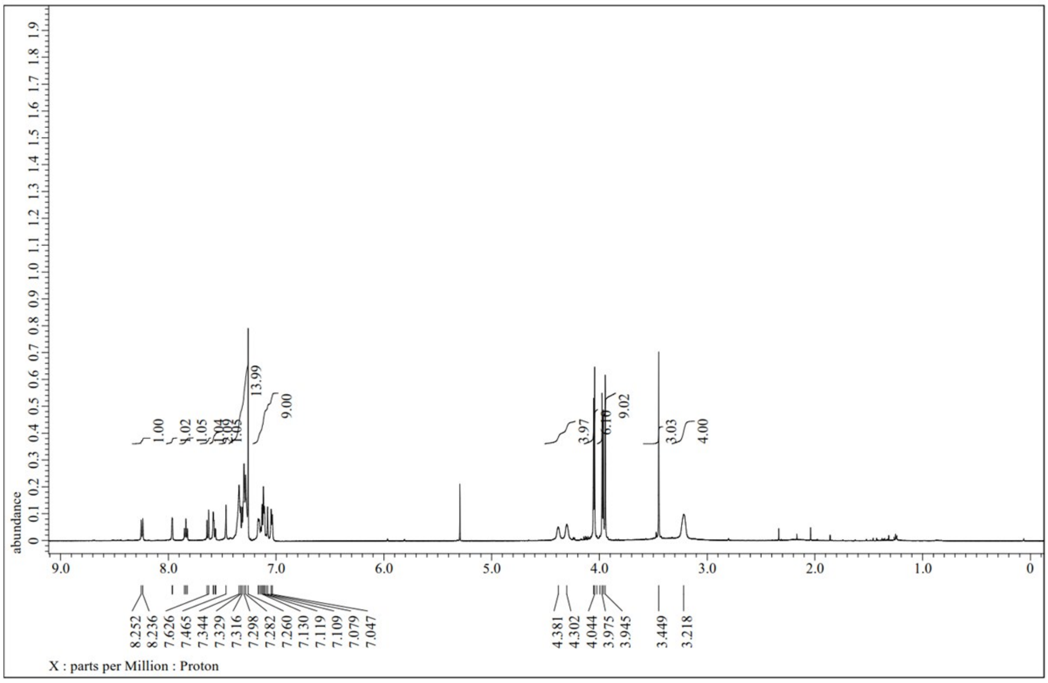
abundance
X : parts per Million : Proton

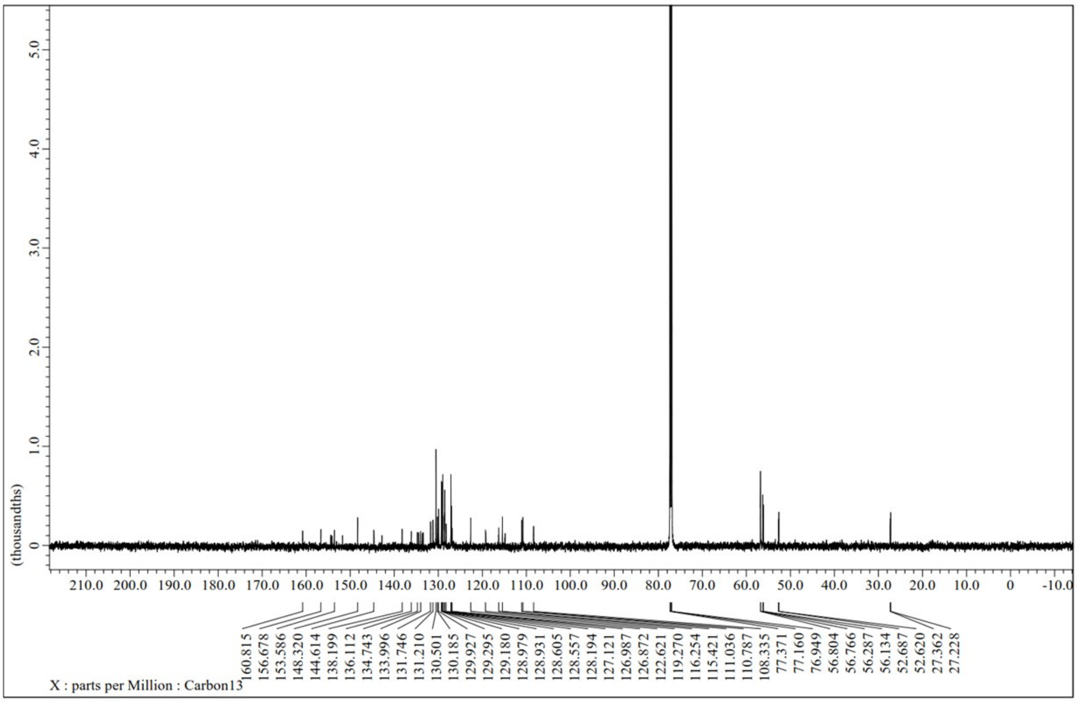
(thousandths)
X : parts per Million : Carbon13
160.815
156.678
153.586
148.320
144.614
138.199
136.112
134.743
133.996
131.746
131.210
130.501
130.185
129.927
129.295
129.180
128.979
128.931
128.605
128.557
128.194
127.121
126.987
126.872
122.621
119.270
116.254
115.421
111.036
110.787
108.335
77.371
77.160
76.949
56.804
56.766
56.287
56.134
52.687
52.620
27.362
27.228

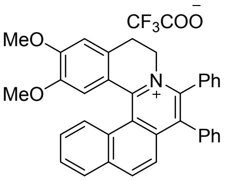
MeO
MeO
CF3COO
N
+
Ph
Ph

3ja

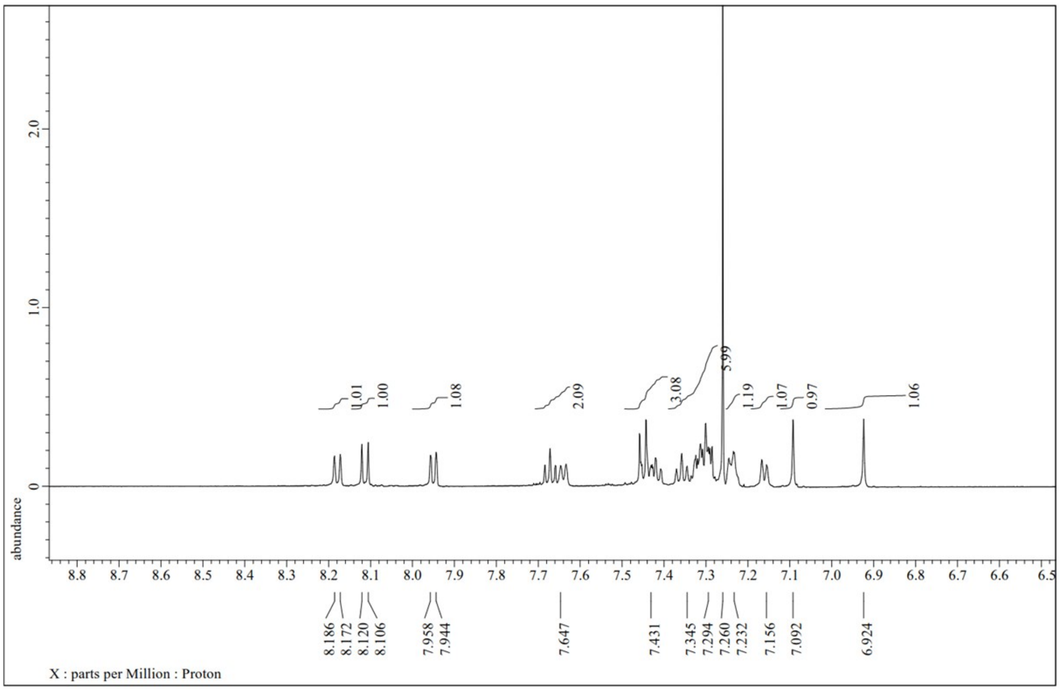
abundance
X : parts per Million : Proton
1.01
1.00
1.08
2.09
3.08
5.99
1.19
1.07
0.97
1.06
8.186
8.172
8.120
8.106
7.958
7.944
7.647
7.431
7.345
7.294
7.260
7.232
7.156
7.092
6.924

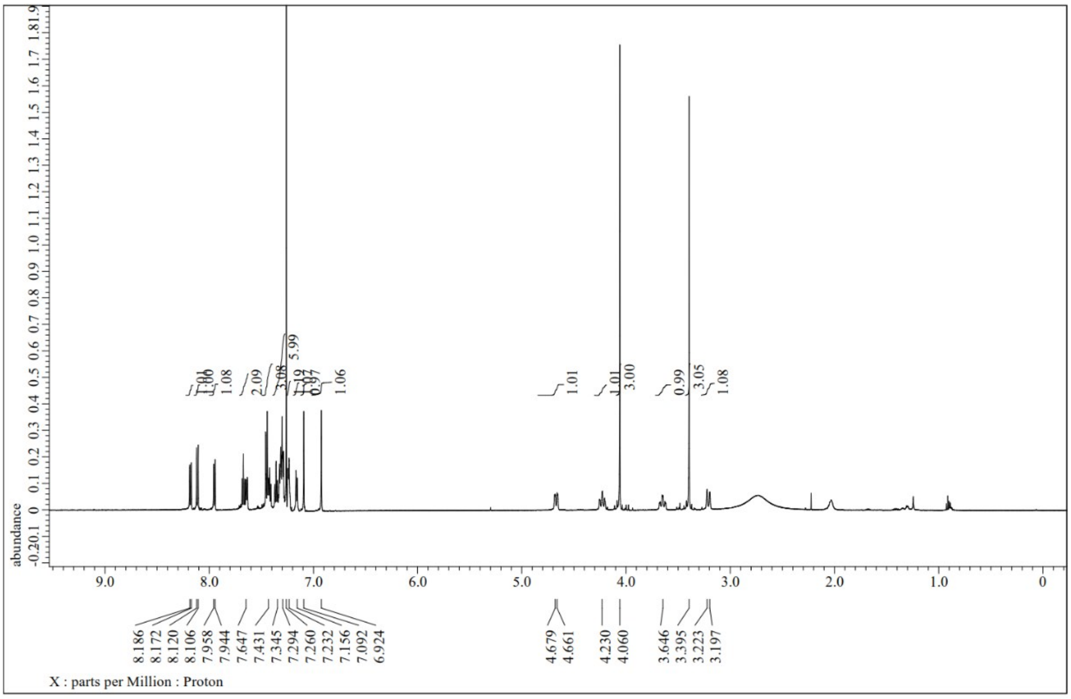
abundance
X : parts per Million : Proton
8.186
8.172
8.120
8.106
7.958
7.944
7.647
7.431
7.345
7.294
7.260
7.232
7.156
7.092
6.924
4.679
4.661
4.230
4.060
3.646
3.395
3.223
3.197

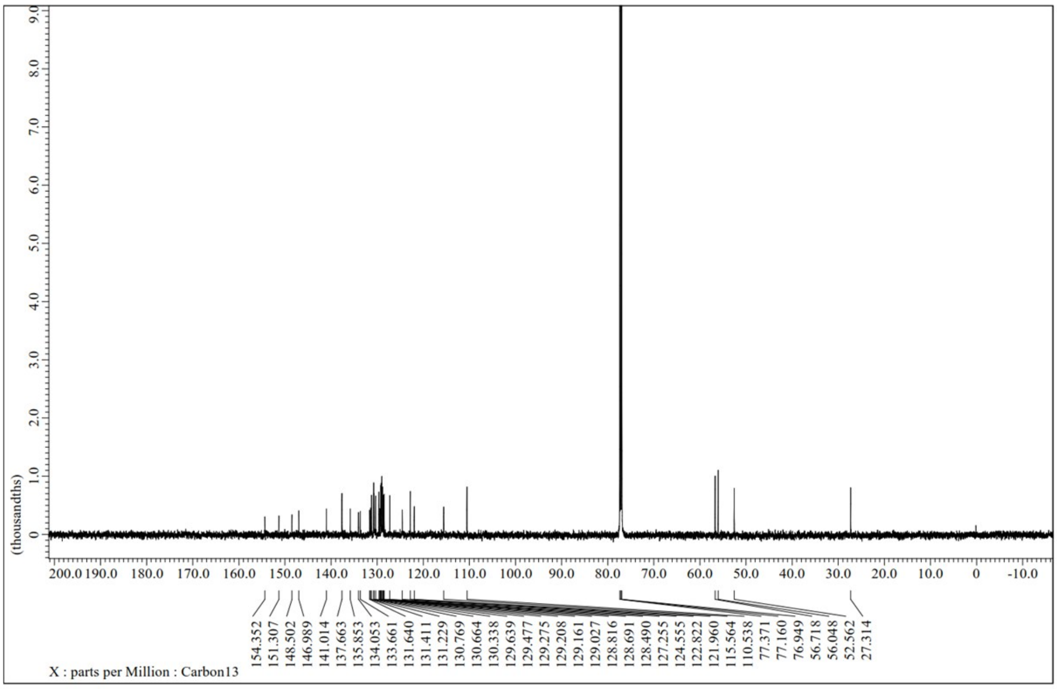
(thousandths)
X : parts per Million : Carbon13
154.352
151.307
148.502
146.989
141.014
137.663
135.853
134.053
133.661
131.640
131.411
131.229
130.769
130.664
130.338
129.639
129.477
129.275
129.208
129.161
129.027
128.816
128.691
128.490
127.255
124.555
122.822
121.960
115.564
110.538
77.371
77.160
76.949
56.718
56.048
52.562
27.314

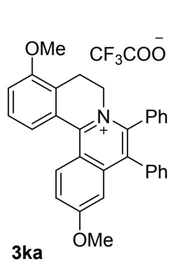
OMe
CF3COO
Ph
Ph
OMe

3ka

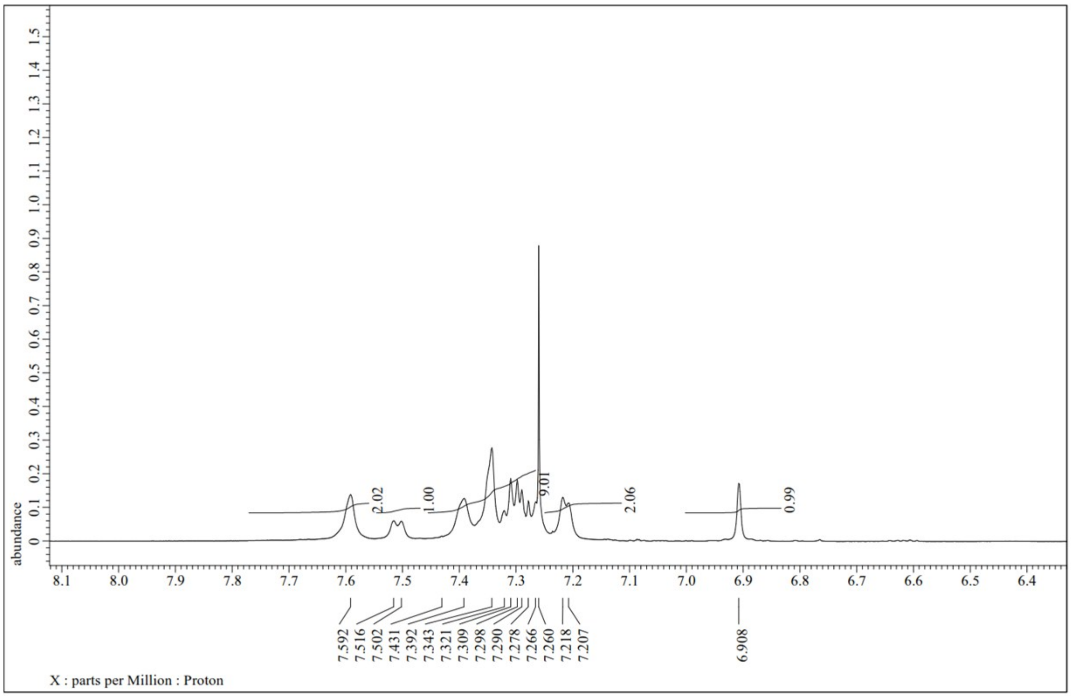
abundance
2.02
1.00
9.01
2.06
0.99
7.592
7.516
7.502
7.431
7.392
7.343
7.321
7.309
7.298
7.290
7.278
7.266
7.260
7.218
7.207
6.908
X : parts per Million : Proton

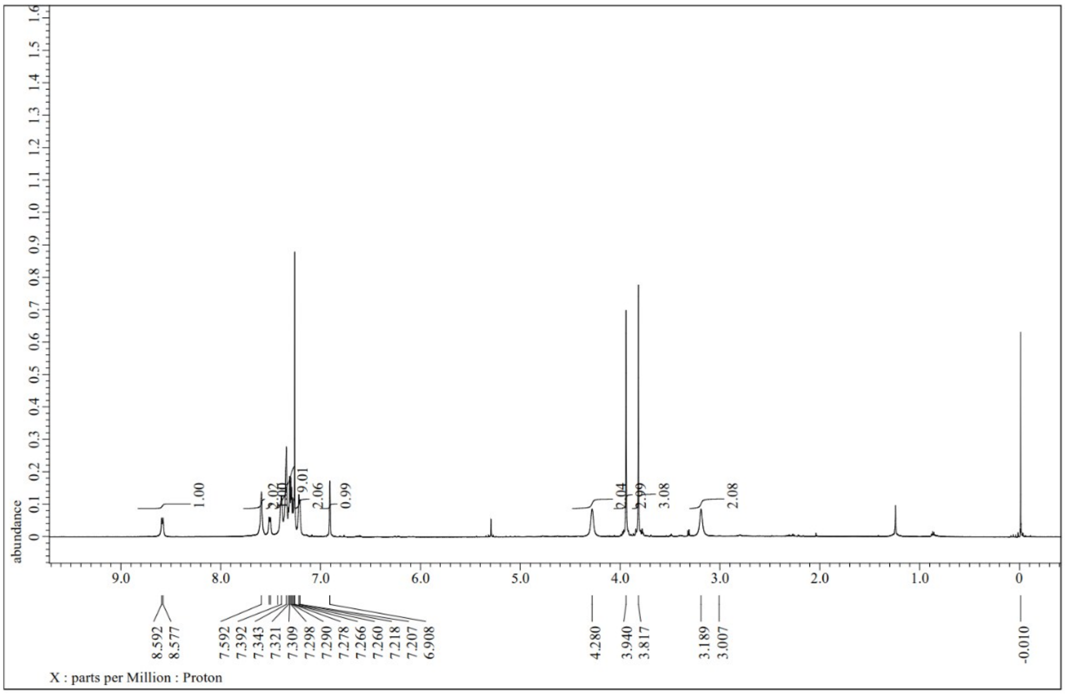
abundance
1.00
2.06
0.99
9.01
3.08
2.08
8.592
8.577
7.592
7.392
7.343
7.321
7.309
7.298
7.290
7.278
7.266
7.260
7.218
7.207
6.908
4.280
3.940
3.817
3.189
3.007
-0.010
X : parts per Million : Proton

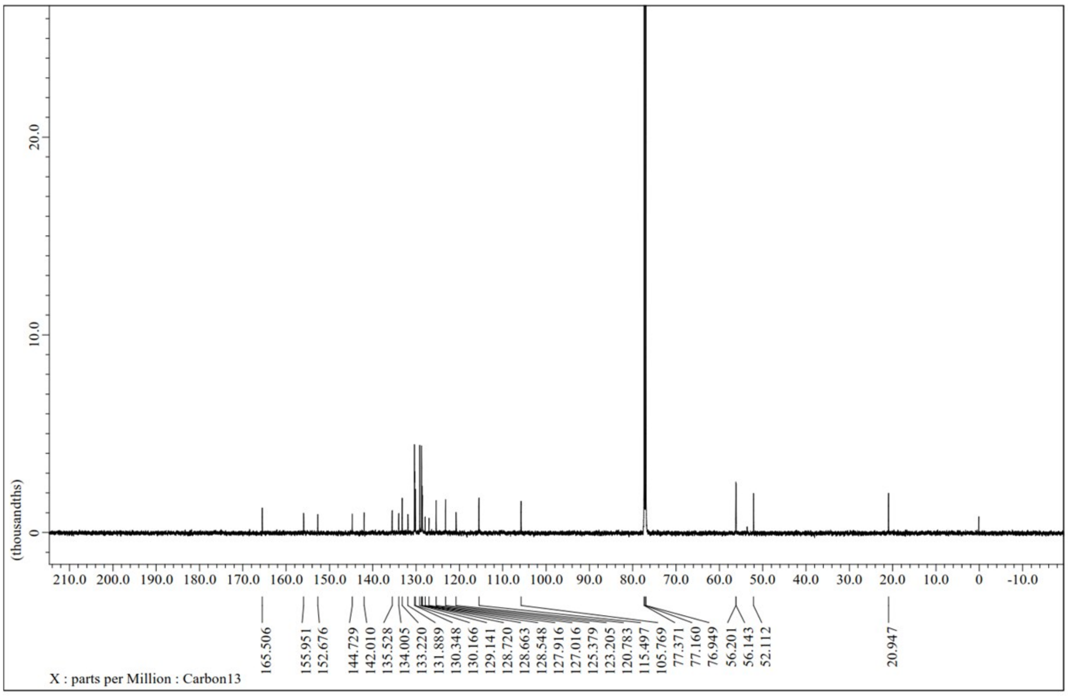
(thousandths)
0
10.0
20.0
210.0 200.0 190.0 180.0 170.0 160.0 150.0 140.0 130.0 120.0 110.0 100.0 90.0 80.0 70.0 60.0 50.0 40.0 30.0 20.0 10.0 0 -10.0
165.506
155.951
152.676
144.729
142.010
135.528
134.005
133.220
131.889
130.348
130.166
129.141
128.720
128.663
128.548
127.916
127.016
125.379
123.205
120.783
115.497
105.769
77.371
77.160
76.949
56.201
56.143
52.112
20.947
X : parts per Million : Carbon13

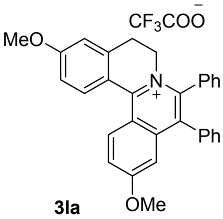
CF3COO
MeO
N
Ph
Ph
OMe

3la

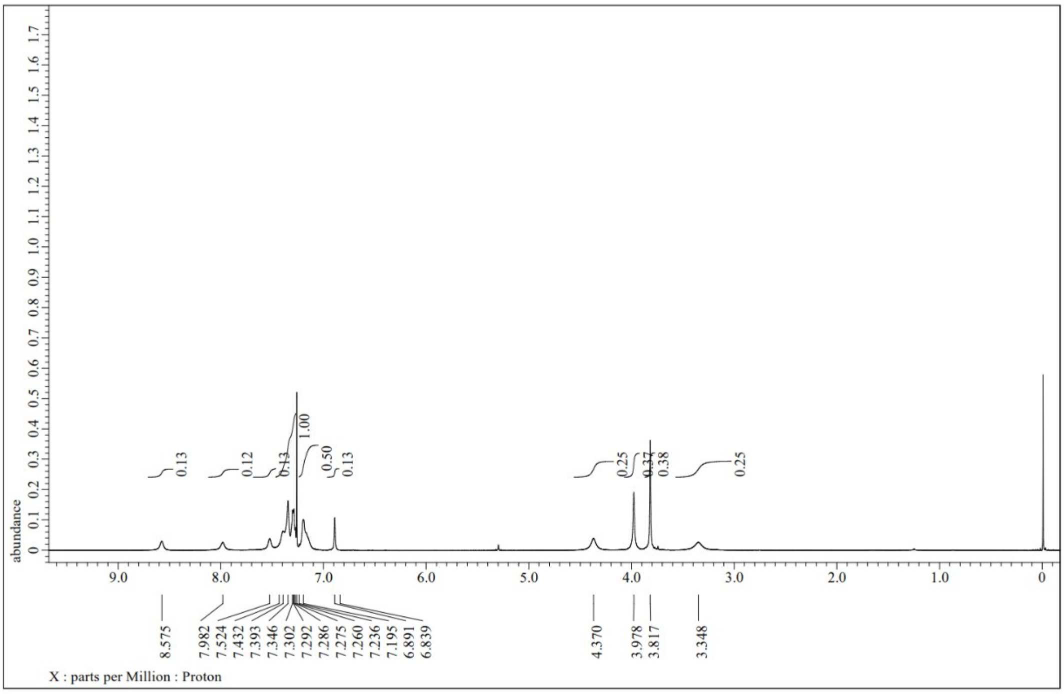
abundance
0 0.1 0.2 0.3 0.4 0.5 0.6 0.7 0.8 0.9 1.0 1.1 1.2 1.3 1.4 1.5 1.6 1.7
0.13
0.12
0.13
1.00
0.50
0.13
0.25
0.37
0.38
0.25
9.0 8.0 7.0 6.0 5.0 4.0 3.0 2.0 1.0 0
8.575
7.982
7.524
7.432
7.393
7.346
7.302
7.292
7.286
7.275
7.260
7.236
7.195
6.891
6.839
4.370
3.978
3.817
3.348
X : parts per Million : Proton

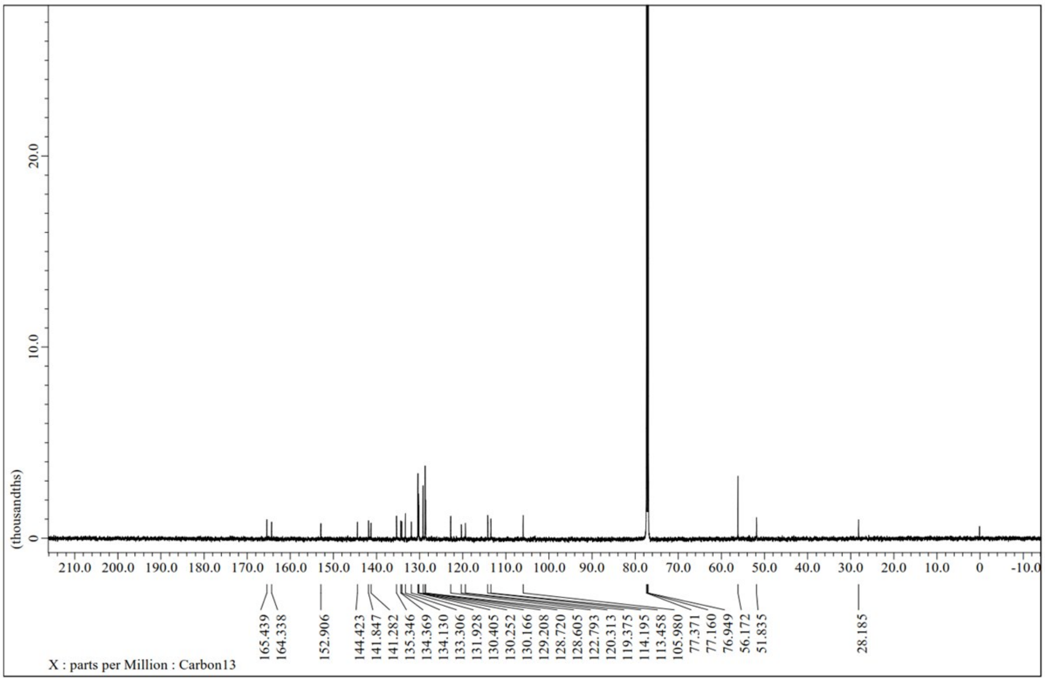
(thousandths)
165.439
164.338
152.906
144.423
141.847
141.282
135.346
134.369
134.130
133.306
131.928
130.405
130.252
130.166
129.208
128.720
128.605
122.793
120.313
119.375
114.195
113.458
105.980
77.371
77.160
76.949
56.172
51.835
28.185
X : parts per Million : Carbon13

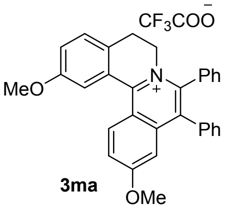
CF_3COO^-
MeO
N
+
Ph
Ph
OMe

3ma

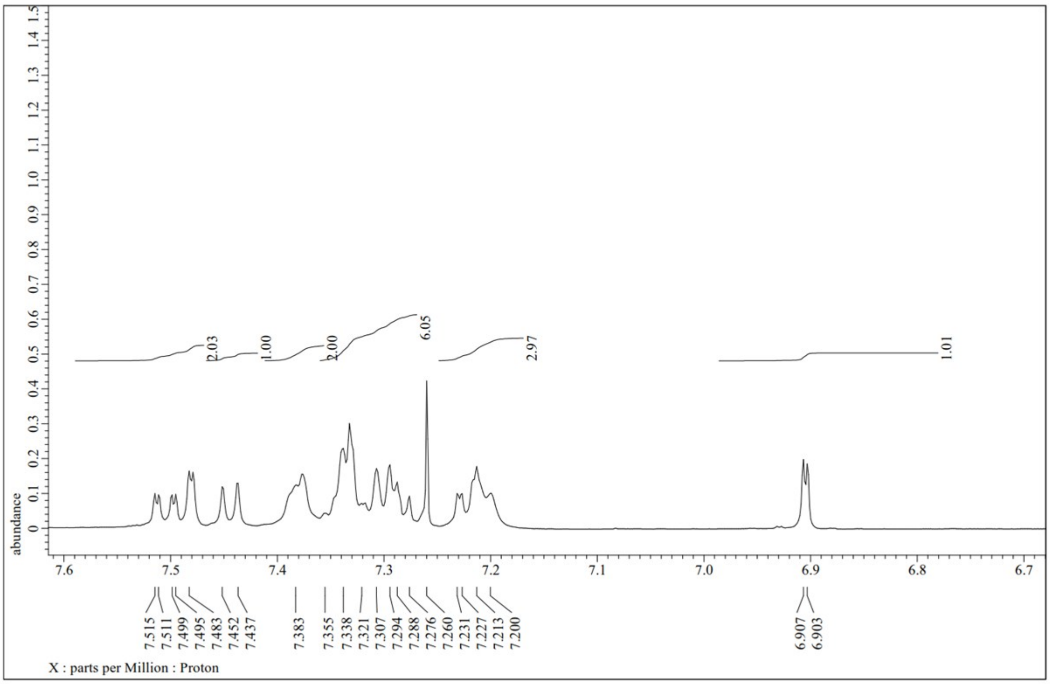
abundance
2.03
1.00
2.00
6.05
2.97
1.01
7.515
7.511
7.499
7.495
7.483
7.452
7.437
7.383
7.355
7.338
7.321
7.307
7.294
7.288
7.276
7.260
7.231
7.227
7.213
7.200
6.907
6.903
X : parts per Million : Proton

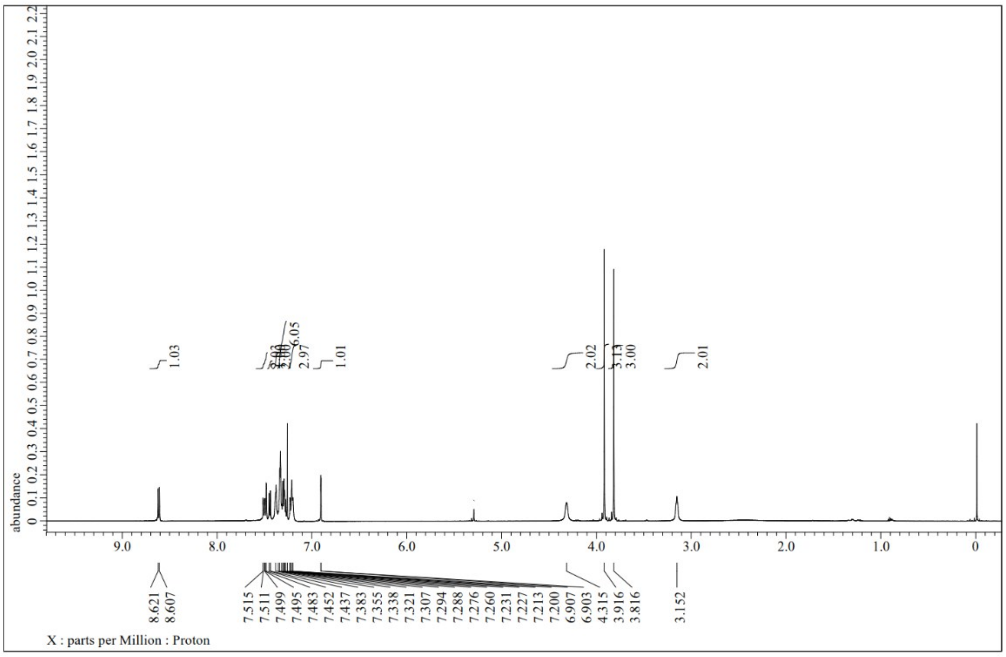
abundance
X : parts per Million : Proton

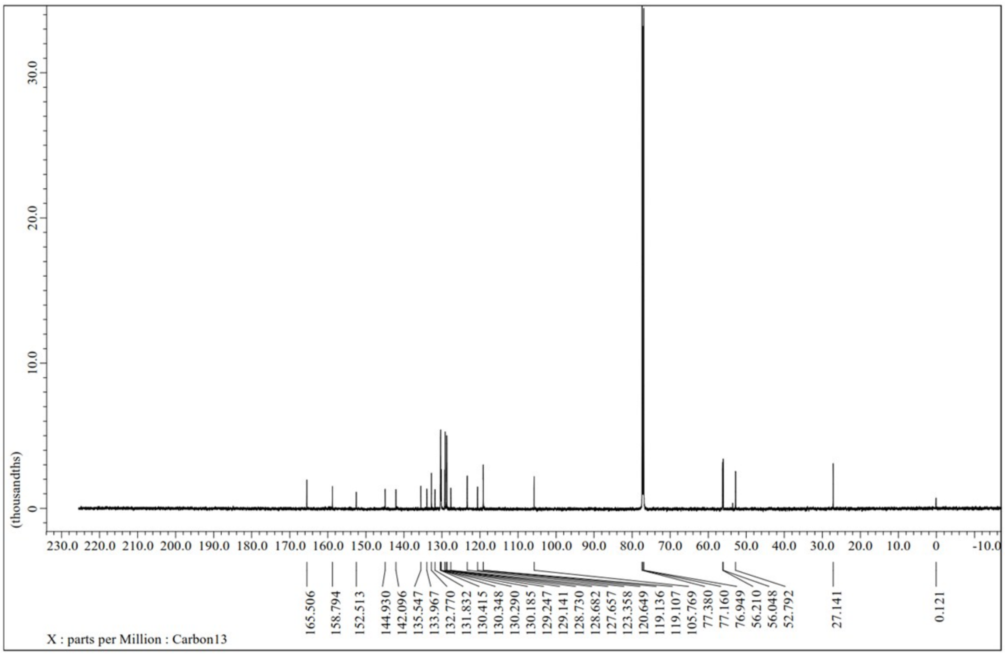
(thousandths)
X : parts per Million : Carbon13

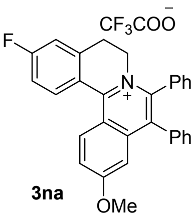
F
CF3COO
N
Ph
Ph
OMe

3na

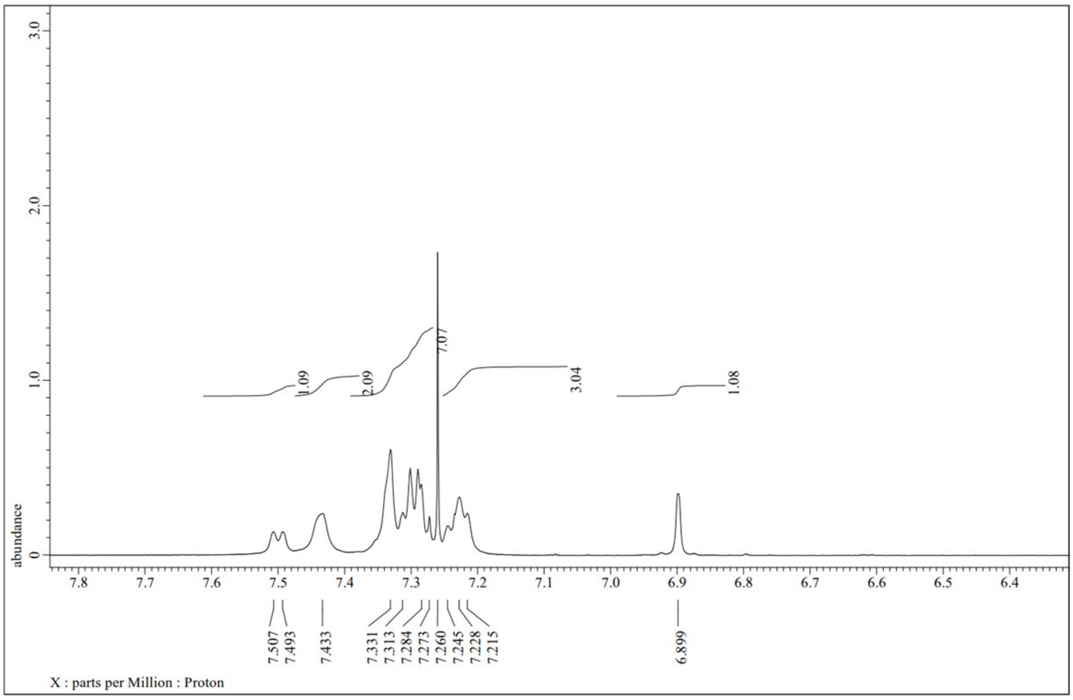
abundance
X : parts per Million : Proton
1.09
2.09
7.07
3.04
1.08
7.507
7.493
7.433
7.331
7.313
7.284
7.273
7.260
7.245
7.228
7.215
6.899

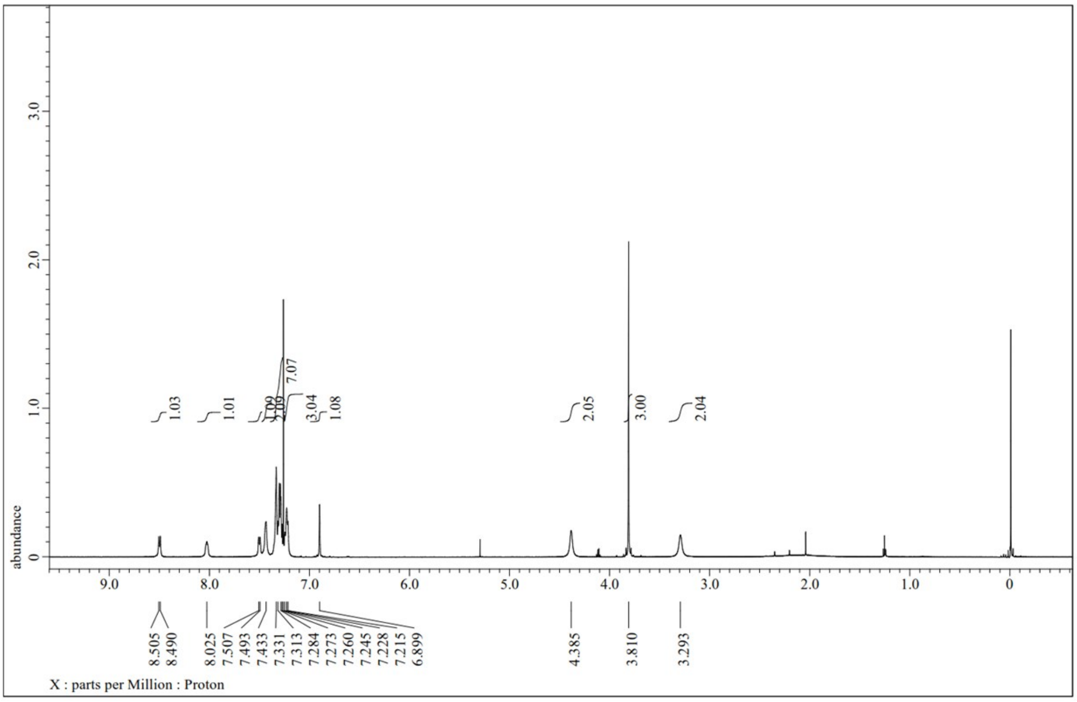
abundance
X : parts per Million : Proton
1.03
1.01
1.09
2.09
7.07
3.04
1.08
2.05
3.00
2.04
8.505
8.490
8.025
7.507
7.493
7.433
7.331
7.313
7.284
7.273
7.260
7.245
7.228
7.215
6.899
4.385
3.810
3.293

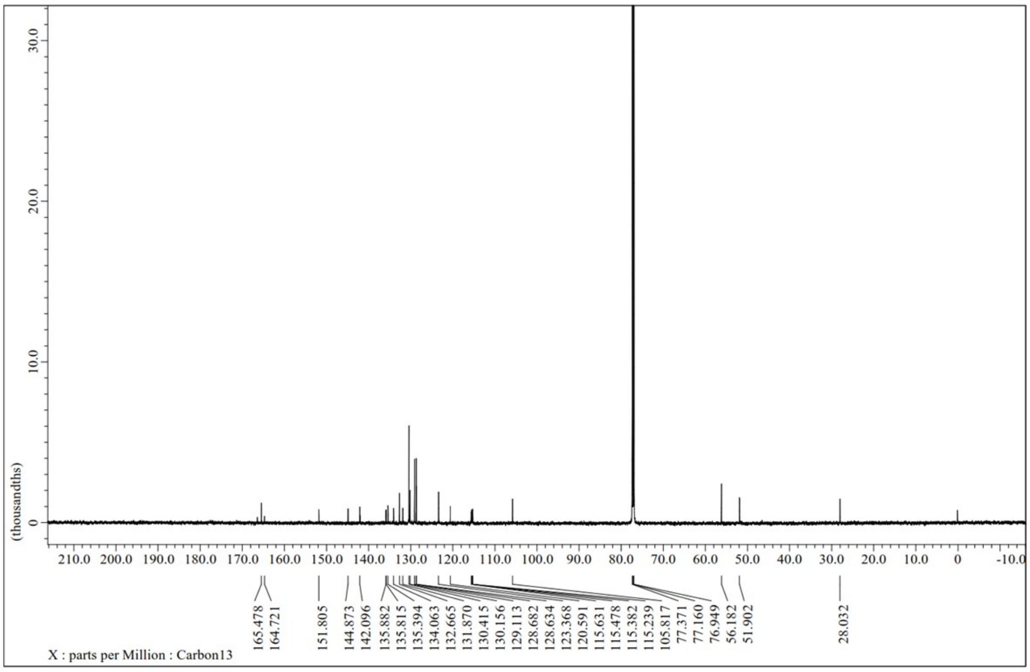

30.0
20.0
10.0
(thousandths)
0
210.0 200.0 190.0 180.0 170.0 160.0 150.0 140.0 130.0 120.0 110.0 100.0 90.0 80.0 70.0 60.0 50.0 40.0 30.0 20.0 10.0 0 -10.0
165.478
164.721
151.805
144.873
142.096
135.882
135.815
135.394
134.063
132.665
131.870
130.415
130.156
129.113
128.682
128.634
123.368
120.591
115.631
115.478
115.382
115.239
105.817
77.371
77.160
76.949
56.182
51.902
28.032
X : parts per Million : Carbon13

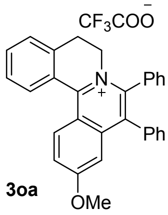

CF_3COO^-
N
+
Ph
Ph
OMe

3oa

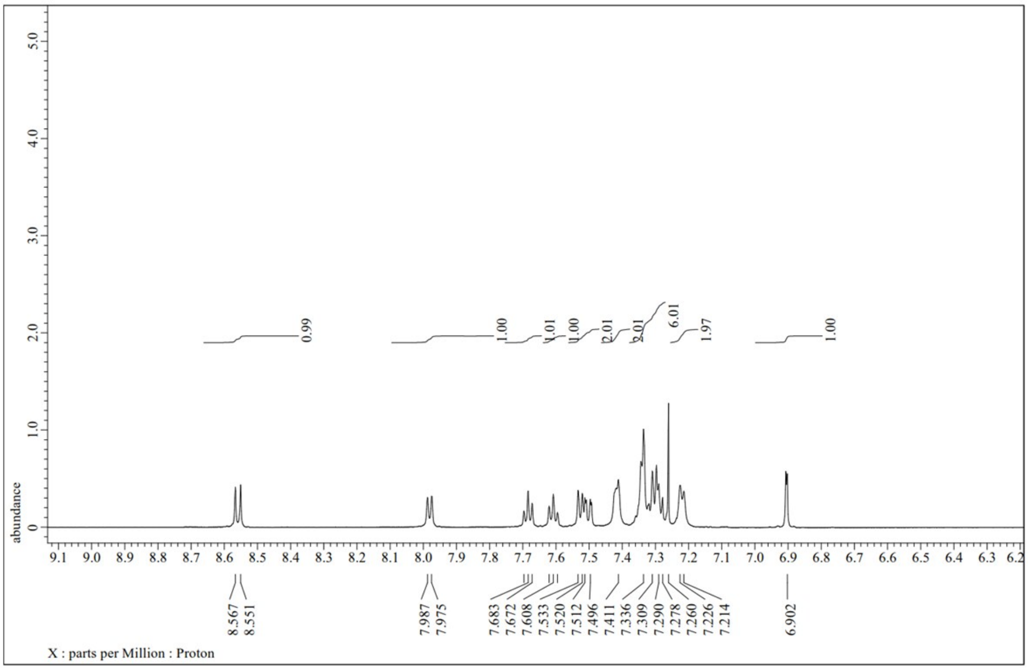

5.0
4.0
3.0
2.0
1.0
0
abundance
0.99
1.00
1.01
1.00
2.01
2.01
6.01
1.97
1.00
9.1 9.0 8.9 8.8 8.7 8.6 8.5 8.4 8.3 8.2 8.1 8.0 7.9 7.8 7.7 7.6 7.5 7.4 7.3 7.2 7.1 7.0 6.9 6.8 6.7 6.6 6.5 6.4 6.3 6.2
8.567
8.551
7.987
7.975
7.683
7.672
7.608
7.533
7.520
7.512
7.496
7.411
7.336
7.309
7.290
7.278
7.260
7.226
7.214
6.902
X : parts per Million : Proton

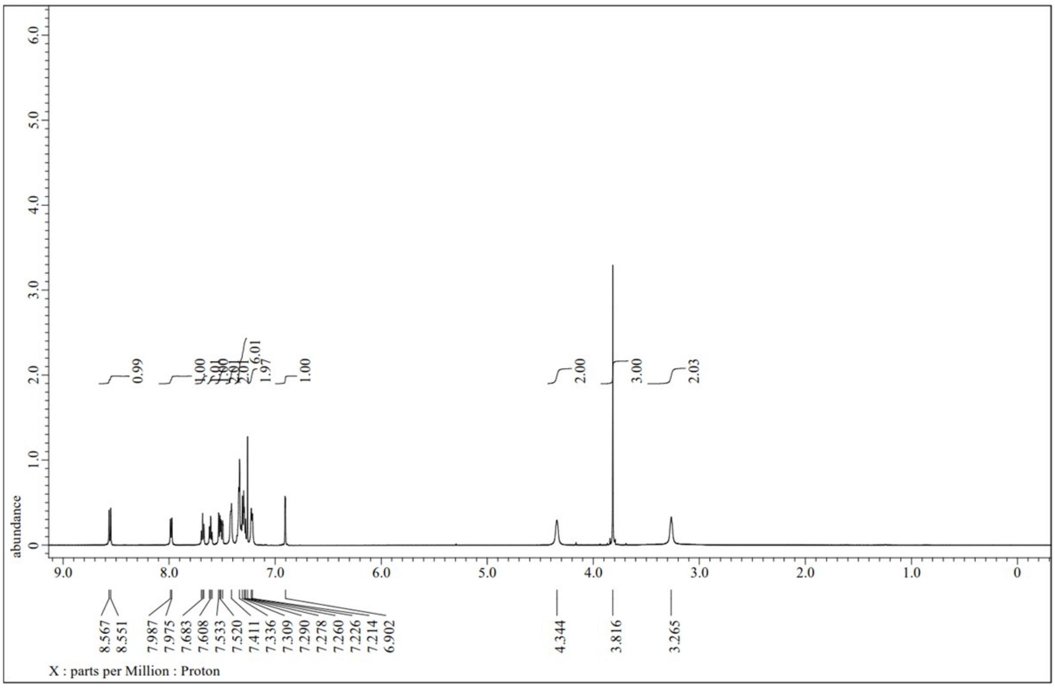
abundance
X : parts per Million : Proton
8.567
8.551
7.987
7.975
7.683
7.608
7.533
7.520
7.411
7.336
7.309
7.290
7.278
7.260
7.226
7.214
6.902
4.344
3.816
3.265
0.99
1.00
6.01
1.97
1.00
2.00
3.00
2.03

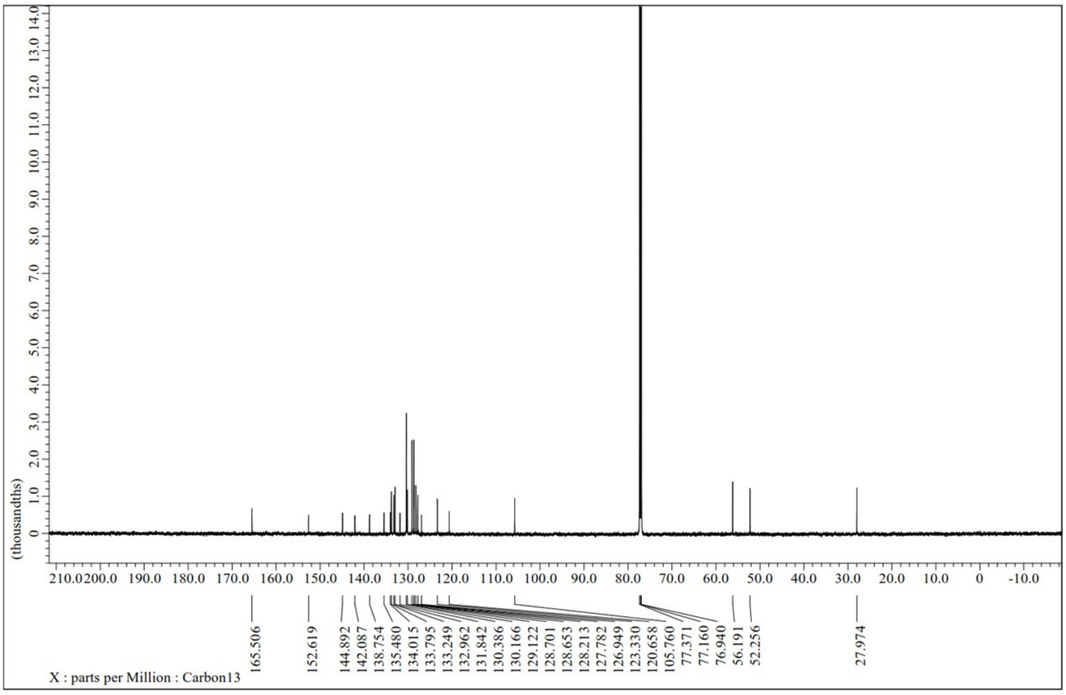
(thousandths)
X : parts per Million : Carbon13
165.506
152.619
144.892
142.087
138.754
135.480
134.015
133.795
133.249
132.962
131.842
130.386
130.166
129.122
128.701
128.653
128.213
127.782
126.949
123.330
120.658
105.760
77.371
77.160
76.940
56.191
52.256
27.974

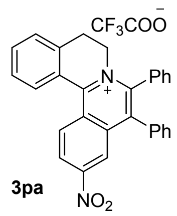

3pa

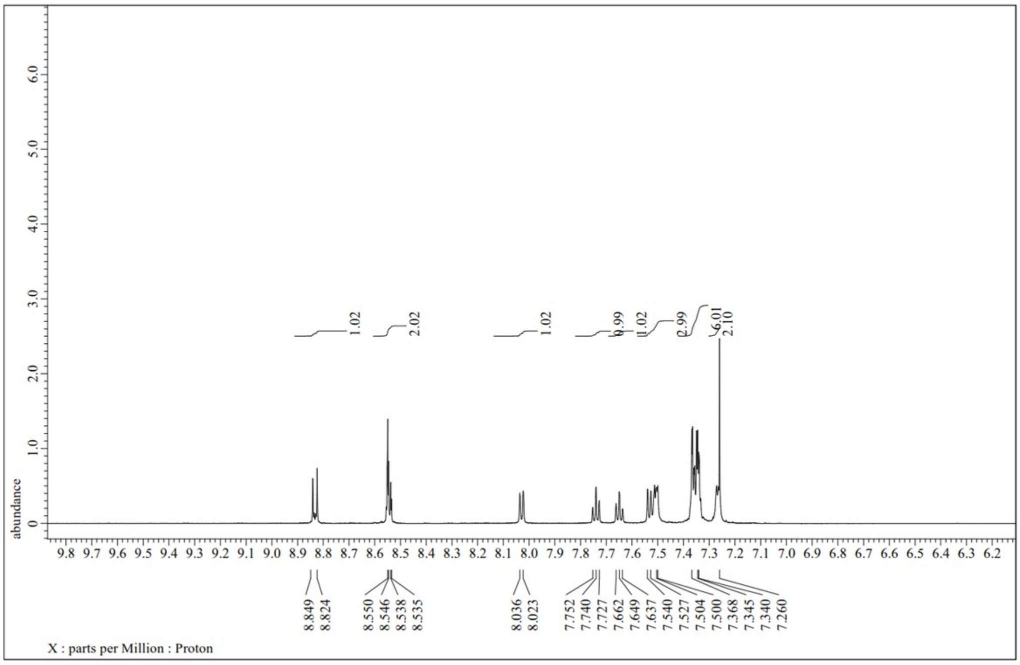

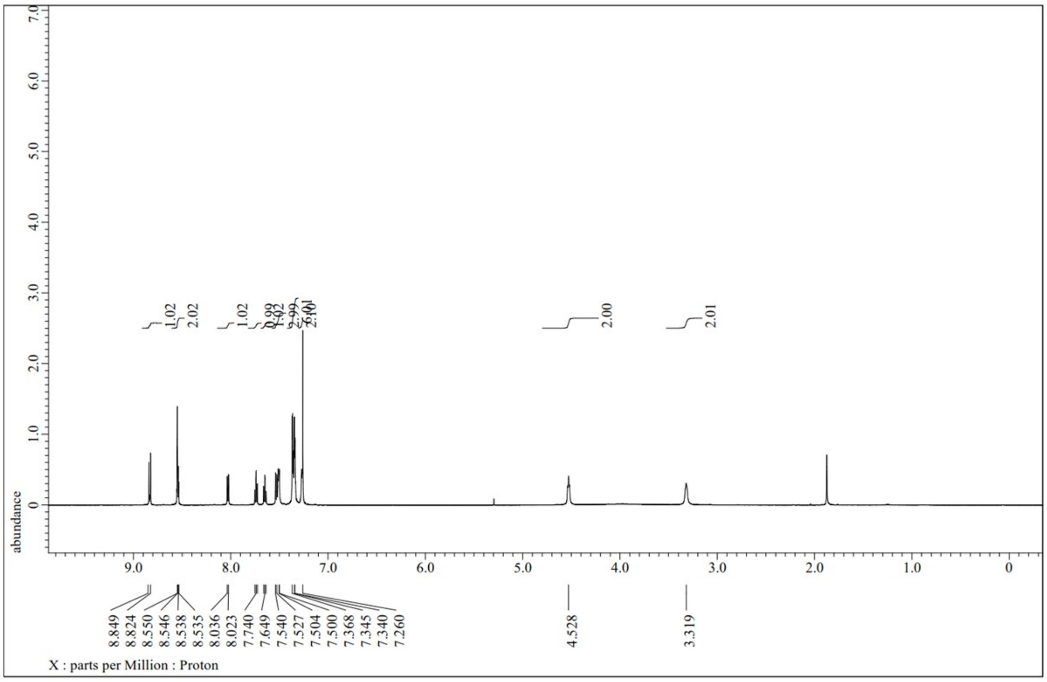

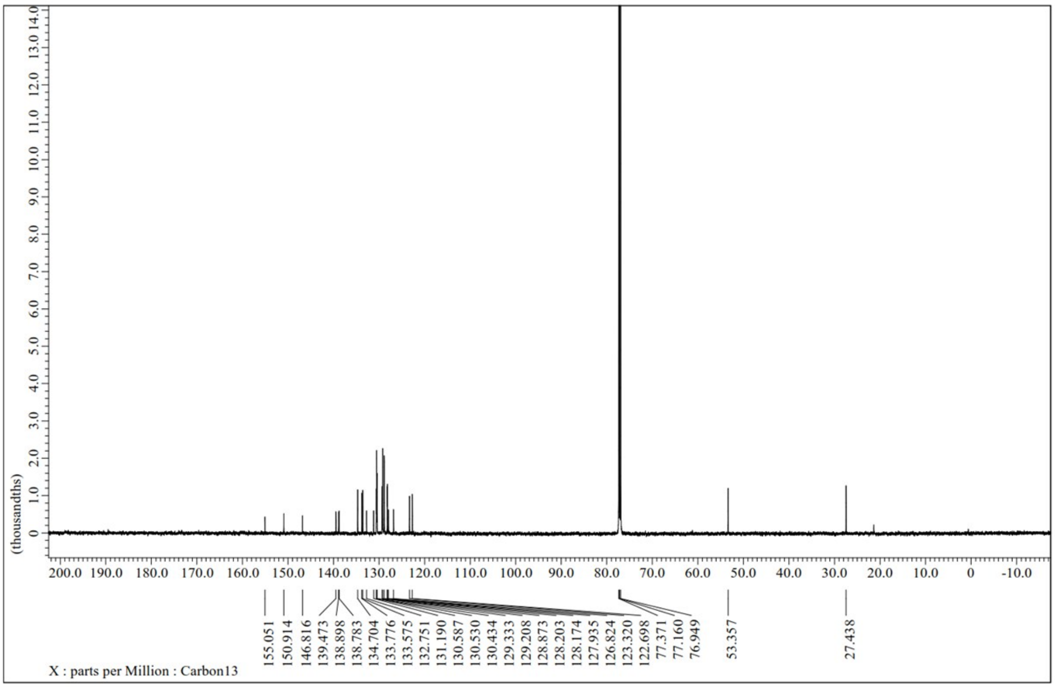

(thousandths)
X : parts per Million : Carbon13
155.051
150.914
146.816
139.473
138.898
138.783
134.704
133.776
133.575
132.751
131.190
130.587
130.530
130.434
129.333
129.208
128.873
128.203
128.174
127.935
126.824
123.320
122.698
77.371
77.160
76.949
53.357
27.438

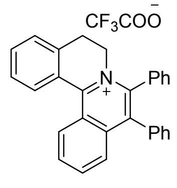

CF_3COO^-
N
+
Ph
Ph

3qa

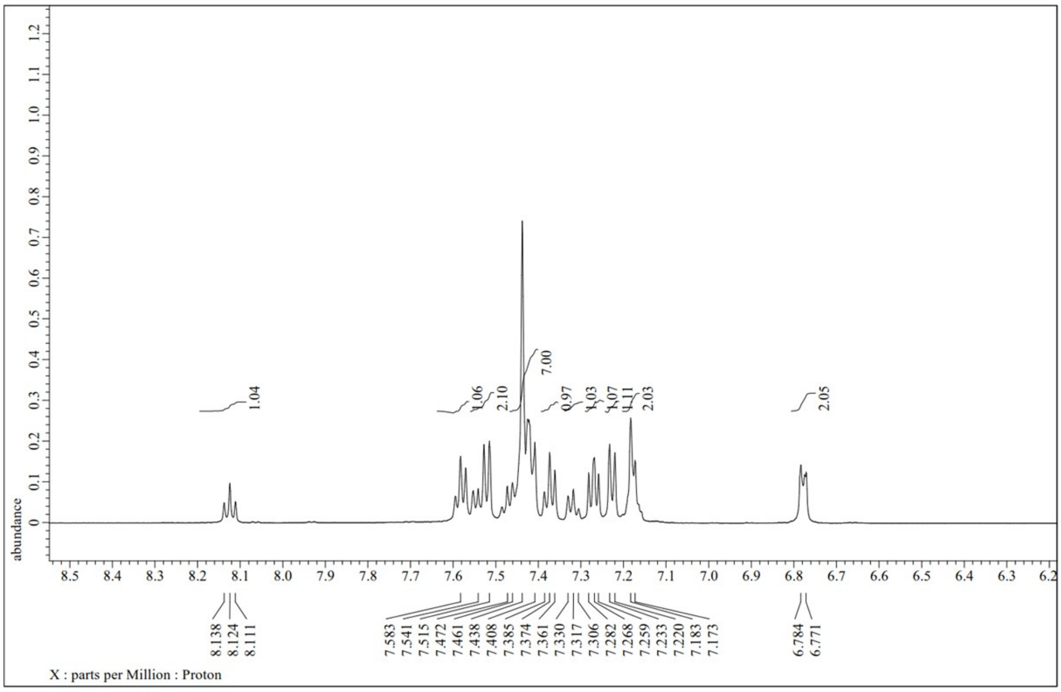

abundance
X : parts per Million : Proton
1.04
1.06
2.10
7.00
0.97
1.03
1.07
1.11
2.03
2.05
8.138
8.124
8.111
7.583
7.541
7.515
7.472
7.461
7.438
7.408
7.385
7.374
7.361
7.330
7.317
7.306
7.282
7.268
7.259
7.233
7.220
7.183
7.173
6.784
6.771

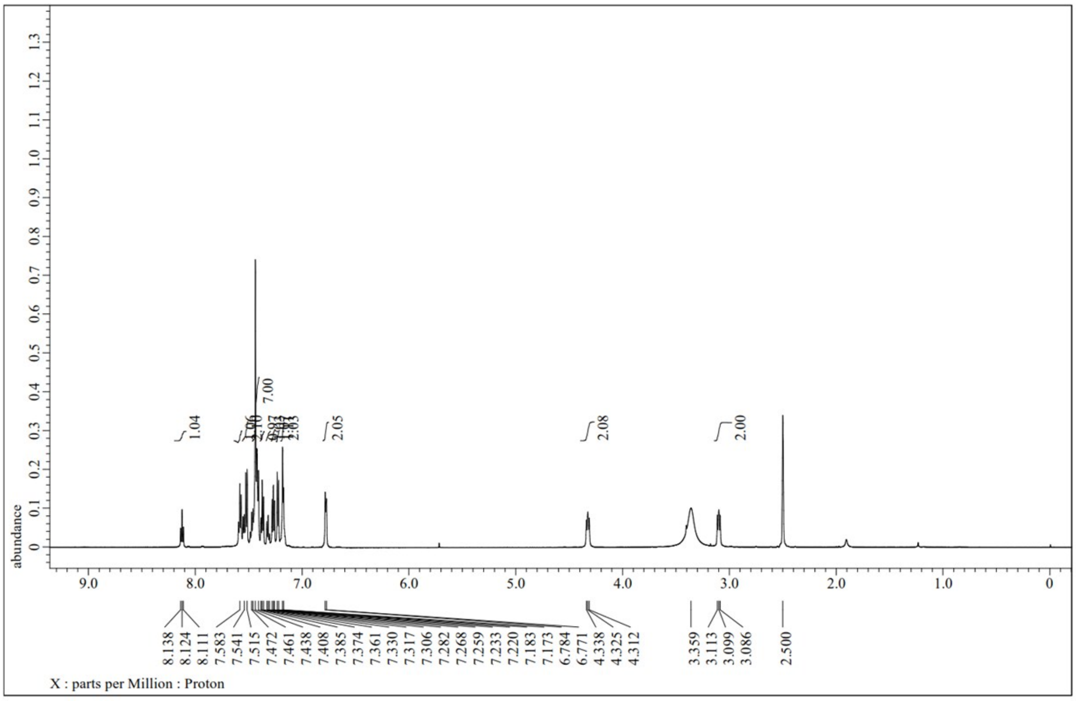
abundance
1.04
1.06
1.10
7.00
2.07
1.97
1.02
2.03
2.05
2.08
2.00
8.138 8.124 8.111 7.583 7.541 7.515 7.472 7.461 7.438 7.408 7.385 7.374 7.361 7.330 7.317 7.306 7.282 7.268 7.259 7.233 7.220 7.183 7.173 6.784 6.771 4.338 4.325 4.312
3.359 3.113 3.099 3.086
2.500
X : parts per Million : Proton

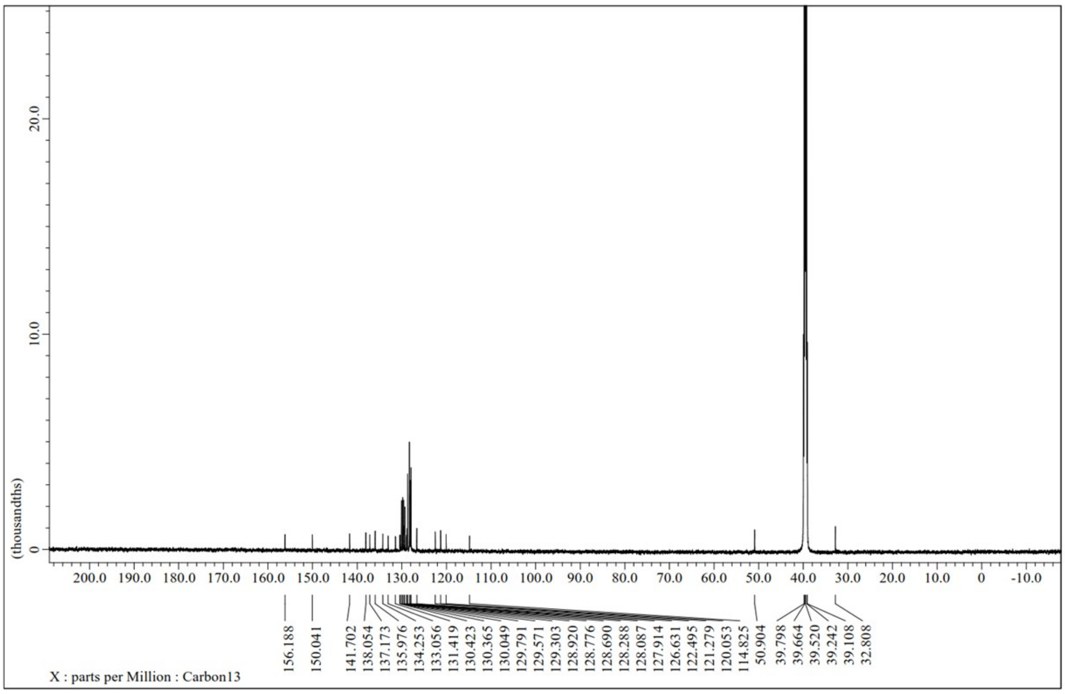
(thousandths)
156.188 150.041 141.702 138.054 137.173 135.976 134.253 133.056 131.419 130.423 130.365 130.049 129.791 129.571 129.303 128.920 128.776 128.690 128.288 128.087 127.914 126.631 122.495 121.279 120.053 114.825
50.904 39.798 39.664 39.520 39.242 39.108 32.808
X : parts per Million : Carbon13

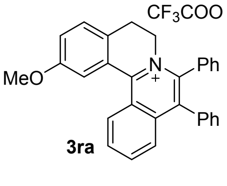
CF_3COO^-
MeO
N
+
Ph
Ph

3ra

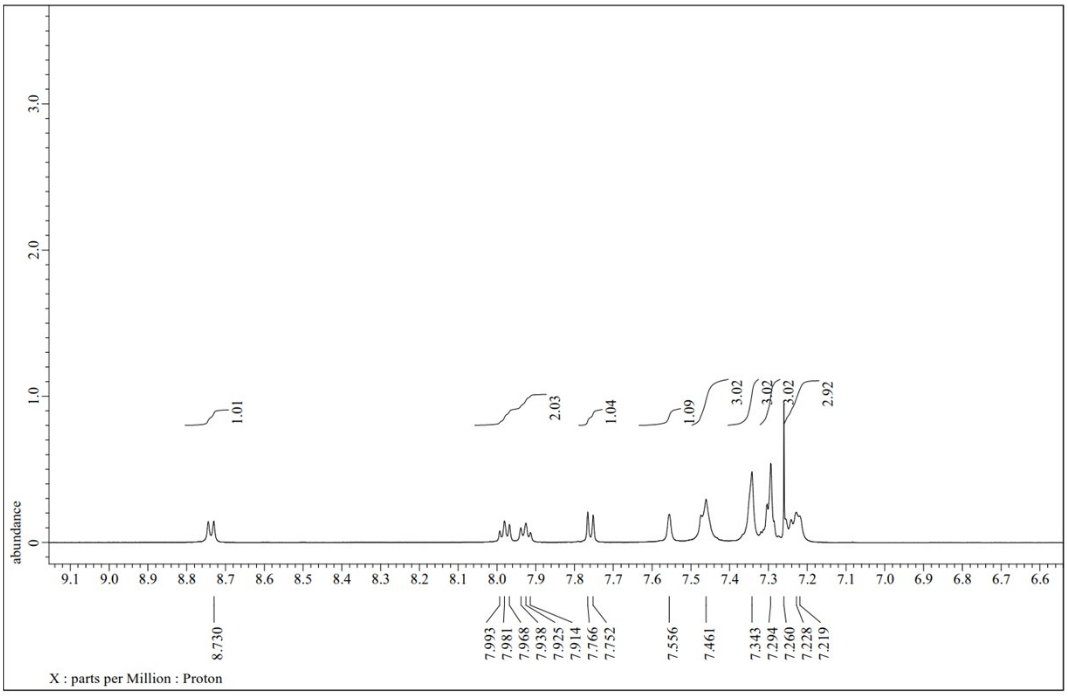
abundance
X : parts per Million : Proton
8.730
7.993
7.981
7.968
7.938
7.925
7.914
7.766
7.752
7.556
7.461
7.343
7.294
7.260
7.228
7.219
1.01
2.03
1.04
1.09
3.02
3.02
3.02
2.92

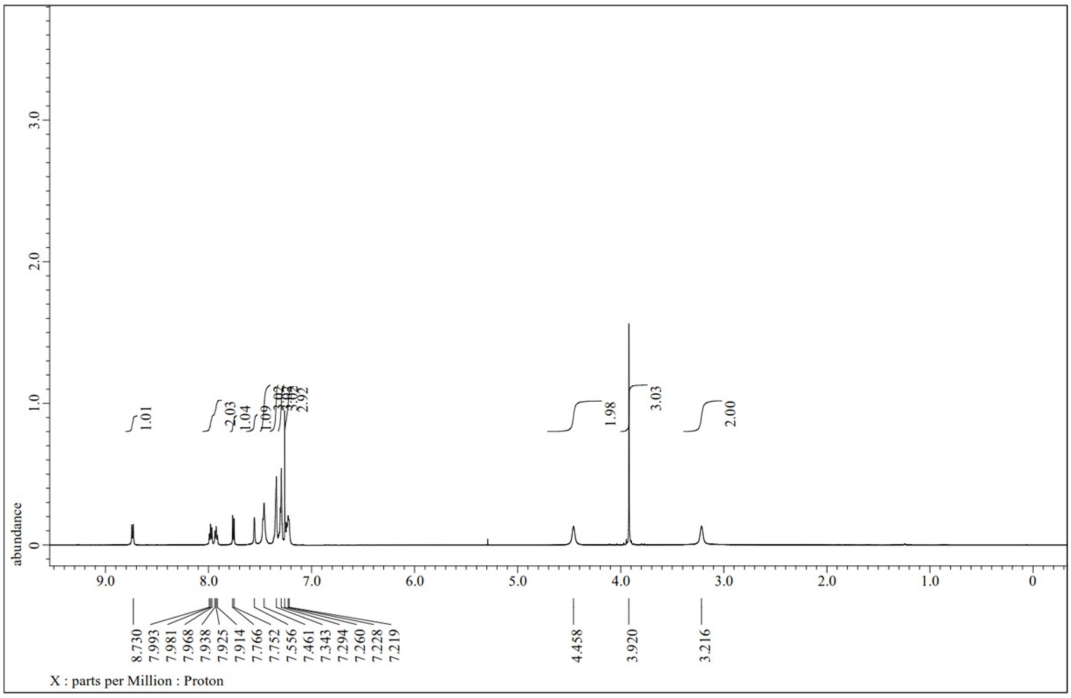
abundance
X : parts per Million : Proton
8.730
7.993
7.981
7.968
7.938
7.925
7.914
7.766
7.752
7.556
7.461
7.343
7.294
7.260
7.228
7.219
4.458
3.920
3.216
1.01
2.03
1.04
1.09
1.98
3.03
2.00

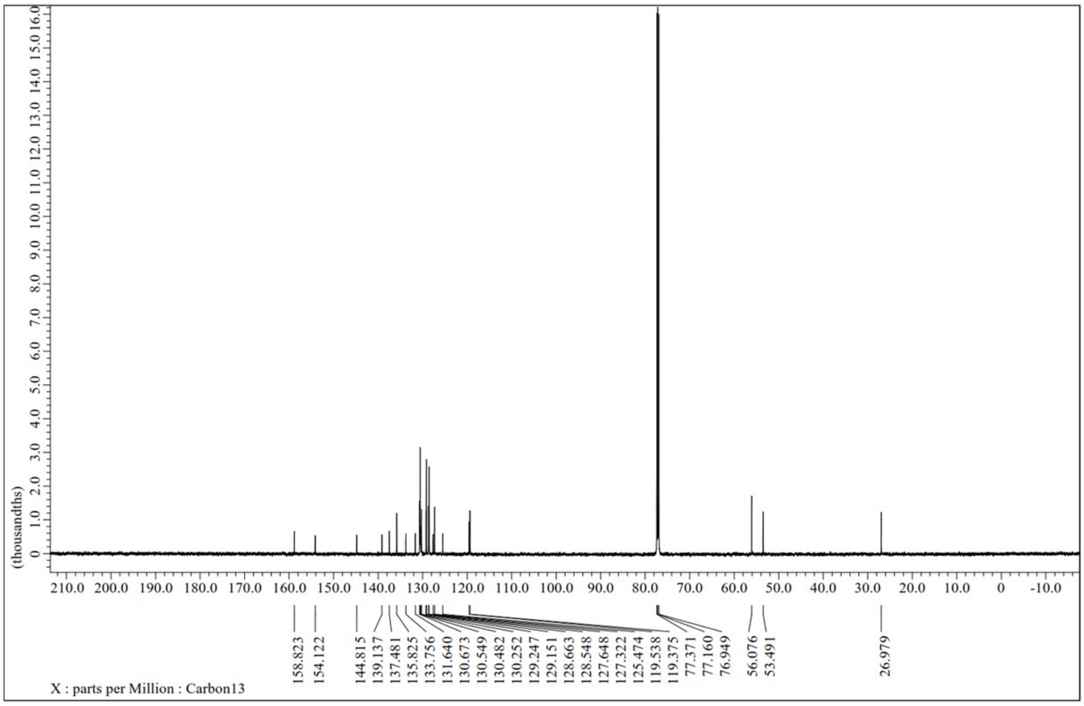

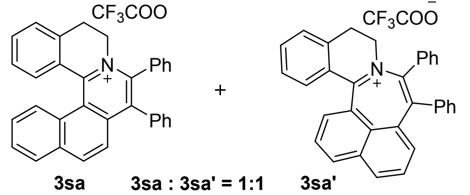

3sa **3sa : 3sa' = 1:1** **3sa'**

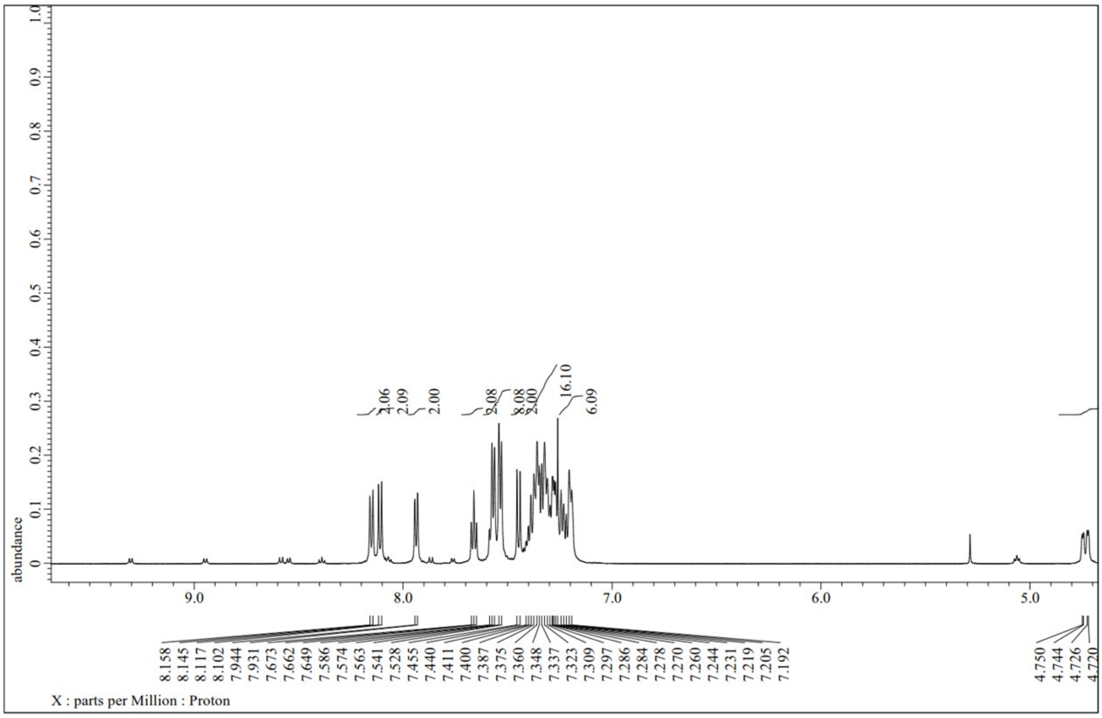

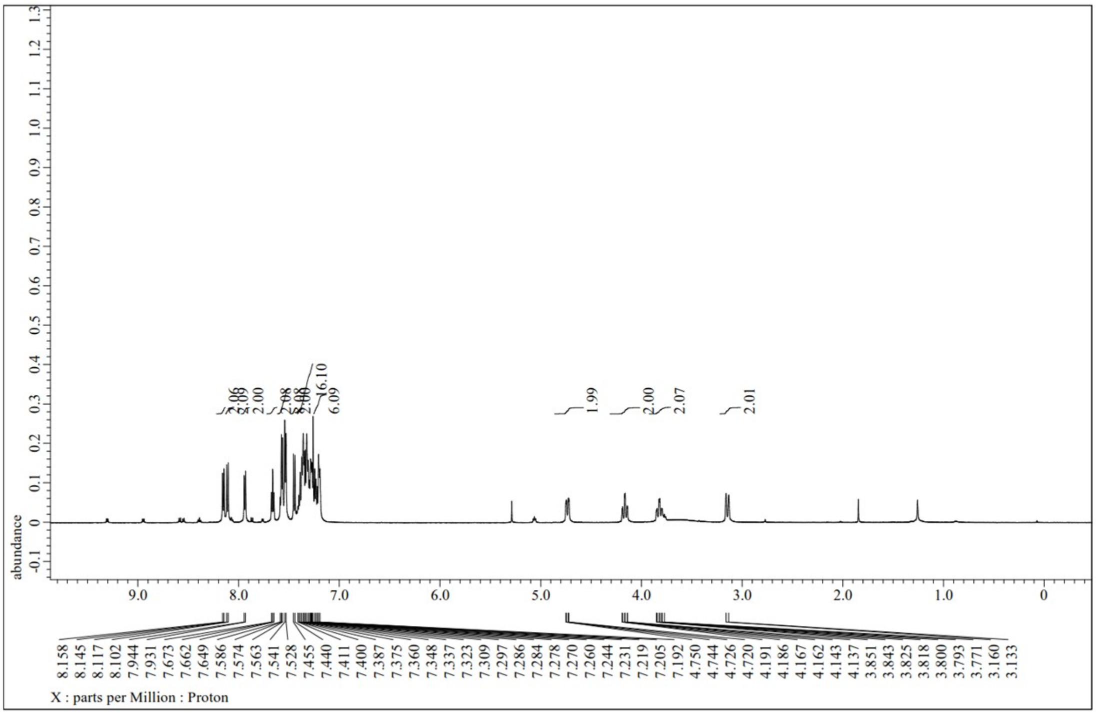
abundance
X : parts per Million : Proton
1.99
2.00
2.07
2.01
16.10
6.09
8.158
8.145
8.117
8.102
7.944
7.931
7.673
7.662
7.649
7.586
7.574
7.563
7.541
7.528
7.455
7.440
7.411
7.400
7.387
7.375
7.360
7.348
7.337
7.323
7.309
7.297
7.284
7.278
7.270
7.260
7.244
7.231
7.219
7.205
7.192
4.750
4.744
4.726
4.720
4.191
4.186
4.167
4.162
4.143
4.137
3.851
3.843
3.825
3.818
3.800
3.793
3.771
3.160
3.133

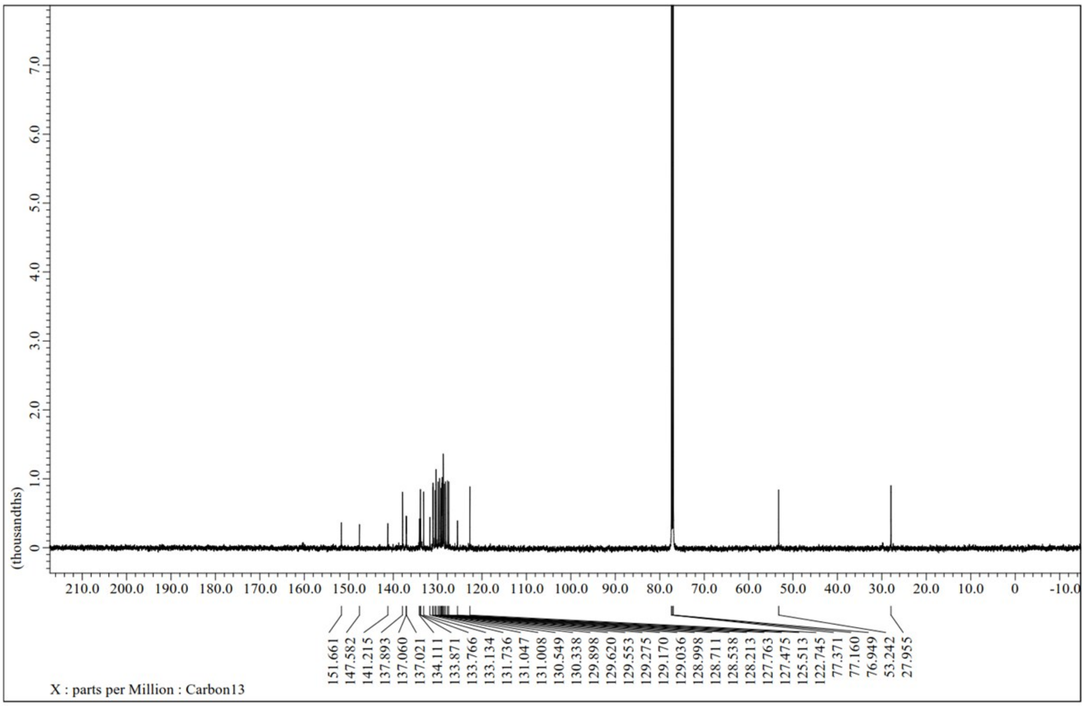
(thousandths)
X : parts per Million : Carbon13
151.661
147.582
141.215
137.893
137.060
137.021
134.111
133.871
133.766
133.134
131.736
131.047
131.008
130.549
130.338
129.898
129.620
129.553
129.275
129.170
129.036
128.998
128.711
128.538
128.213
127.763
127.475
125.513
122.745
77.371
77.160
76.949
53.242
27.955

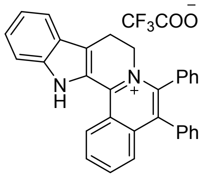
CF3COO
N
H
Ph
Ph

3ta

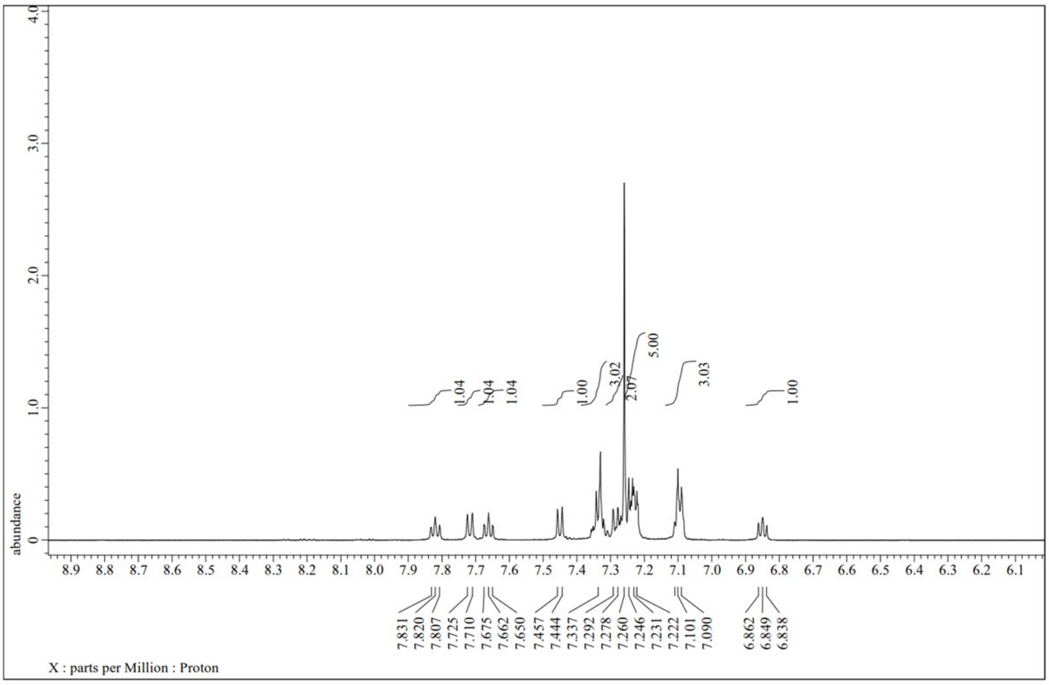
abundance
X : parts per Million : Proton
1.04
1.04
1.04
1.00
3.02
2.07
5.00
3.03
1.00
7.831
7.820
7.807
7.725
7.710
7.675
7.662
7.650
7.457
7.444
7.337
7.292
7.278
7.260
7.246
7.231
7.222
7.101
7.090
6.862
6.849
6.838

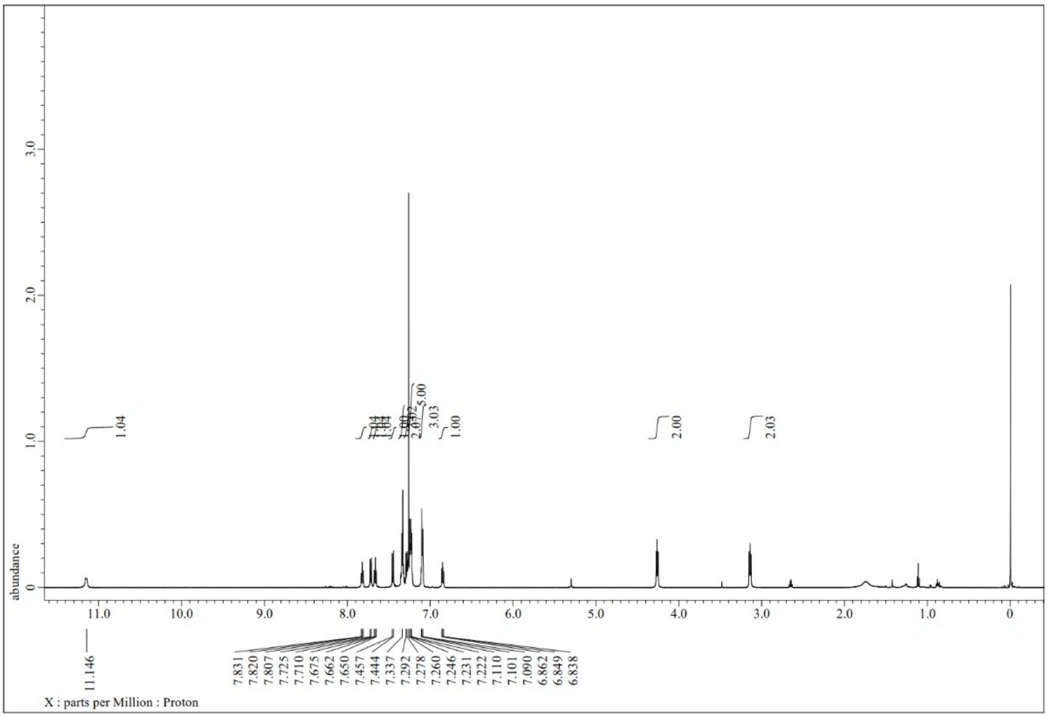
abundance
X : parts per Million : Proton
1.04
5.00
3.03
1.00
2.00
2.03
11.146
7.831
7.820
7.807
7.725
7.710
7.675
7.662
7.650
7.457
7.444
7.337
7.292
7.278
7.260
7.246
7.231
7.222
7.110
7.101
7.090
6.862
6.849
6.838

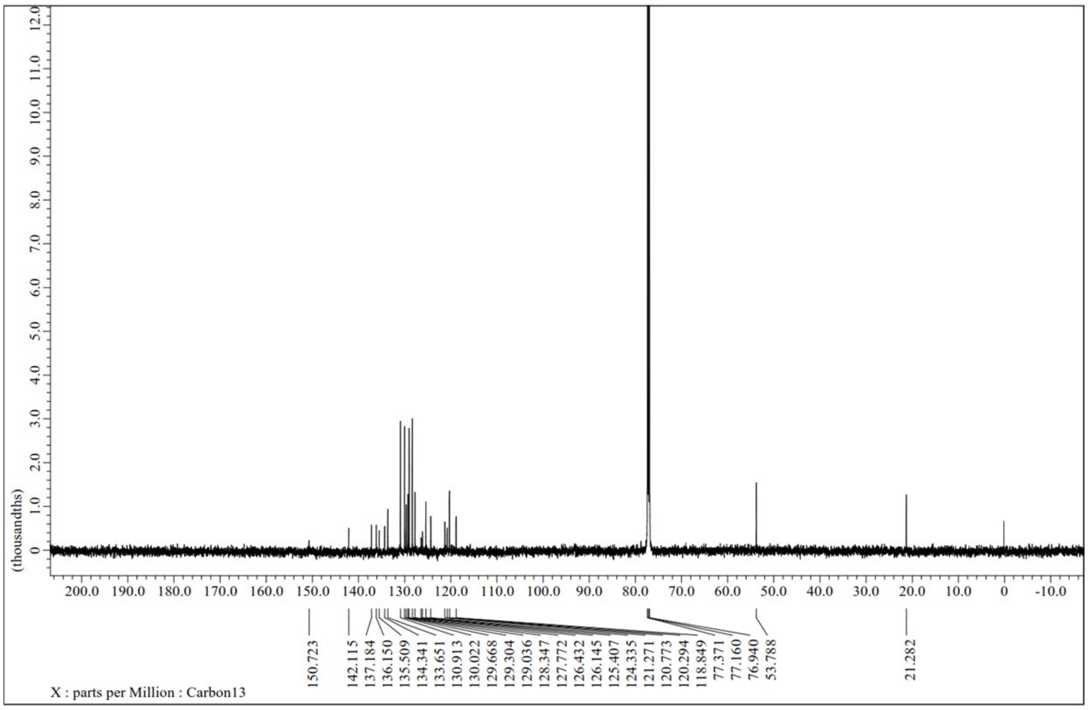
(thousandths)
X : parts per Million : Carbon13
150.723
142.115
137.184
136.150
135.509
134.341
133.651
130.913
130.022
129.668
129.304
129.036
128.347
127.772
126.432
126.145
125.407
124.335
121.271
120.773
120.294
118.849
77.371
77.160
76.940
53.788
21.282

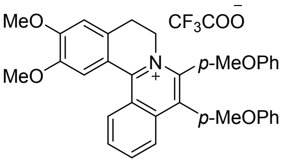
MeO
MeO
CF_3COO^-
N
+
p-MeOPh
p-MeOPh

3ab

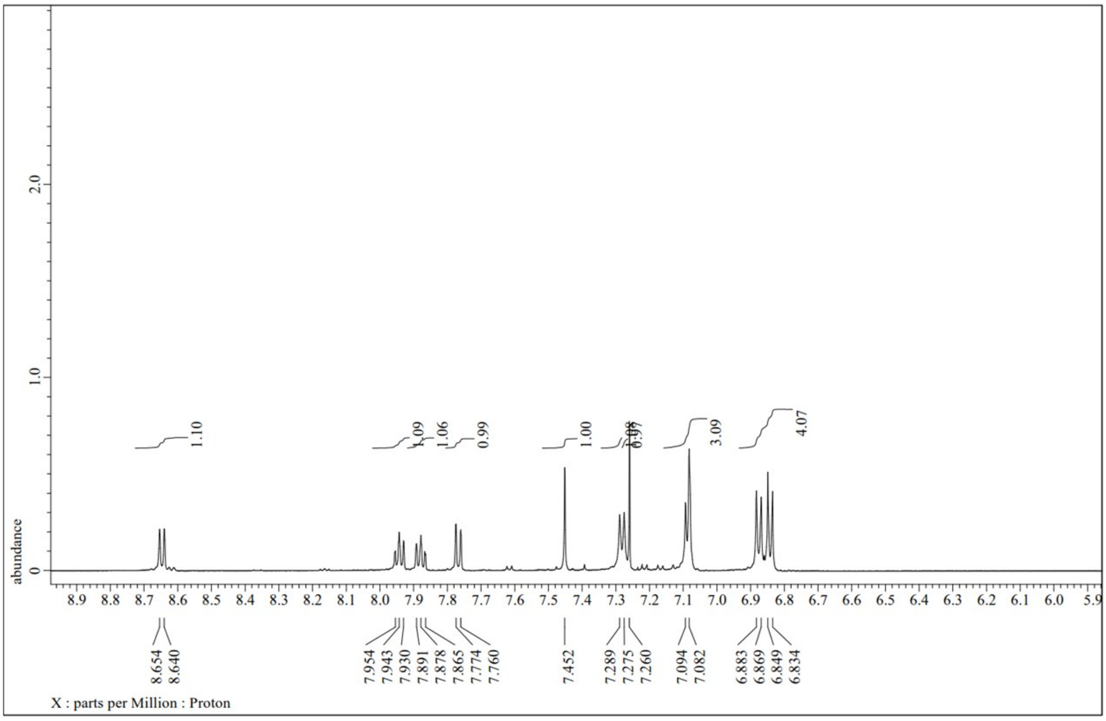
abundance
X : parts per Million : Proton
1.10
1.09
1.06
0.99
1.00
1.08
0.97
3.09
4.07
8.654
8.640
7.954
7.943
7.930
7.891
7.878
7.865
7.774
7.760
7.452
7.289
7.275
7.260
7.094
7.082
6.883
6.869
6.849
6.834

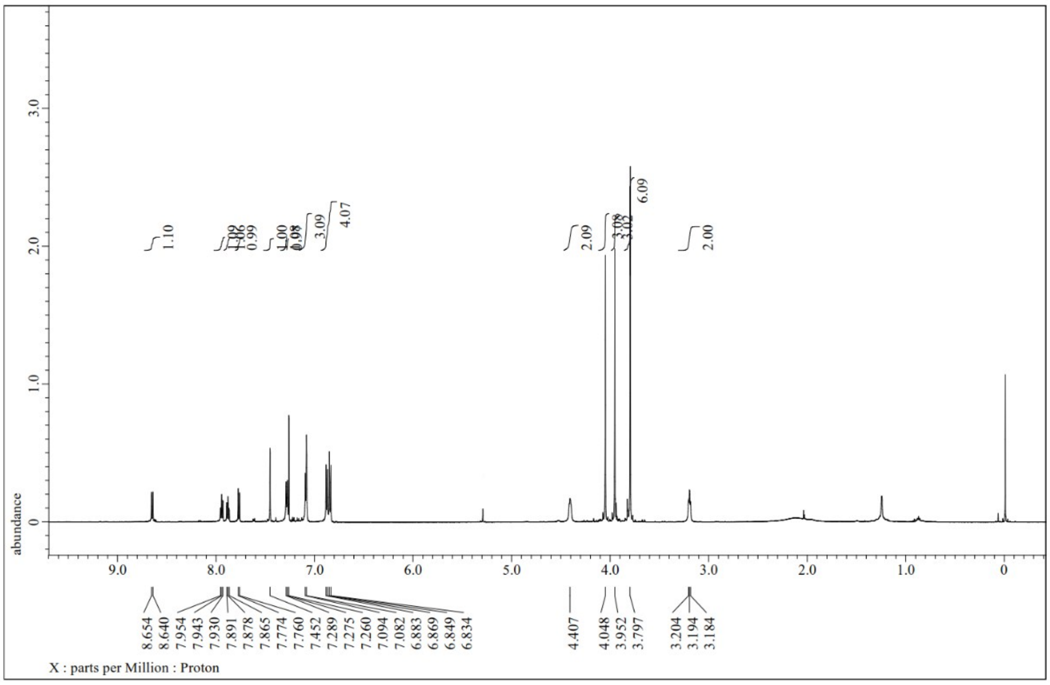

abundance
3.0
2.0
1.0
0
1.10
1.02
1.08
0.99
1.00
0.98
3.09
4.07
2.09
3.08
3.02
6.09
2.00
9.0
8.0
7.0
6.0
5.0
4.0
3.0
2.0
1.0
0
8.654
8.640
7.954
7.943
7.930
7.891
7.878
7.865
7.774
7.760
7.452
7.289
7.275
7.260
7.094
7.082
6.883
6.869
6.849
6.834
4.407
4.048
3.952
3.797
3.204
3.194
3.184
X : parts per Million : Proton

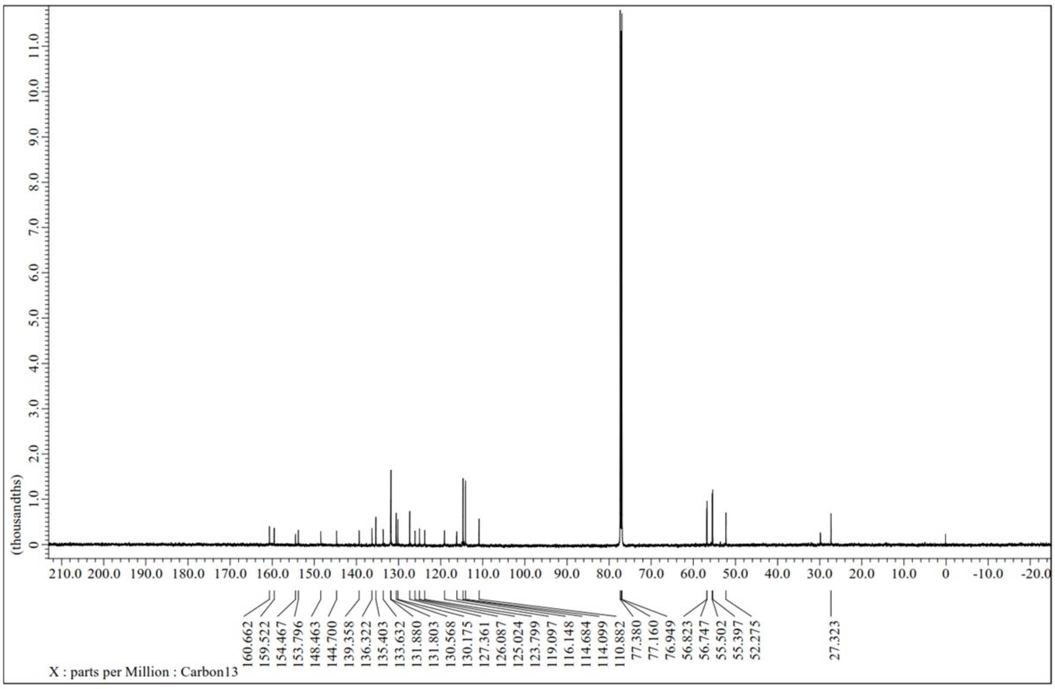

(thousandths)
11.0
10.0
9.0
8.0
7.0
6.0
5.0
4.0
3.0
2.0
1.0
0
210.0 200.0 190.0 180.0 170.0 160.0 150.0 140.0 130.0 120.0 110.0 100.0 90.0 80.0 70.0 60.0 50.0 40.0 30.0 20.0 10.0 0 -10.0 -20.0
160.662
159.522
154.467
153.796
148.463
144.700
139.358
136.322
135.403
133.632
131.880
131.803
130.568
130.175
127.361
126.087
125.024
123.799
119.097
116.148
114.684
114.099
110.882
77.380
77.160
76.949
56.823
56.747
55.502
55.397
52.275
27.323
X : parts per Million : Carbon13

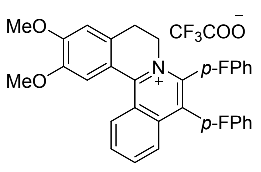

MeO
MeO
CF3COO
N
+
p-FPh
p-FPh

3ac

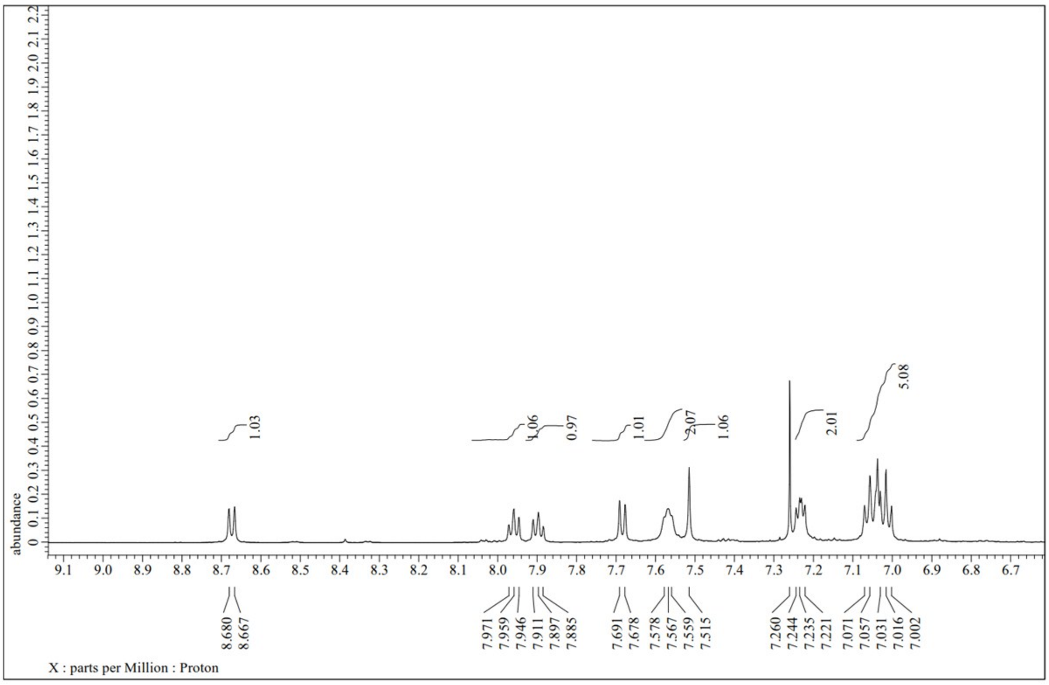
abundance
X : parts per Million : Proton

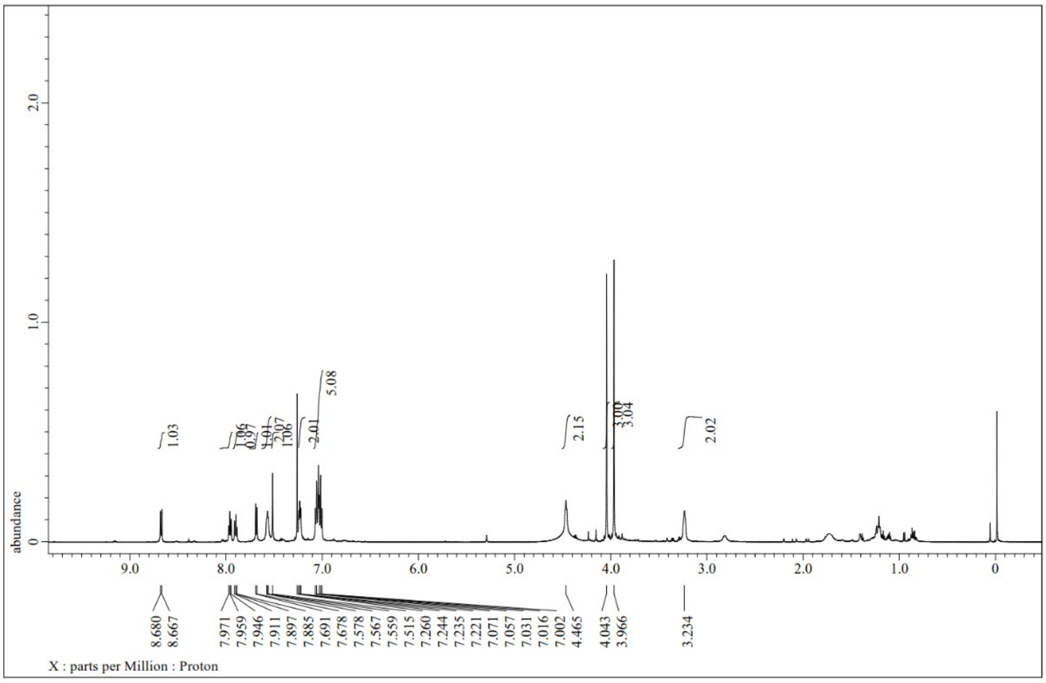
abundance
X : parts per Million : Proton

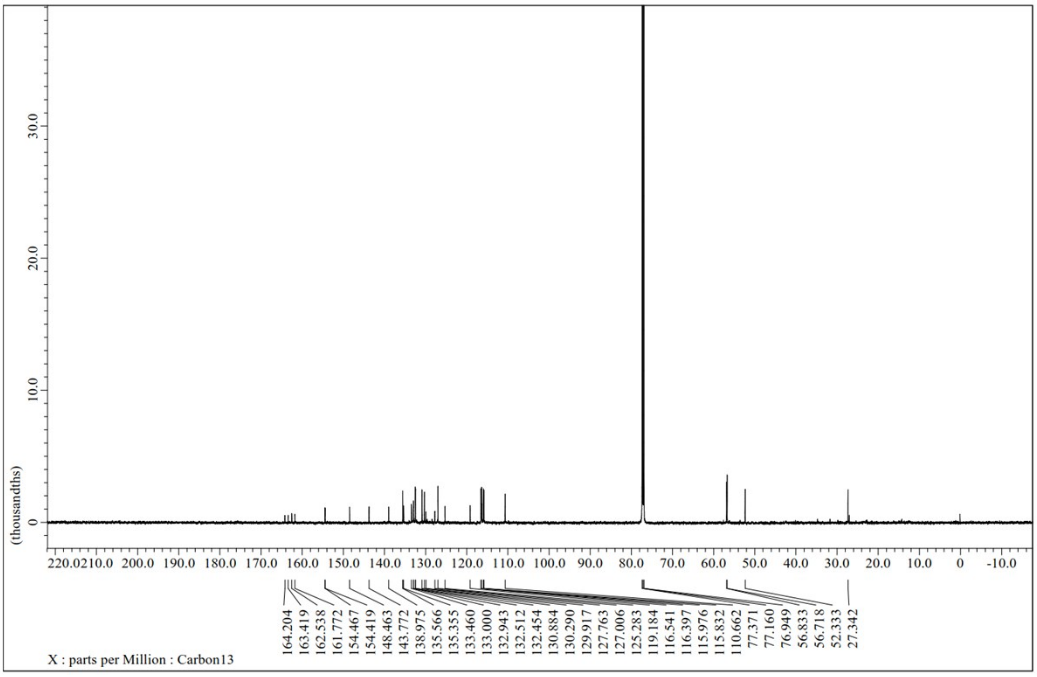
(thousandths)
X : parts per Million : Carbon13
164.204
163.419
162.538
161.772
154.467
154.419
148.463
143.772
138.975
135.566
135.355
133.460
133.000
132.943
132.512
132.454
130.884
130.290
129.917
127.763
127.006
125.283
119.184
116.541
116.397
115.976
115.832
110.662
77.371
77.160
76.949
56.833
56.718
52.333
27.342

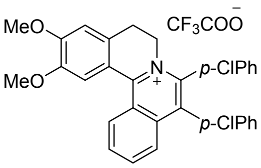
MeO
MeO
CF3COO
N
p-ClPh
p-ClPh

3ad

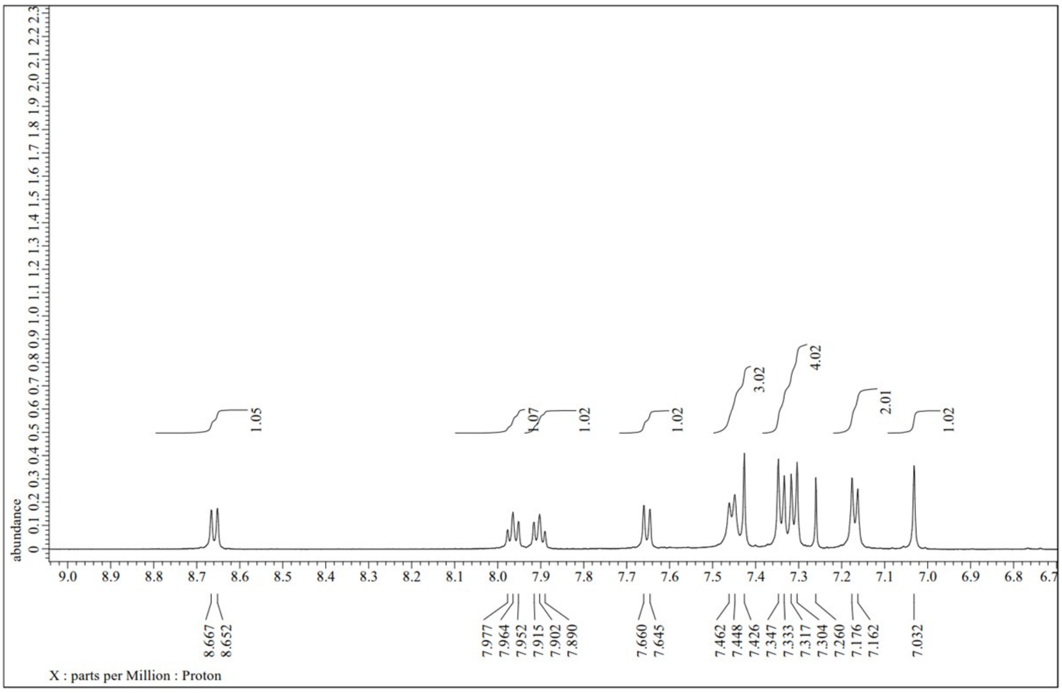
abundance
X : parts per Million : Proton
1.05
1.07
1.02
1.02
3.02
4.02
2.01
1.02
8.667
8.652
7.977
7.964
7.952
7.915
7.902
7.890
7.660
7.645
7.462
7.448
7.426
7.347
7.333
7.317
7.304
7.260
7.176
7.162
7.032

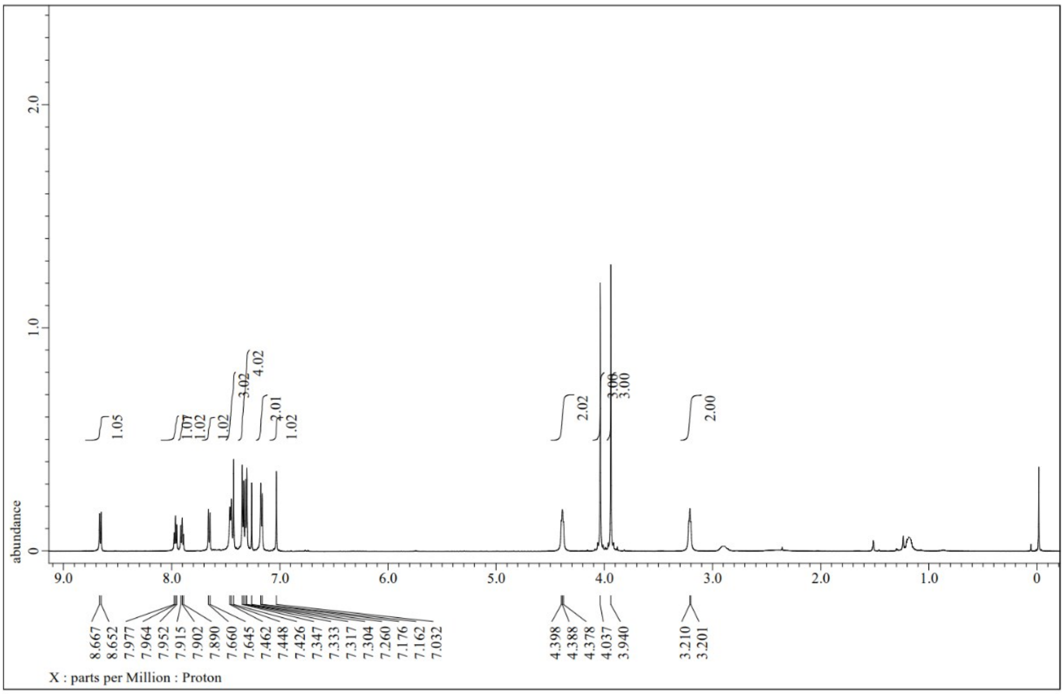
abundance
X : parts per Million : Proton
8.667
8.652
7.977
7.964
7.952
7.915
7.902
7.890
7.660
7.645
7.462
7.448
7.426
7.347
7.333
7.317
7.304
7.260
7.176
7.162
7.032
4.398
4.388
4.378
4.037
3.940
3.210
3.201

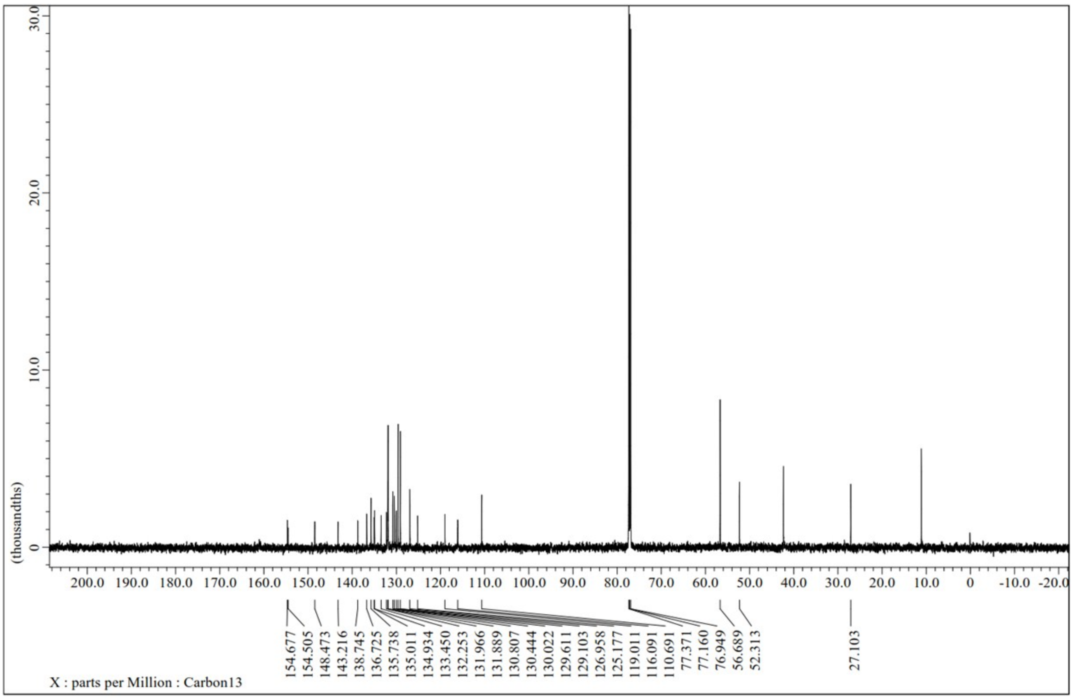
(thousandths)
X : parts per Million : Carbon13
154.677
154.505
148.473
143.216
138.745
136.725
135.738
135.011
134.934
133.450
132.253
131.966
131.889
130.807
130.444
130.022
129.611
129.103
126.958
125.177
119.011
116.091
110.691
77.371
77.160
76.949
56.689
52.313
27.103

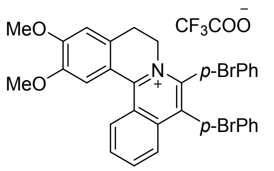
MeO
MeO
CF3COO
p-BrPh
p-BrPh

3ae

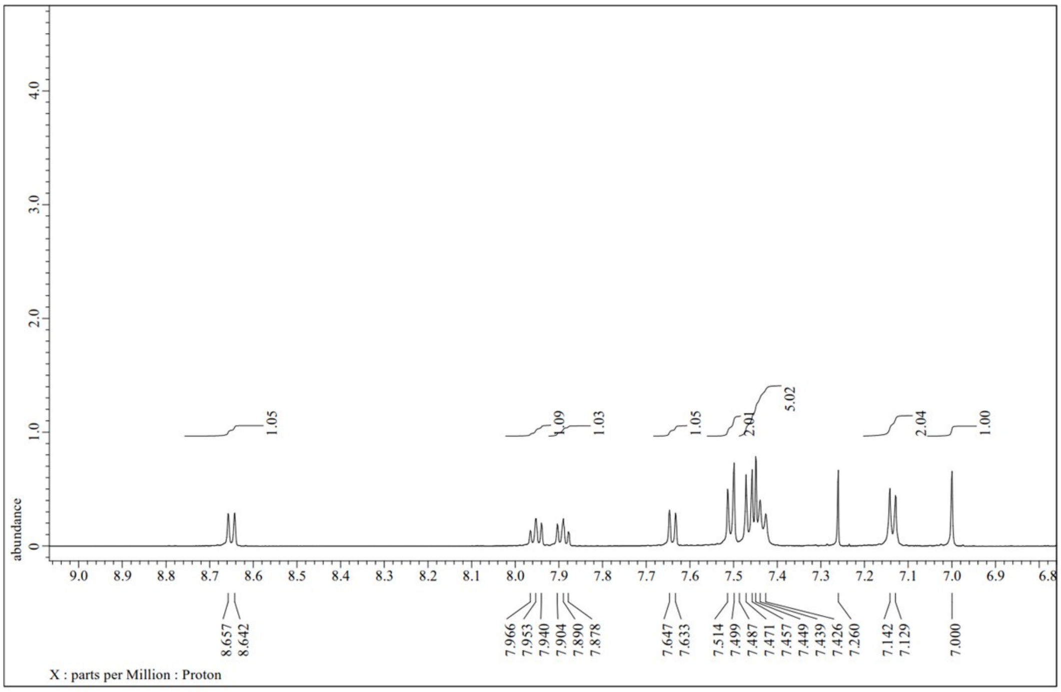
abundance
X : parts per Million : Proton

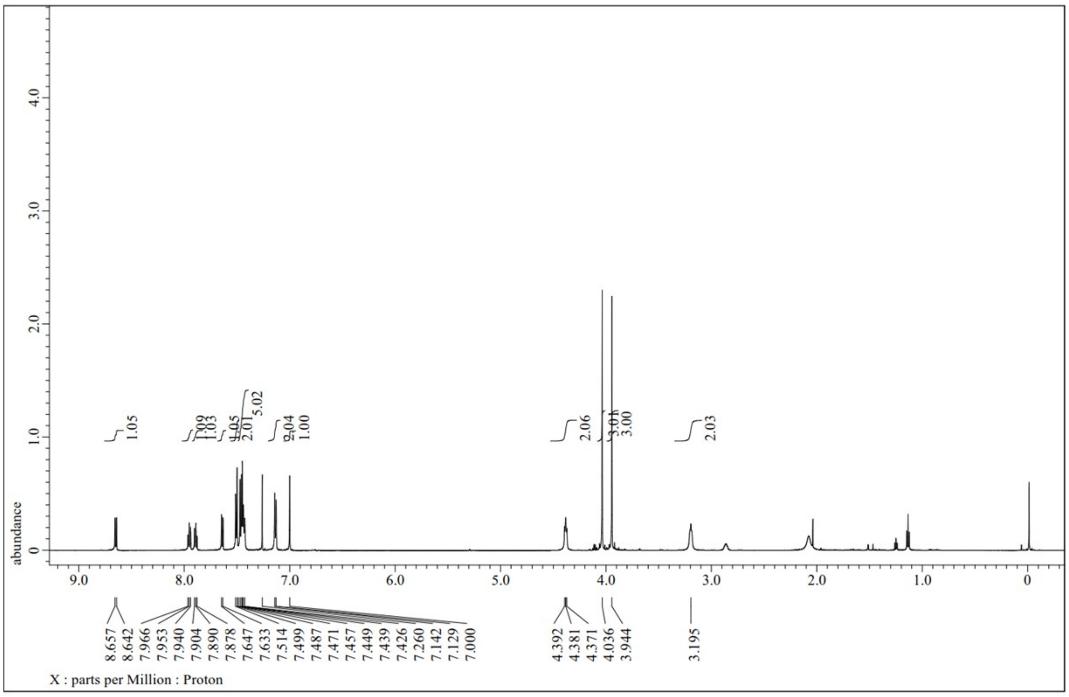
abundance
X : parts per Million : Proton

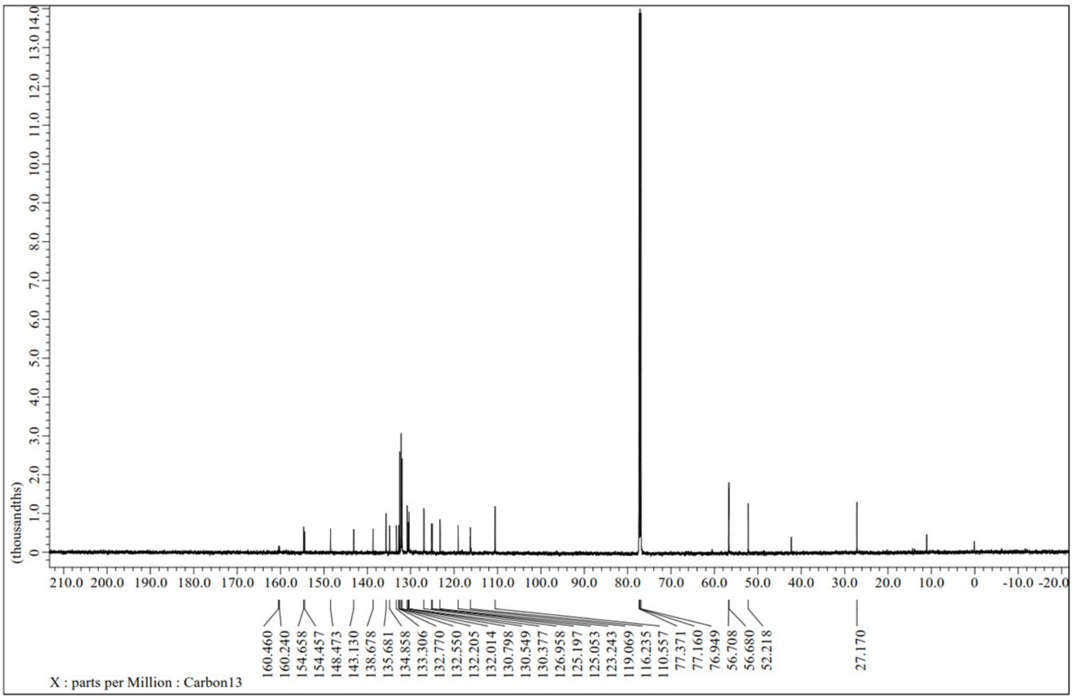
(thousandths)
X : parts per Million : Carbon13
160.460
160.240
154.658
154.457
148.473
143.130
138.678
135.681
134.858
133.306
132.770
132.550
132.205
132.014
130.798
130.549
130.377
126.958
125.197
125.053
123.243
119.069
116.235
110.557
77.371
77.160
76.949
56.708
56.680
52.218
27.170

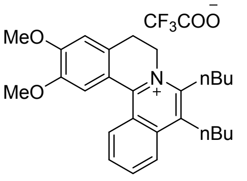
MeO
MeO
CF3COO
N
nBu
nBu

3af

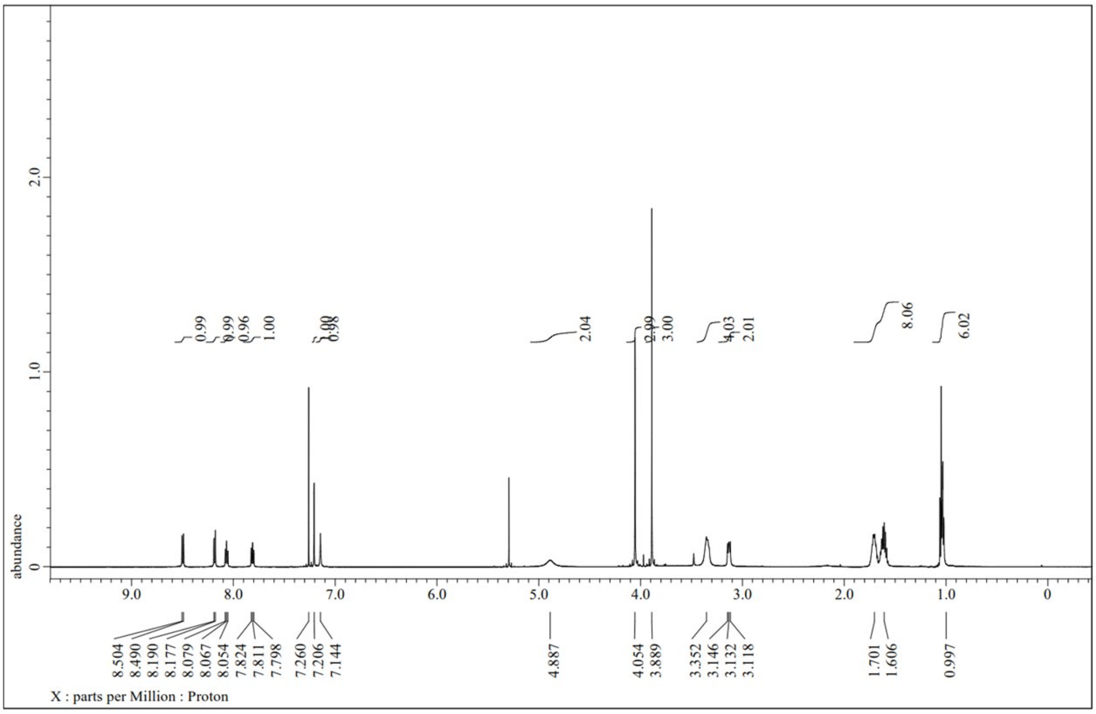
abundance
X : parts per Million : Proton
8.504
8.490
8.190
8.177
8.079
8.067
8.054
7.824
7.811
7.798
7.260
7.206
7.144
4.887
4.054
3.889
3.352
3.146
3.132
3.118
1.701
1.606
0.997
2.04
3.00
2.01
8.06
6.02

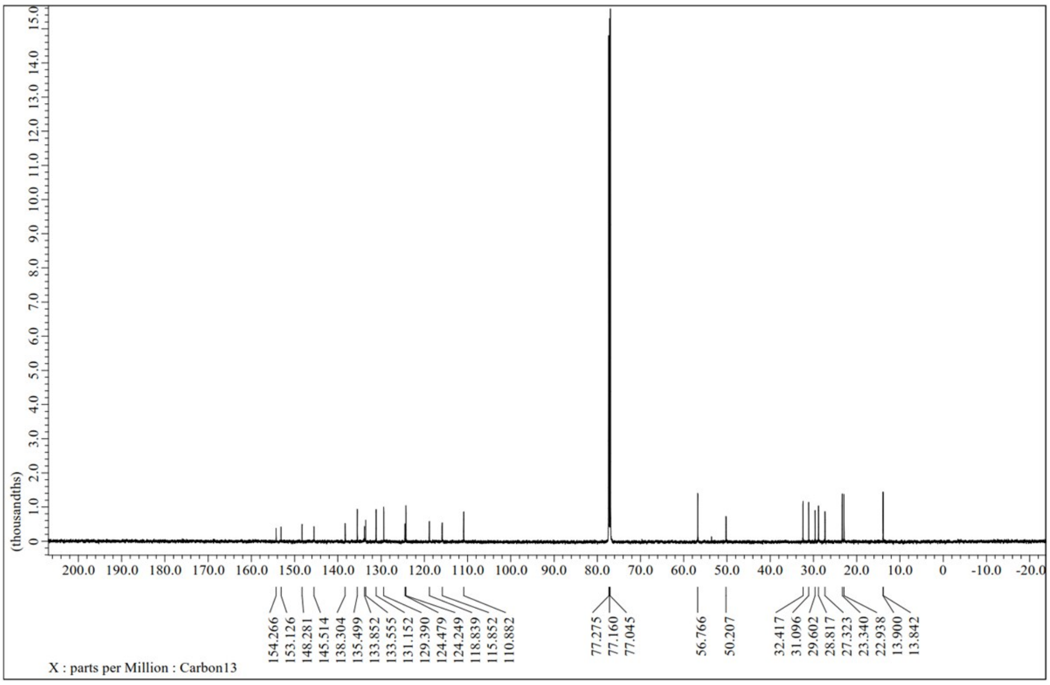
(thousandths)
15.0 14.0 13.0 12.0 11.0 10.0 9.0 8.0 7.0 6.0 5.0 4.0 3.0 2.0 1.0 0
200.0 190.0 180.0 170.0 160.0 150.0 140.0 130.0 120.0 110.0 100.0 90.0 80.0 70.0 60.0 50.0 40.0 30.0 20.0 10.0 0 -10.0 -20.0
154.266
153.126
148.281
145.514
138.304
135.499
133.852
133.555
131.152
129.390
124.479
124.249
118.839
115.852
110.882
77.275
77.160
77.045
56.766
50.207
32.417
31.096
29.602
28.817
27.323
23.340
22.938
13.900
13.842
X : parts per Million : Carbon13

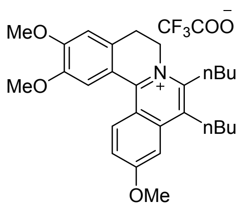
MeO
MeO
CF3COO
N
+
nBu
nBu
OMe

3bf

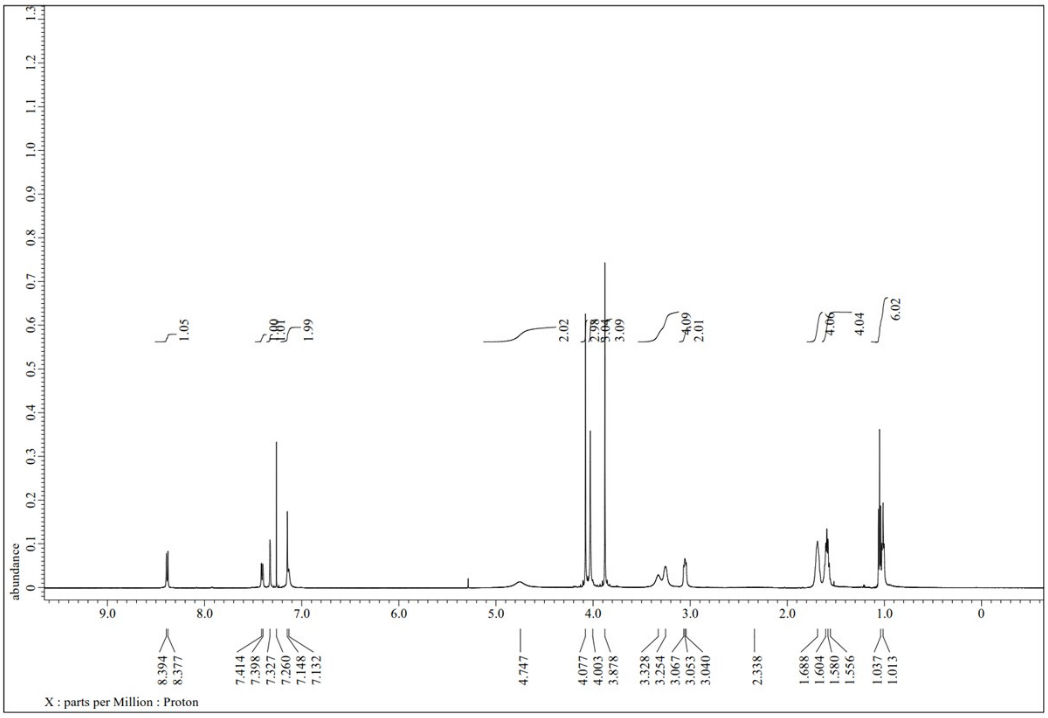
abundance
1.3 1.2 1.1 1.0 0.9 0.8 0.7 0.6 0.5 0.4 0.3 0.2 0.1 0
1.05
1.01
1.99
2.02
3.09
4.09
2.01
4.06
4.04
6.02
9.0 8.0 7.0 6.0 5.0 4.0 3.0 2.0 1.0 0
8.394
8.377
7.414
7.398
7.327
7.260
7.148
7.132
4.747
4.077
4.003
3.878
3.328
3.254
3.067
3.053
3.040
2.338
1.688
1.604
1.580
1.556
1.037
1.013
X : parts per Million : Proton

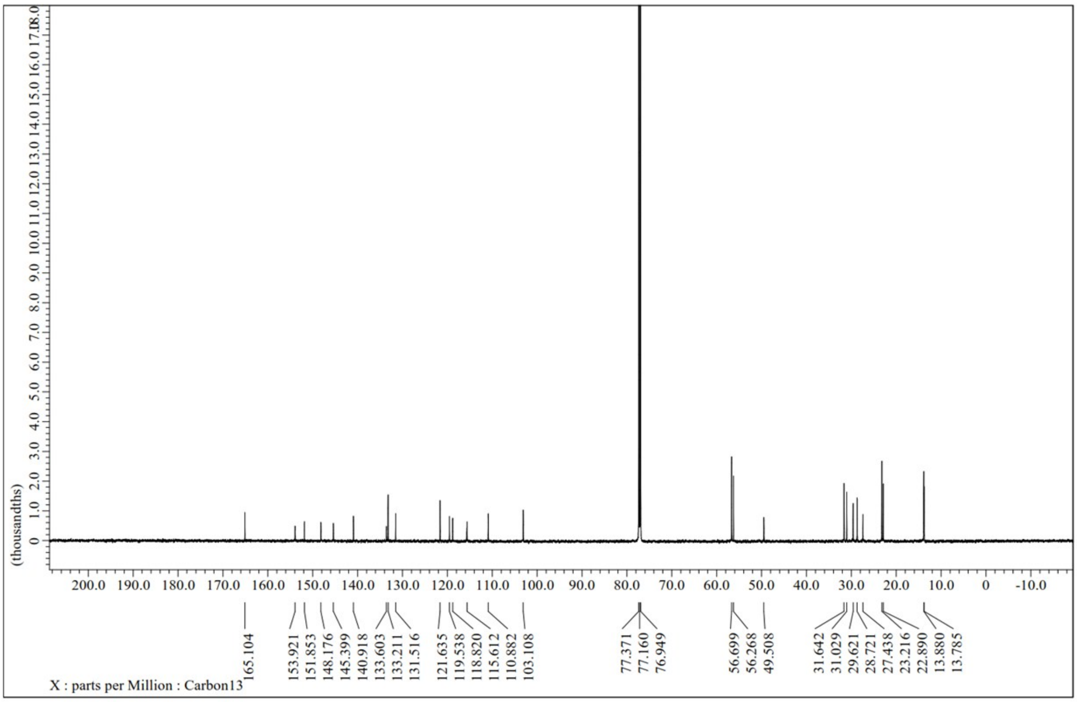
(thousandths)
165.104
153.921
151.853
148.176
145.399
140.918
133.603
133.211
131.516
121.635
119.538
118.820
115.612
110.882
103.108
77.371
77.160
76.949
56.699
56.268
49.508
31.642
31.029
29.621
28.721
27.438
23.216
22.890
13.880
13.785
X : parts per Million : Carbon13

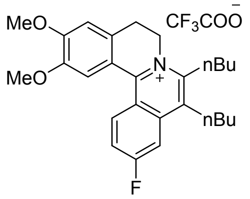
MeO
MeO
CF_3COO^-
N
+
nBu
nBu
F

3df

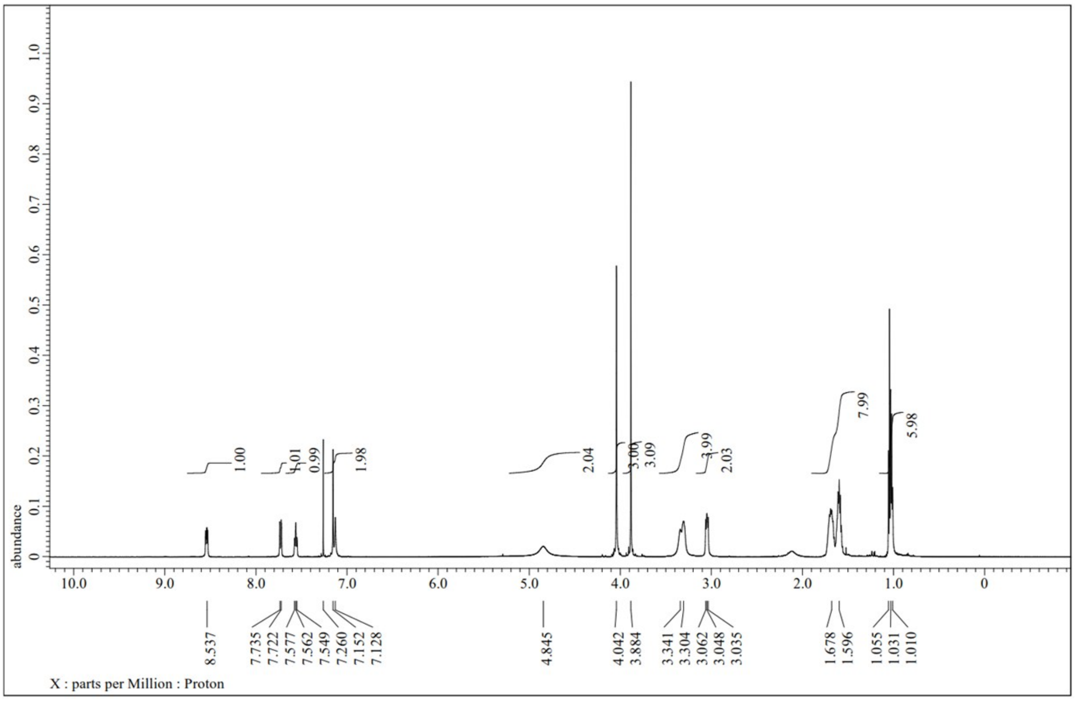
abundance
1.00
1.01
0.99
1.98
2.04
3.00
3.09
3.99
2.03
7.99
5.98
8.537
7.735
7.722
7.577
7.562
7.549
7.260
7.152
7.128
4.845
4.042
3.884
3.341
3.304
3.062
3.048
3.035
1.678
1.596
1.055
1.031
1.010
X : parts per Million : Proton

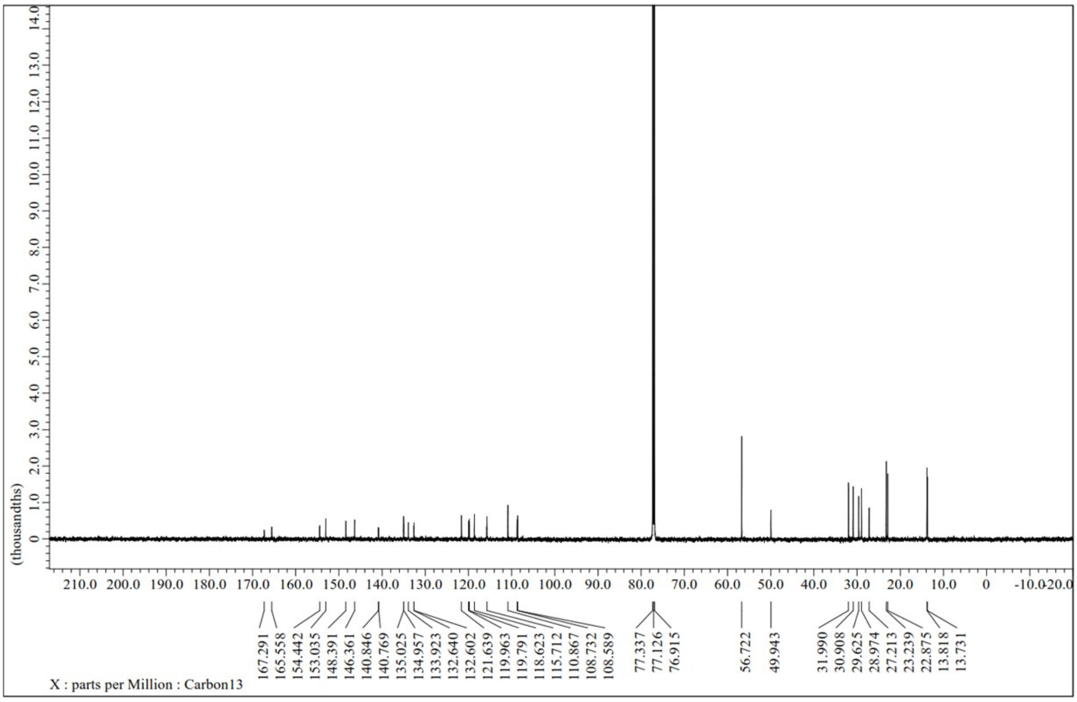

(thousandths)
X : parts per Million : Carbon13
167.291
165.558
154.442
153.035
148.391
146.361
140.846
140.769
135.025
134.957
133.923
132.640
132.602
121.639
119.963
119.791
118.623
115.712
110.867
108.732
108.589
77.337
77.126
76.915
56.722
49.943
31.990
30.908
29.625
28.974
27.213
23.239
22.875
13.818
13.731

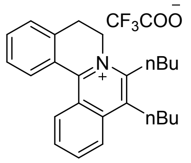

CF3COO
N
+
nBu
nBu

3qf

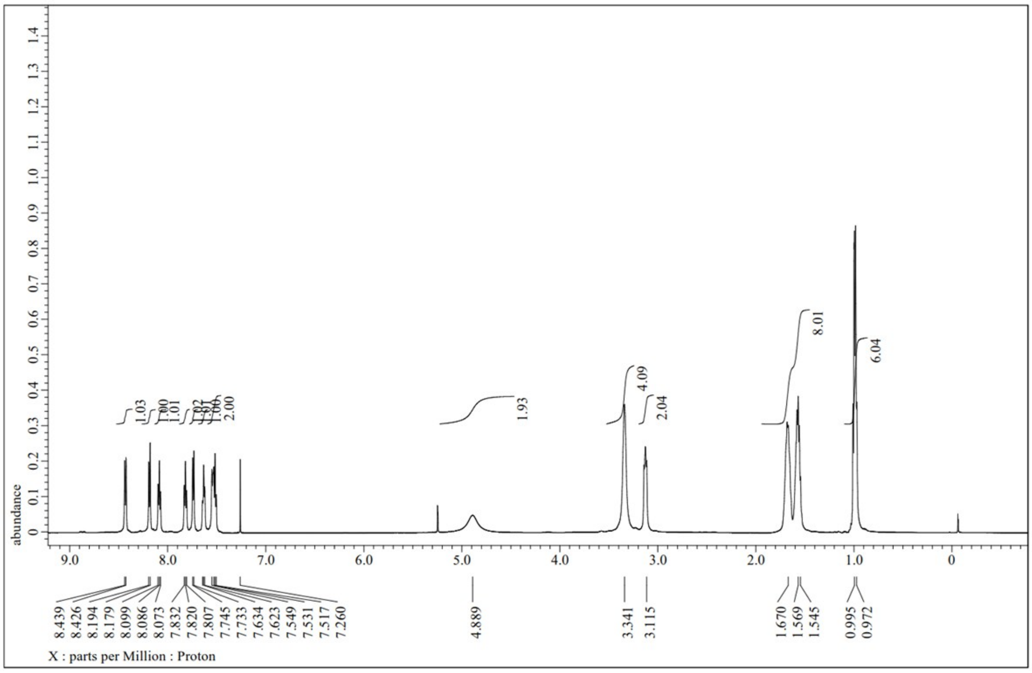

abundance
X : parts per Million : Proton
1.03
1.00
1.01
2.00
1.93
4.09
2.04
8.01
6.04
8.439
8.426
8.194
8.179
8.099
8.086
8.073
7.832
7.820
7.807
7.745
7.733
7.634
7.623
7.549
7.531
7.517
7.260
4.889
3.341
3.115
1.670
1.569
1.545
0.995
0.972

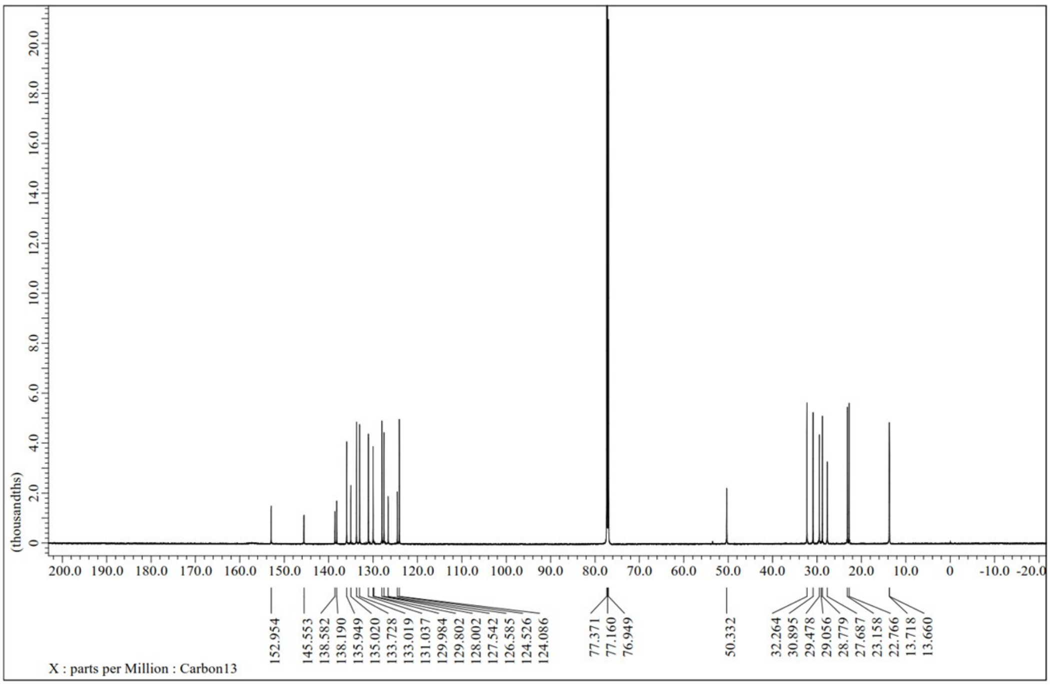
(thousandths)
X : parts per Million : Carbon13
152.954
145.553
138.582
138.190
135.949
135.020
133.728
133.019
131.037
129.984
129.802
128.002
127.542
126.585
124.526
124.086
77.371
77.160
76.949
50.332
32.264
30.895
29.478
29.056
28.779
27.687
23.158
22.766
13.718
13.660

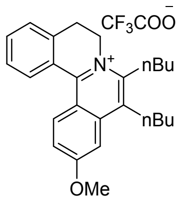
CF3COO
N+
nBu
nBu
OMe

3of

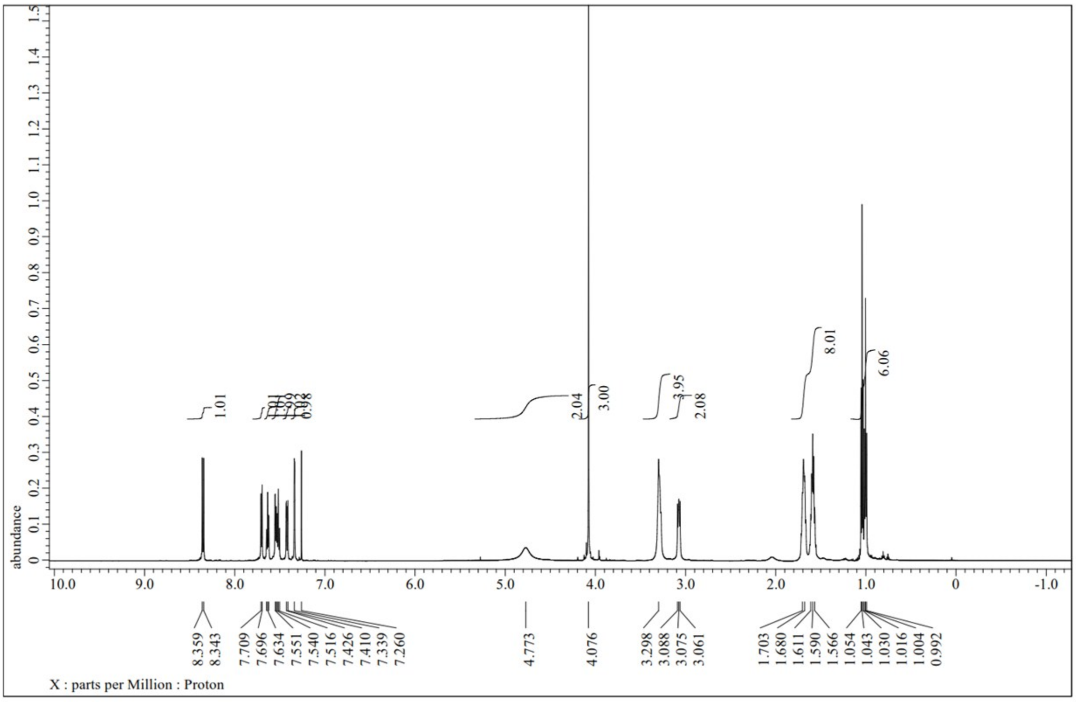
abundance
X : parts per Million : Proton
1.01
2.04
3.00
3.95
2.08
8.01
6.06
8.359
8.343
7.709
7.696
7.634
7.551
7.540
7.516
7.426
7.410
7.339
7.260
4.773
4.076
3.298
3.088
3.075
3.061
1.703
1.680
1.611
1.590
1.566
1.054
1.043
1.030
1.016
1.004
0.992

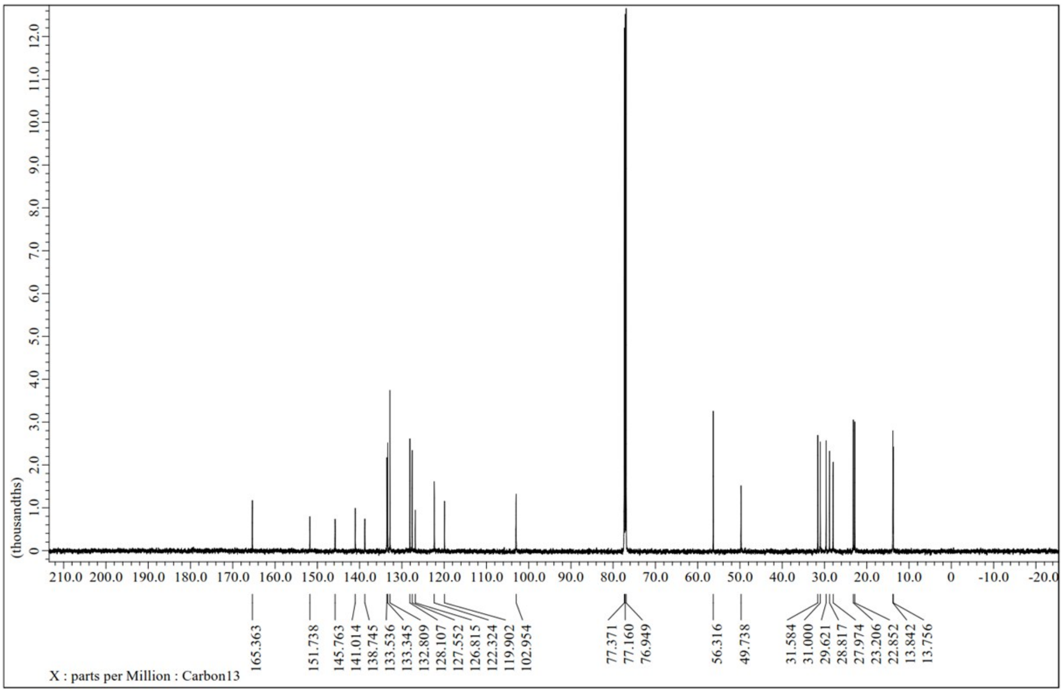
(thousandths)
X : parts per Million : Carbon13
165.363
151.738
145.763
141.014
138.745
133.536
133.345
132.809
128.107
127.552
126.815
122.324
119.902
102.954
77.371
77.160
76.949
56.316
49.738
31.584
31.000
29.621
28.817
27.974
23.206
22.852
13.842
13.756

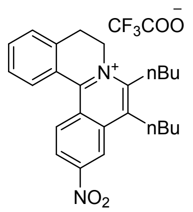
CF3COO
N+
nBu
nBu
NO2

3pf

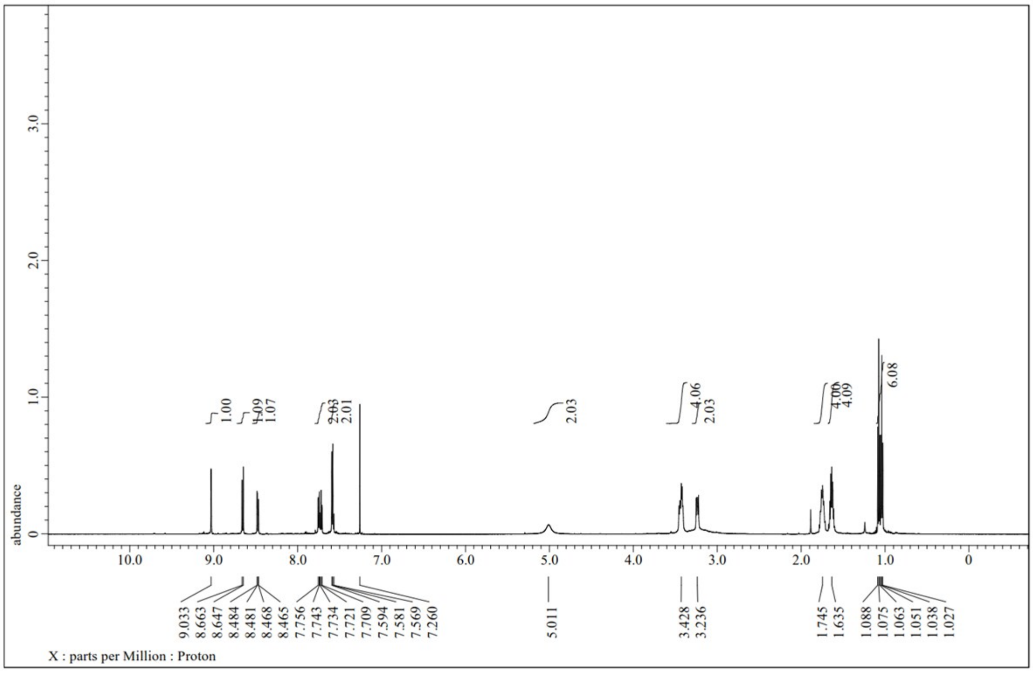
abundance
X : parts per Million : Proton
1.00
1.09
1.07
2.03
2.01
2.03
4.06
2.03
4.00
4.09
6.08
9.033
8.663
8.647
8.484
8.481
8.468
8.465
7.756
7.743
7.734
7.721
7.709
7.594
7.581
7.569
7.260
5.011
3.428
3.236
1.745
1.635
1.088
1.075
1.063
1.051
1.038
1.027

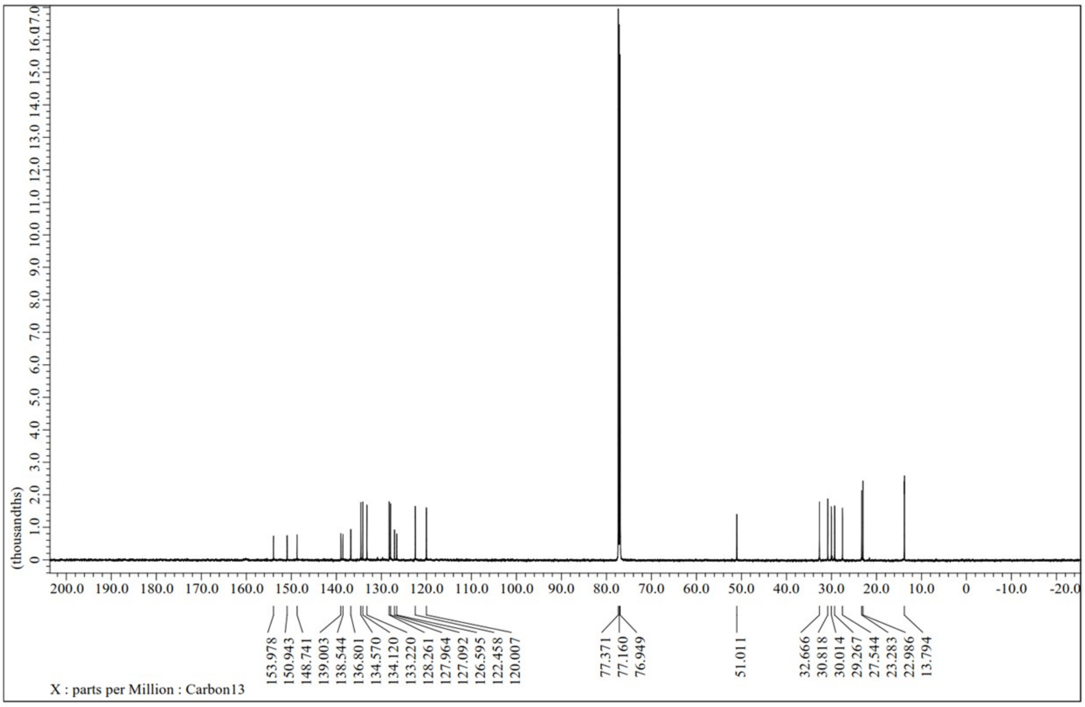
(thousandths)
X : parts per Million : Carbon13
153.978
150.943
148.741
139.003
138.544
136.801
134.570
134.120
133.220
128.261
127.964
127.092
126.595
122.458
120.007
77.371
77.160
76.949
51.011
32.666
30.818
30.014
29.267
27.544
23.283
22.986
13.794

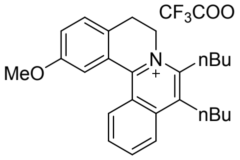
CF3COO
MeO
N
nBu
nBu

3rf

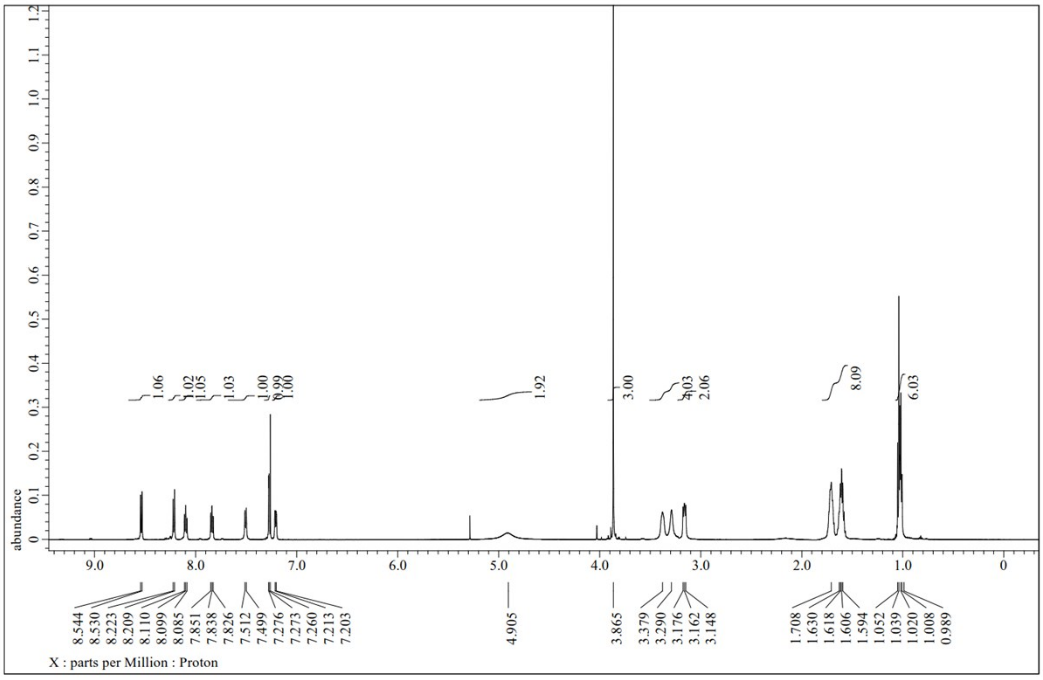
abundance
X : parts per Million : Proton
1.06
1.02
1.05
1.03
1.00
0.99
1.00
1.92
3.00
4.03
2.06
8.09
6.03
8.544
8.530
8.223
8.209
8.110
8.099
8.085
7.851
7.838
7.826
7.512
7.499
7.276
7.273
7.260
7.213
7.203
4.905
3.865
3.379
3.290
3.176
3.162
3.148
1.708
1.630
1.618
1.606
1.594
1.052
1.039
1.020
1.008
0.989

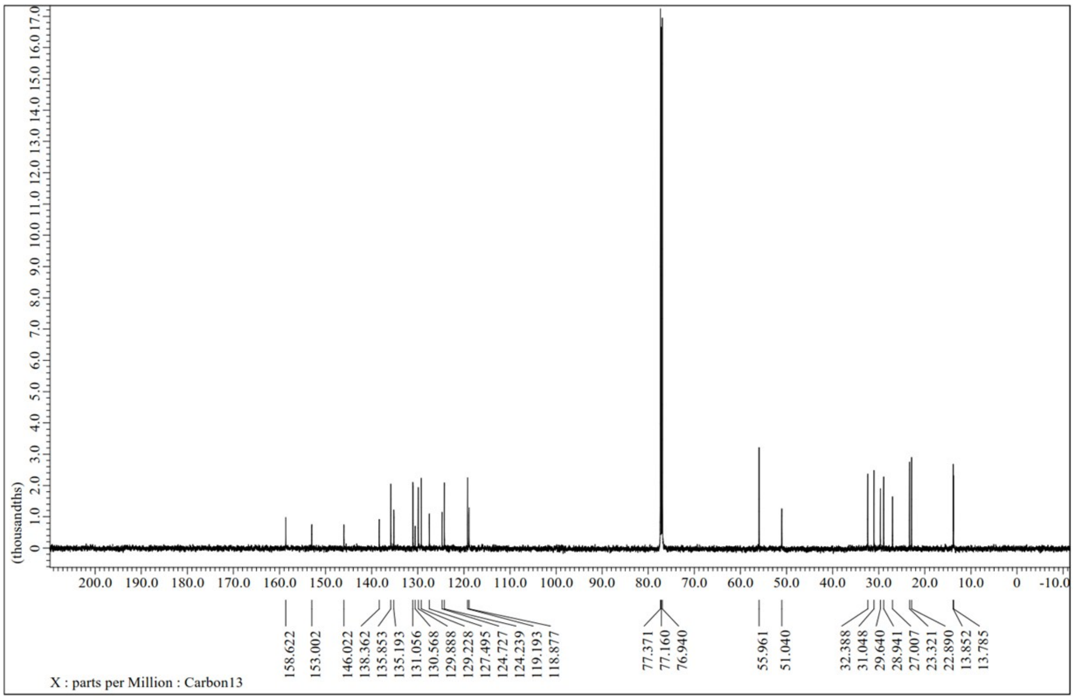
158.622
153.002
146.022
138.362
135.853
135.193
131.056
130.568
129.888
129.228
127.495
124.727
124.239
119.193
118.877
77.371
77.160
76.940
55.961
51.040
32.388
31.048
29.640
28.941
27.007
23.321
22.890
13.852
13.785
(thousandths)
X : parts per Million : Carbon13

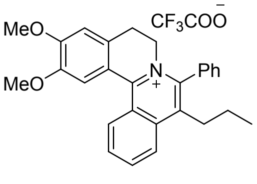
MeO
MeO
CF3COO
N
Ph

3ag

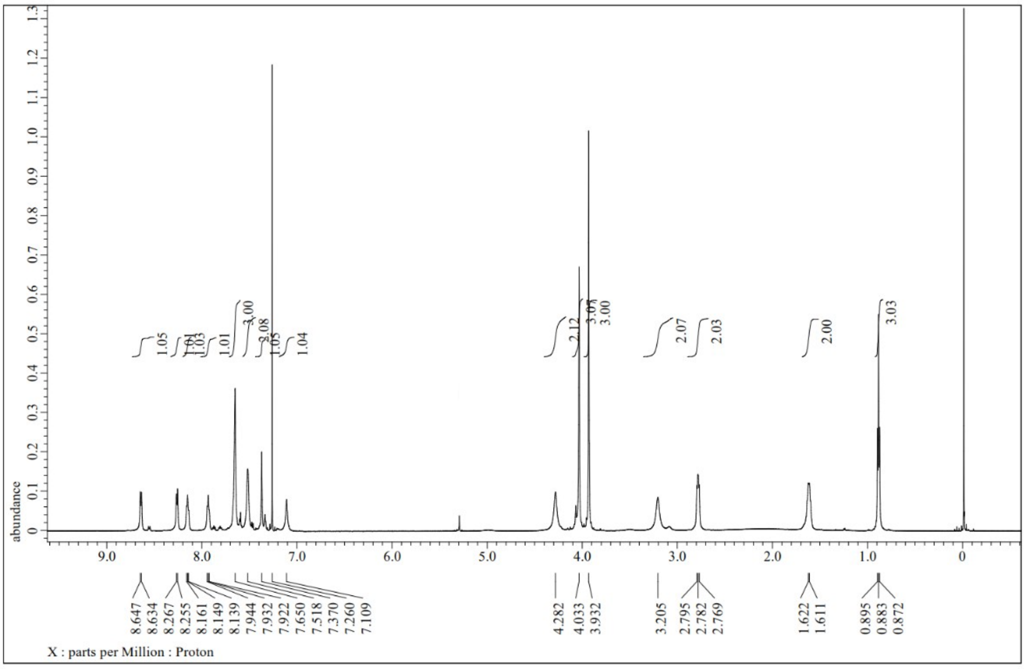
8.647
8.634
8.267
8.255
8.161
8.149
8.139
7.944
7.932
7.922
7.650
7.518
7.370
7.260
7.109
4.282
4.033
3.932
3.205
2.795
2.782
2.769
1.622
1.611
0.895
0.883
0.872
abundance
X : parts per Million : Proton

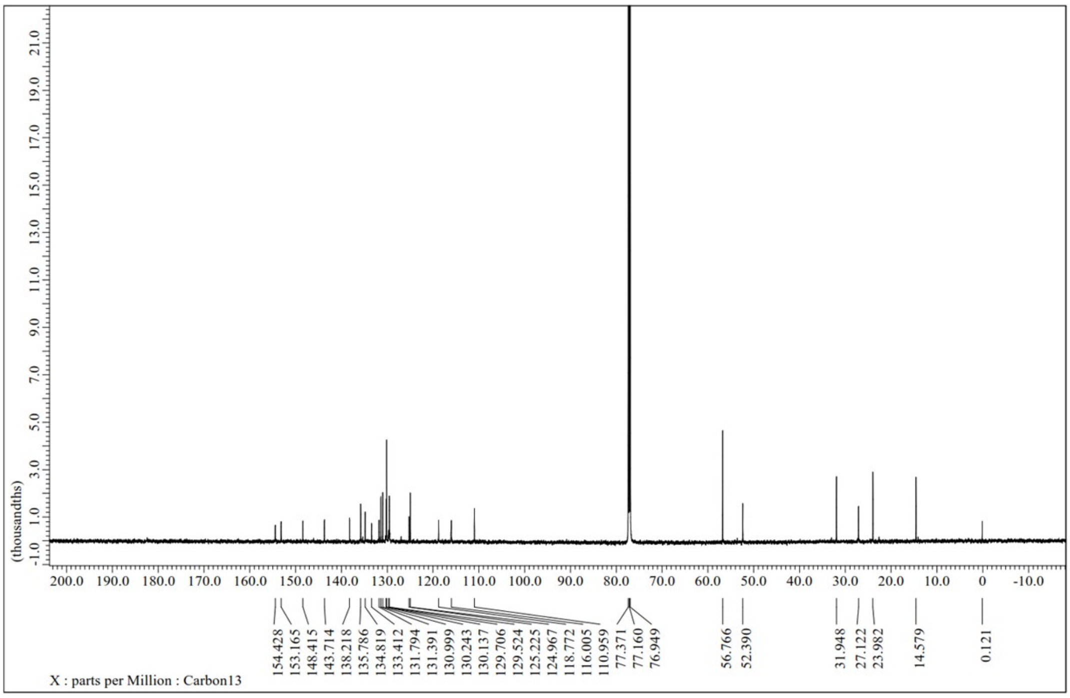
(thousandths)
X : parts per Million : Carbon13
154.428
153.165
148.415
143.714
138.218
135.786
134.819
133.412
131.794
131.391
130.999
130.243
130.137
129.706
129.524
125.225
124.967
118.772
116.005
110.959
77.371
77.160
76.949
56.766
52.390
31.948
27.122
23.982
14.579
0.121

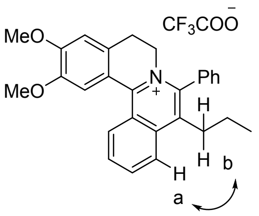
MeO
MeO
CF_3COO^-
Ph
H
H
H
a
b

3ag

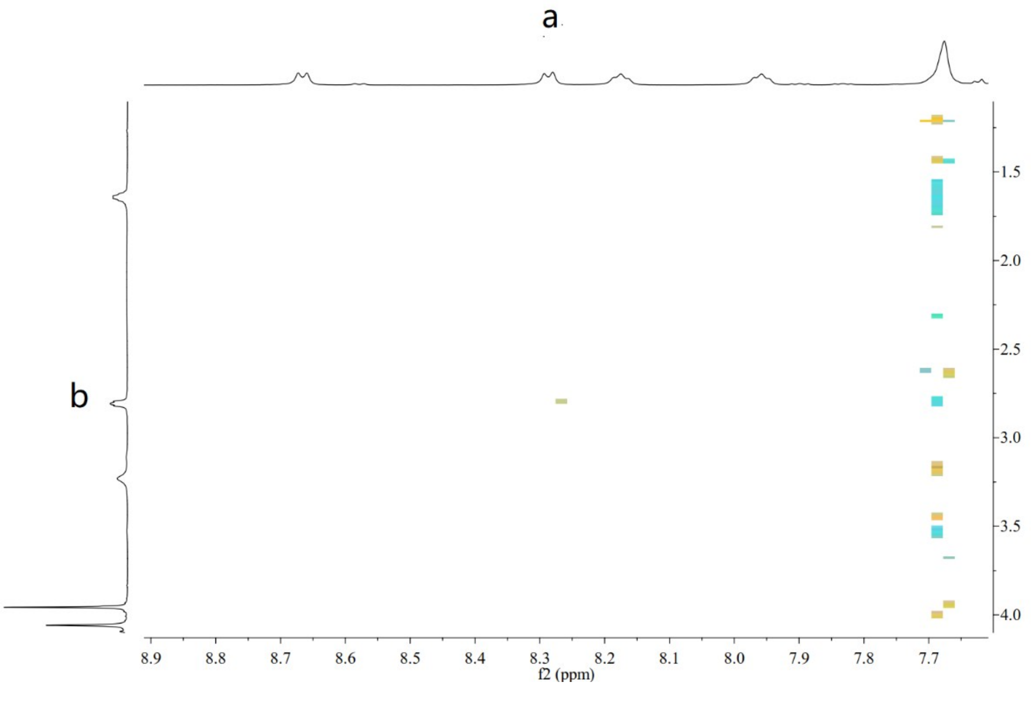
a
b
f2 (ppm)

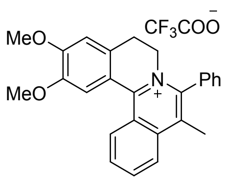
MeO
MeO
CF3COO
N
Ph

3ah

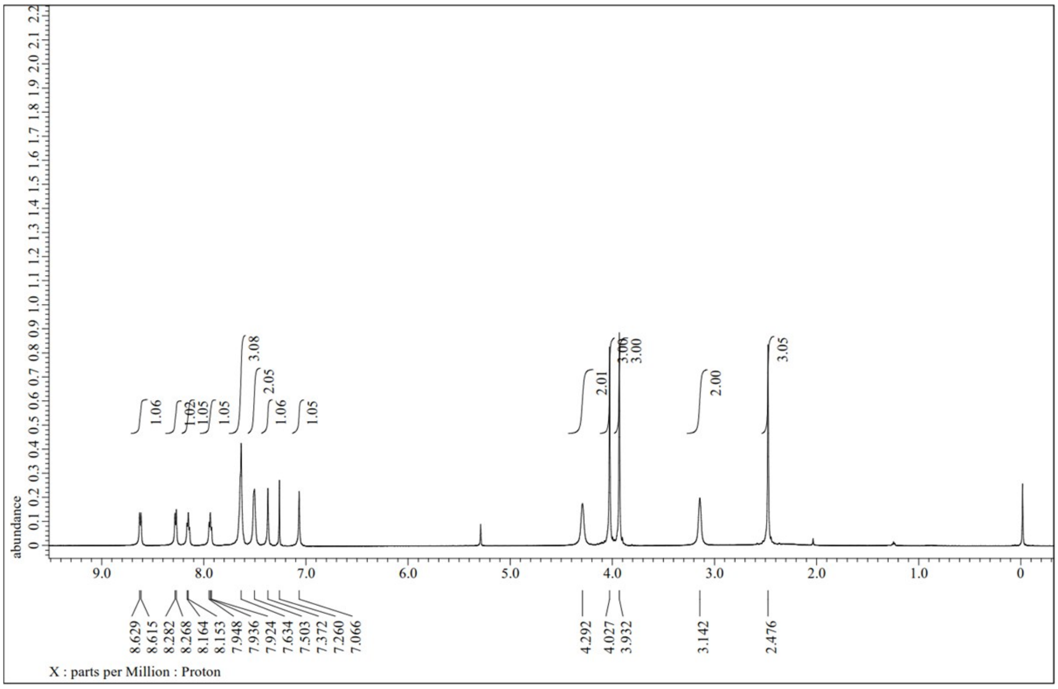
abundance
1.06
1.02
1.05
1.05
3.08
2.05
1.06
1.05
2.01
3.00
3.00
2.00
3.05
8.629
8.615
8.282
8.268
8.164
8.153
7.948
7.936
7.924
7.634
7.503
7.372
7.260
7.066
4.292
4.027
3.932
3.142
2.476
X : parts per Million : Proton

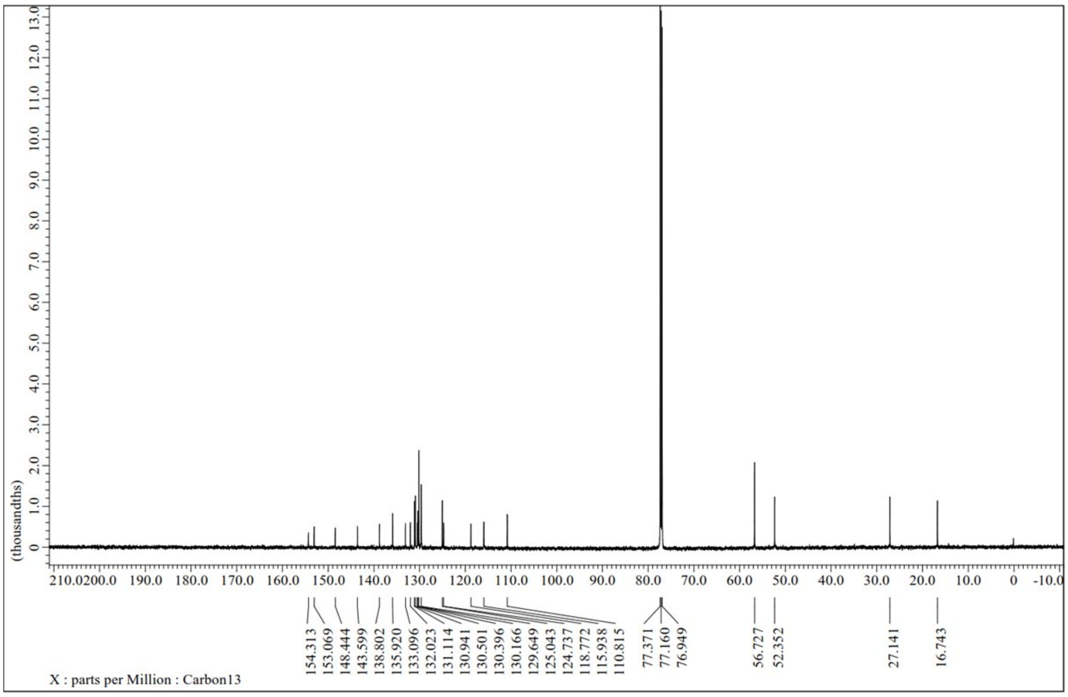
(thousandths)
154.313
153.069
148.444
143.599
138.802
135.920
133.096
132.023
131.114
130.941
130.501
130.396
130.166
129.649
125.043
124.737
118.772
115.938
110.815
77.371
77.160
76.949
56.727
52.352
27.141
16.743
X : parts per Million : Carbon13

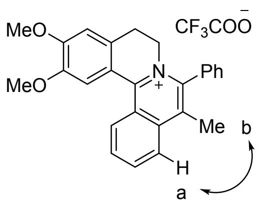

3ah

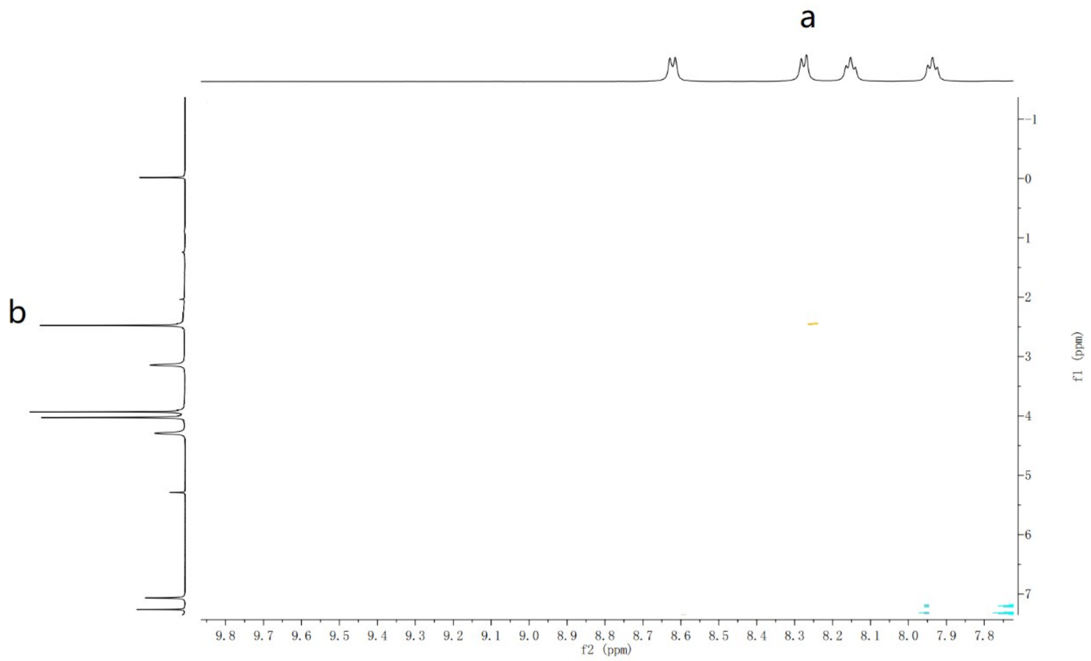

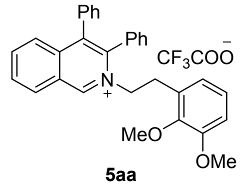
Ph
Ph
CF3COO
N
MeO
OMe

5aa

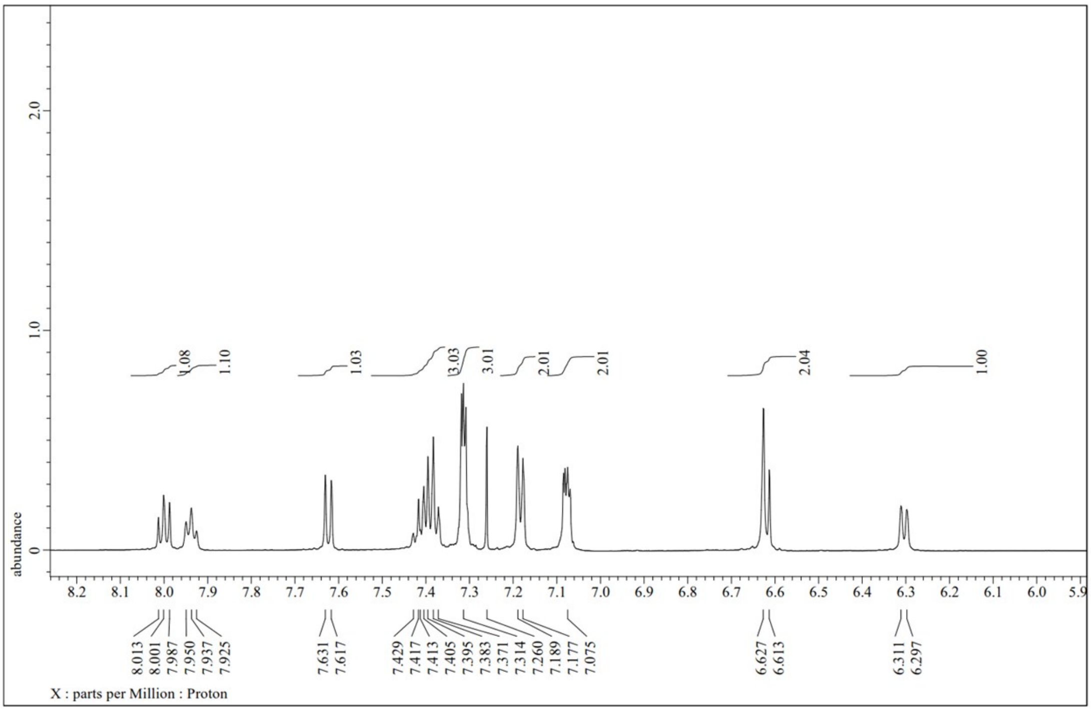
abundance
1.08
1.10
1.03
3.03
3.01
2.01
2.01
2.04
1.00
8.013
8.001
7.987
7.950
7.937
7.925
7.631
7.617
7.429
7.417
7.413
7.405
7.395
7.383
7.371
7.314
7.260
7.189
7.177
7.075
6.627
6.613
6.311
6.297
X : parts per Million : Proton

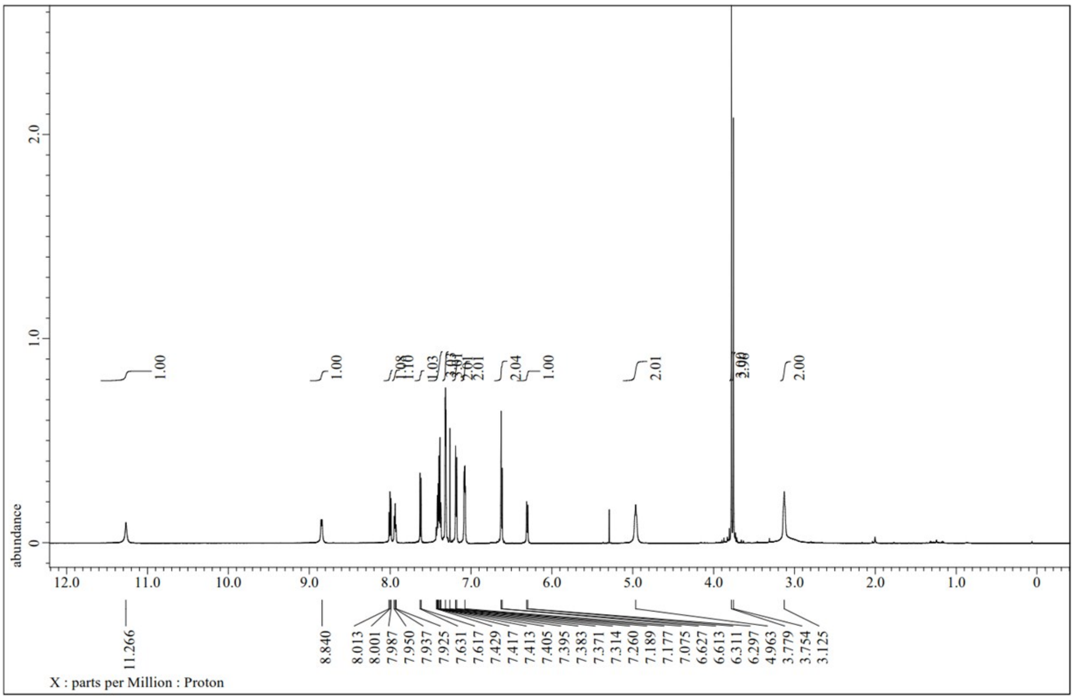
abundance
1.00
1.00
2.01
2.00
11.266
8.840
8.013
8.001
7.987
7.950
7.937
7.925
7.631
7.617
7.429
7.417
7.413
7.405
7.395
7.383
7.371
7.314
7.260
7.189
7.177
7.075
6.627
6.613
6.311
6.297
4.963
3.779
3.754
3.125
X : parts per Million : Proton

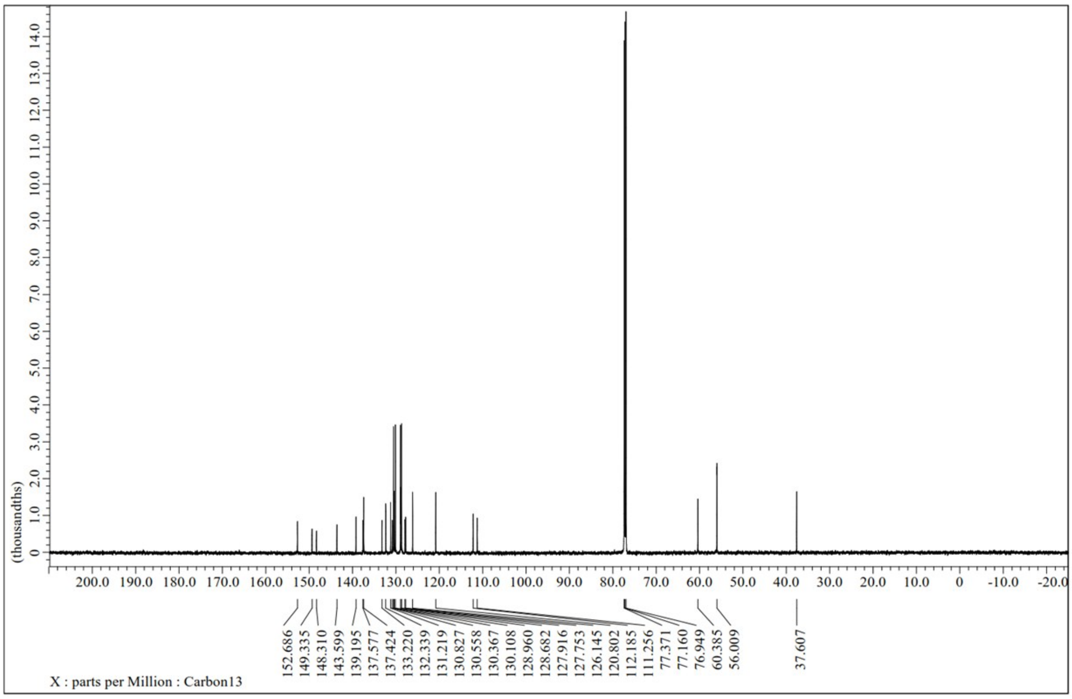
(thousandths)
X : parts per Million : Carbon13
152.686
149.335
148.310
143.599
139.195
137.577
137.424
133.220
132.339
131.219
130.827
130.558
130.367
130.108
128.960
128.682
127.916
127.753
126.145
120.802
112.185
111.256
77.371
77.160
76.949
60.385
56.009
37.607

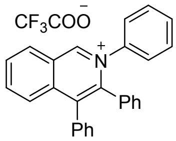
CF3COO
Ph
Ph

5ba

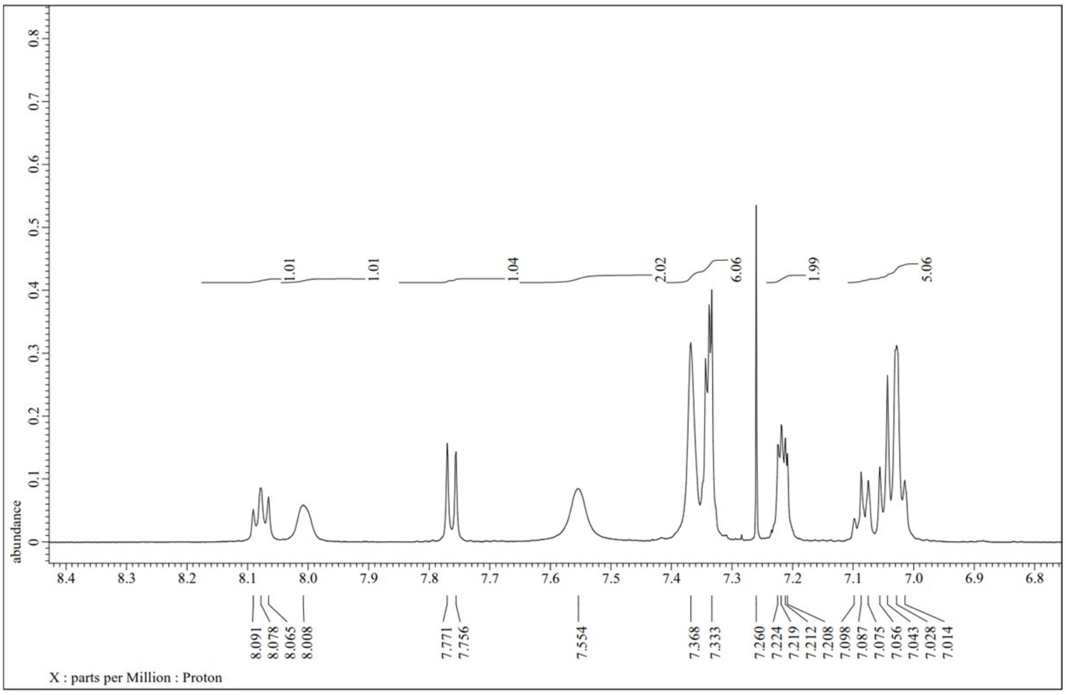
abundance
X : parts per Million : Proton
1.01
1.01
1.04
2.02
6.06
1.99
5.06
8.091
8.078
8.065
8.008
7.771
7.756
7.554
7.368
7.333
7.260
7.224
7.219
7.212
7.208
7.098
7.087
7.075
7.056
7.043
7.028
7.014

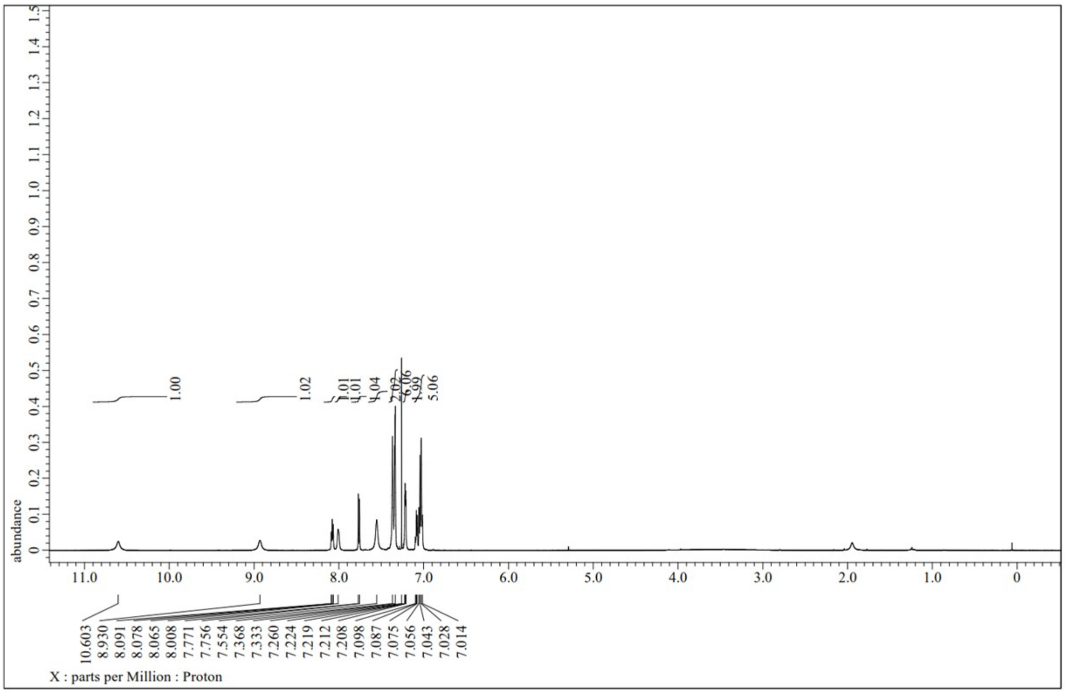
abundance
10.603
8.930
8.091
8.078
8.065
8.008
7.771
7.756
7.554
7.368
7.333
7.260
7.224
7.219
7.212
7.208
7.098
7.087
7.075
7.056
7.043
7.028
7.014
X : parts per Million : Proton

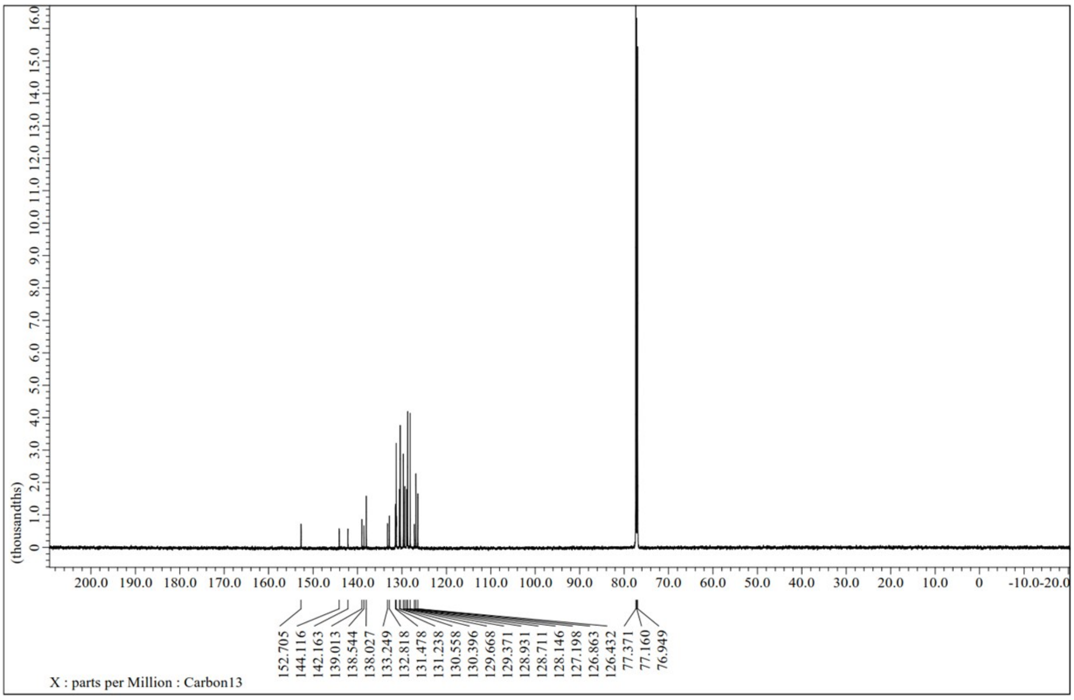
(thousandths)
152.705
144.116
142.163
139.013
138.544
138.027
133.249
132.818
131.478
131.238
130.558
130.396
129.668
129.371
128.931
128.711
128.146
127.198
126.863
126.432
77.371
77.160
76.949
X : parts per Million : Carbon13

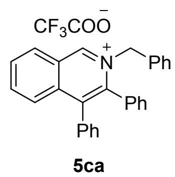
CF3COO
Ph
Ph
Ph

5ca

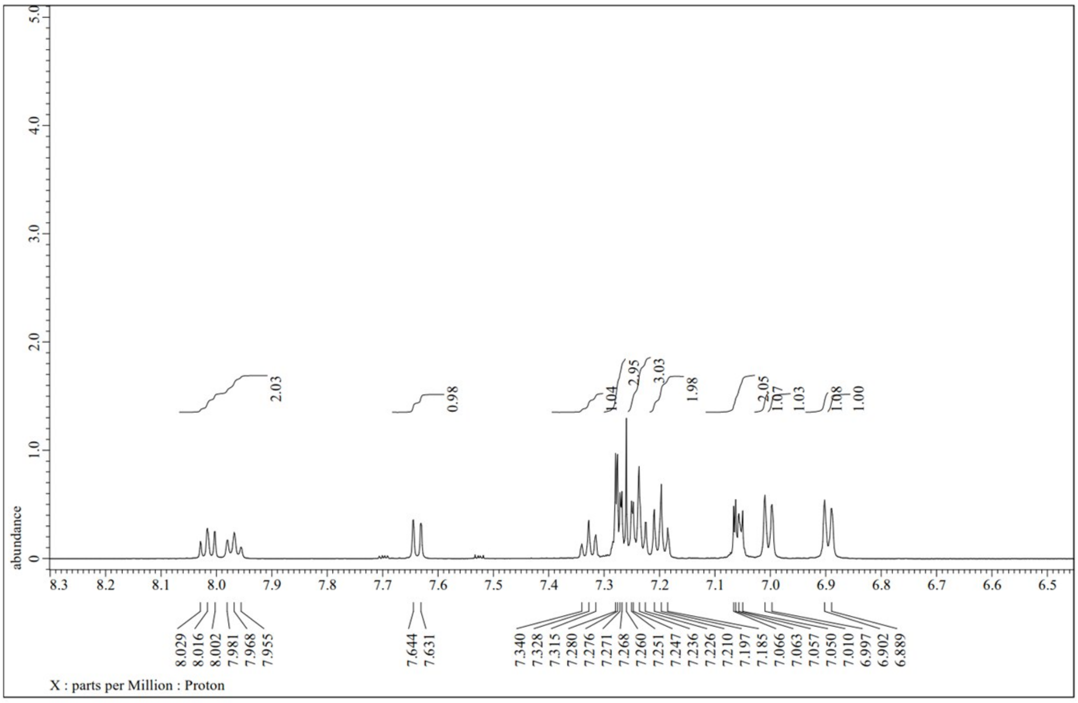
abundance
X : parts per Million : Proton

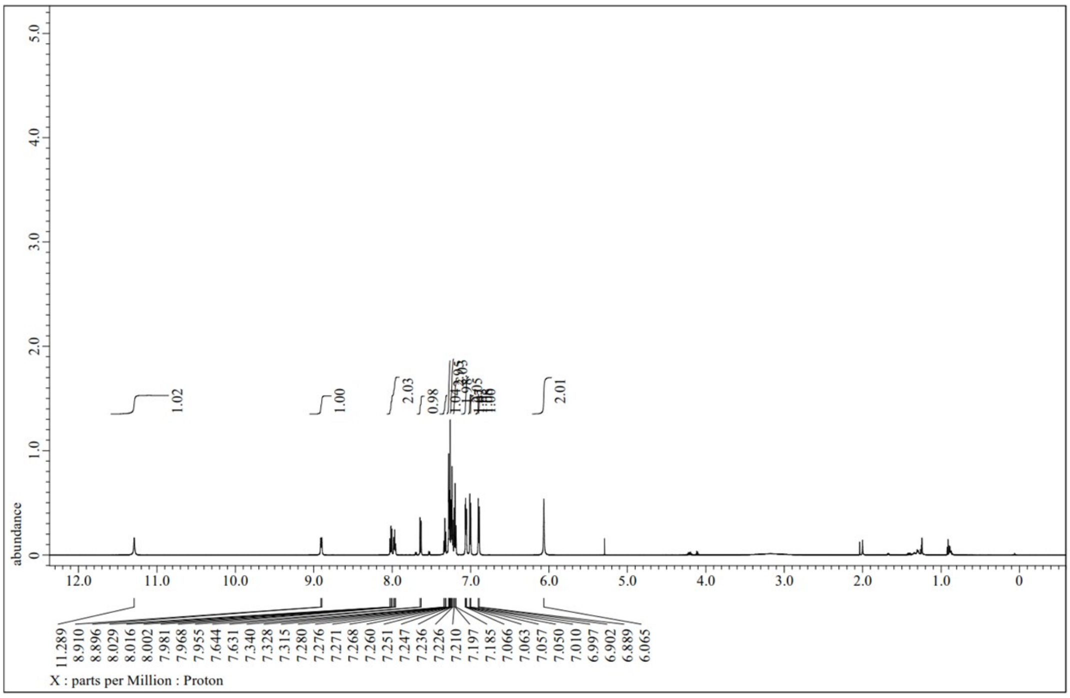
abundance
X : parts per Million : Proton

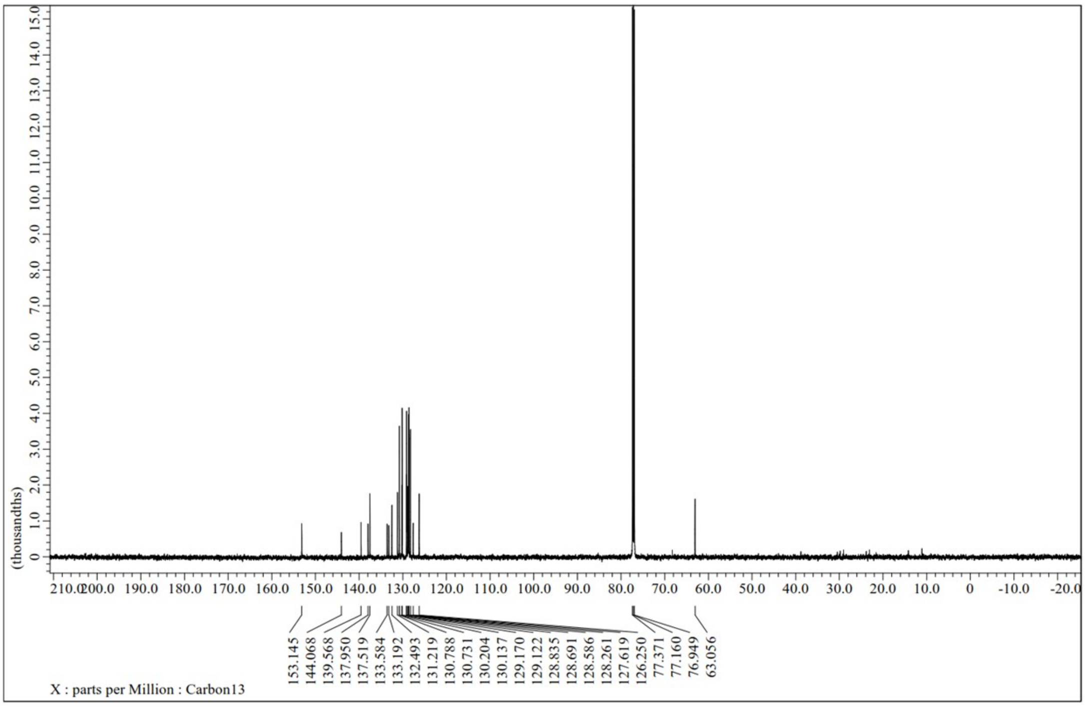

7. Crystal Data

Table S3. Crystal data and structure refinement for 3ba

Empirical formula	$C_{34}H_{28}F_3NO_5$
Formula weight	587.57
Temperature/K	293.15
Crystal system	triclinic
Space group	P-1
a/Å	10.8707(6)
b/Å	12.3194(7)
c/Å	13.1341(8)
α/°	87.958(5)
β/°	74.654(5)
γ/°	70.533(5)
Volume/Å^3	1596.56(17)
Z	2
ρ_{calc}g/cm^3	1.222
μ/mm^{-1}	0.093
F(000)	612.0
Crystal size/mm3	0.35 × 0.3 × 0.25
Index ranges	$-10 \le h \le 13$, $-15 \le k \le 14$, $-16 \le l \le 16$
Reflections collected	12890
Independent reflections	6531 [R_{int} = 0.0206, R_{sigma} = 0.0439]
Data/restraints/parameters	6531/0/401
Goodness-of-fit on F^2	1.113
Final R indexes [I>=2σ (I)]	R_1 = 0.0680, wR_2 = 0.2025

Final R indexes [all data]	R_1 = 0.1069, wR_2 = 0.2310
Largest diff. peak/hole / e Å^{-3}	0.37/-0.33

Table S4. Bond Lengths for 3ba

Atom	Atom	Length/Å	Atom	Atom	Length/Å
O1	C1	1.353(3)	C18	C19	1.354(3)
O1	C10	1.423(4)	C18	C21	1.491(3)
O2	C2	1.351(3)	C19	C27	1.493(3)
O2	C11	1.428(3)	C21	C22	1.353(4)
O3	C15	1.360(3)	C21	C26	1.355(4)
O3	C20	1.415(3)	C22	C23	1.385(4)
N1	C5	1.348(3)	C23	C24	1.343(5)
N1	C6	1.472(3)	C24	C25	1.356(6)
N1	C19	1.394(3)	C25	C26	1.393(5)
C1	C2	1.405(3)	C27	C28	1.362(3)
C1	C9	1.380(3)	C27	C32	1.371(4)
C2	C3	1.374(3)	C28	C2	1.390(4)
C3	C4	1.406(3)	C29	C30	1.366(5)
C4	C5	1.465(3)	C30	C3	1.364(5)
C4	C8	1.386(3)	C31	C32	1.378(4
C5	C12	1.420(3)	O4	C33	1.210(4)
C6	C7	1.473(4)	O5	C33	1.207(4)
C7	C8	1.481(3)	C33	C34	1.518(5)
C8	C9	1.387(3)	C34	F	1.347(5)
C12	C13	1.402(3)	C34	F2	1.331(6)
C12	C17	1.419(3	C34	F3	1.316(6)
C13	C14	1.364(3)	C34	F1A	1.278(15)
C14	C15	1.393(3)	C34	F2A	1.083(17)
C15	C16	1.361(3)	C34	F3A	1.438(13)
C16	C17	1.405(3)	F1A	F3A	1.75(2)
C17	C18	1.431(3)			

Table S5. Bond Angles for 3ba

Atom	Atom	Atom	Angle/˚	Atom	Atom	Atom	Angle/˚
C1	O1	C10	117.2(2)	C19	C18	C17	119.0(2)
C2	O2	C11	117.42(19)	C19	C18	C21	121.4(2)
C15	O3	C20	118.4(2)	N1	C19	C27	116.57(19)
C5	N1	C6	119.10(19)	C18	C19	N1	120.6(2)
C5	N1	C19	122.22(18)	C18	C19	C27	122.8(2)
C19	N1	C6	118.67(18)	C22	C21	C18	122.1(3)

O1	C1	C2	115.2(2)	C22	C21	C26	118.5(3)
O1	C1	C9	125.0(2)	C26	C21	C18	119.4(2)
C9	C1	C2	119.7(2)	C21	C22	C23	121.2(3)
O2	C2	C1	115.1(2)	C24	C23	C22	120.3(3)
O2	C2	C3	125.2(2)	C23	C24	C25	119.4(3)
C3	C2	C1	119.7(2)	C24	C25	C26	120.3(4)
C2	C3	C4	120.6(2)	C21	C26	C25	120.4(3)
C3	C4	C5	121.7(2)	C28	C27	C19	122.0(2)
C8	C4	C3	119.3(2)	C28	C27	C32	119.1(3)
C8	C4	C5	118.8(2)	C32	C27	C19	118.9(2)
N1	C5	C4	117.48(19)	C27	C28	C29	120.5(3)
N1	C5	C12	118.32(19)	C30	C29	C28	119.5(3)
C12	C5	C4	124.2(2)	C31	C30	C29	120.5(3)
N1	C6	C7	112.1(2)	C30	C31	C32	119.4(3)
C6	C7	C8	109.6(2)	C27	C32	C31	120.9(3)
C4	C8	C7	116.4(2)	O4	C33	C34	113.6(3)
C4	C8	C9	120.1(2)	O5	C33	O4	131.4(4)
C9	C8	C7	123.5(2)	O5	C33	C34	115.0(4)
C1	C9	C8	120.6(2)	F1	C34	C33	113.1(3)
C13	C12	C5	122.5(2)	F2	C34	C33	116.5(4)
C13	C12	C17	118.3(2)	F2	C34	F1	97.6(4)
C17	C12	C5	119.0(2)	F3	C34	C33	110.6(4)
C14	C13	C12	121.6(2)	F3	C34	F1	100.5(4)
C13	C14	C15	119.5(2)	F3	C34	F2	116.7(4)
O3	C15	C14	123.3(2)	F1A	C34	C33	122.1(6)
O3	C15	C16	115.8(2)	F1A	C34	F3A	80.2(9)
C16	C15	C14	120.9(2)	F2A	C34	C33	124.1(11)
C15	C16	C17	120.6(2)	F2A	C34	F1A	107.4(12)
C12	C17	C18	119.2(2)	F2A	C34	F3A	107.0(11)
C16	C17	C12	118.9(2)	F3A	C34	C33	105.5(5)
C16	C17	C18	121.9(2)	C34	F1A	F3A	53.9(6)
C17	C18	C21	119.6(2)	C34	F3A	F1A	45.9(7)
